# A fair bed allocation during COVID-19 pandemic using TOPSIS technique based on correlation coefficient for interval-valued pythagorean fuzzy hypersoft set

**DOI:** 10.1038/s41598-024-53923-2

**Published:** 2024-04-01

**Authors:** Rana Muhammad Zulqarnain, Wen-Xiu Ma, Imran Siddique, Hijaz Ahmad, Sameh Askar

**Affiliations:** 1 School of Mathematical Sciences, Zhejiang Normal University, Jinhua, 321004 Zhejiang China; 2 Department of Mathematics, University of Sargodha, Sargodha, 40100 Pakistan; 3 Section of Mathematics, International Telematic University Uninettuno, Corso Vittorio Emanuele II, 39, 00186 Rome, Italy; 4 Department of Statistics and Operations Research, College of Science, King Saud University, P.O. Box 2455, 11451 Riyadh, Saudi Arabia

**Keywords:** Pythagorean fuzzy hypersoft set, Hypersoft set, Correlation coefficient, Weighted correlation coefficient, TOPSIS, MADM, COVID-19, Bed allocation, Mathematics and computing, Applied mathematics, Computational science

## Abstract

The relationship between two variables is an essential factor in statistics, and the accuracy of the results depends on the data collected. However, the data collected for statistical analysis can be unclear and difficult to interpret. One way to predict how one variable will change about another is by using the correlation coefficient (CC), but this method is not commonly used in interval-valued Pythagorean fuzzy hypersoft set (IVPFHSS). The IVPFHSS is a more advanced and generalized form of the Pythagorean fuzzy hypersoft set (PFHSS), which allows for more precise and accurate analysis. In this research, we introduce the correlation coefficient (CC) and weighted correlation coefficient (WCC) for IVPFHSS and their essential properties. To demonstrate the applicability of these measures, we use the COVID-19 pandemic as an example and establish a prioritization technique for order preference by similarity to the ideal solution (TOPSIS) model. The technique is used to study the problem of optimizing the allocation of hospital beds during the pandemic. This study provides insights into the importance of utilizing correlation measures for decision-making in uncertain and complex situations like the COVID-19 pandemic. It is a robust multi-attribute decision-making (MADM) methodology with significant importance. Subsequently, it is planned to increase a dynamic bed allocation algorithm based on biogeography to accomplish the superlative decision-making system. Moreover, numerical investigations deliberate the best decision structures and deliver sensitivity analyses. The efficiency of our encouraged algorithm is more consistent than prevalent models, and it can effectively control and determine the optimal configurations for the study.

## Introduction

A correlation coefficient is a statistical measure that is commonly used to evaluate the degree of relationship between two variables. In industrial engineering, correlation coefficients are commonly used to analyze the relationships between different factors that affect the performance of a system. For example, in manufacturing, a CC can be used to determine how the machine speed, the number of workers, or other factors affect the production rate. However, using probabilistic methods to analyze real-world industrial problems can be challenging due to the large amount of arbitrary data involved. Complex systems often have imprecise uncertainties that make it difficult to obtain accurate probability estimates. In addition, there may be insufficient data to correct the information obtained from these methods. So, outcomes based on probability models are not always helpful to professionals. To overcome these limitations, multiple attribute decision-making (MADM) has been identified as a more effective way to make appropriate decisions when uncertainties and imperfections are present. To report these issues, Zadeh^1^ anticipated the concept of fuzzy sets (FS), which can help condense redundant and apprehensive data. Turksen^2^ also offered the idea of interval value fuzzy sets (IVFS), which represent membership values as intervals rather than numbers, making them better suited for describing uncertainty in complex systems. Using an IVFS in fuzzy control is important to make accurate decisions in complex situations. The fuzzy TOPSIS approach was established by Chen^3^ to find the closeness coefficient, which was further prolonged by Ashtiani et al.^4^ to determine multi-criteria decision-making (MCDM) challenges in the IVFS setting considering the membership degrees (MD).

However, traditional FS and IVFS have limitations in handling non-membership degrees (NMD) in decision-making (DM) assessments. Atanassov^5^ protracted the intuitionistic fuzzy sets (IFS) contracting theory with the abovementioned drawbacks. The TOPSIS method has been used in green supply chain management for IFS by Rouyendegh et al.^6^. Atanassov^7^ extended his IFS theory to IVIFS. Several techniques for computing the CC of IFS and IVIFS were developed by Hung and Wu^8^, Bustince and Burillo^9^, and Mitchell^10^. The IVIFS TOPSIS has been utilized for supplier selection by Tiwari et al.^11^. The Pythagorean fuzzy set (PFS) was created by Yager^12^ to address the shortcomings of existing FS theories in handling inconsistent and uncertain data. Yager improved upon the basic condition ${\mathcal{T}} + {\mathcal{J}} \le 1$ by revising it to ${\mathcal{T}}^{2} + {\mathcal{J}}^{2} \le 1$ to correct errors in the system. Since then, other researchers have built upon Yager's work in PFS theory. Biswas & Sarkar^13^ proposed the TOPSIS model for PFS to resolve multi-criteria group decision-making (MCGDM) complexities. Einstein-weighted geometric aggregation operators (AOs) for multi-attribute group decision-making (MAGDM). In recent years, Pythagorean fuzzy sets (PFS) have emerged as a powerful tool in DM. In this context, Wei and Lu^14^ proposed using power AOs to solve MADM problems in PFS. Similarly, Wang and Li^15^ investigated using power Bonferroni mean operators with Pythagorean fuzzy numbers (PFNs) to explore their interaction and potential applications in DM. Zhang^16^ proposed a DM method that uses similarity measures to address multi-criteria group decision-making (MCGDM) obstacles in PFS scenarios. Peng and Yang^17^ extended the PFS theory to include interval-valued Pythagorean fuzzy sets (IVPFS), which allowed for the development of a DM system based on their method. Garg^18^ presented a novel score function for IVPFS and proposed the TOPSIS technique using his developed function. 

Despite these advancements, there are still limitations in dealing with uncertainties and vagueness in parametric chemistry. Nonetheless, PFS theory and its extensions have proved useful tools for DM in various fields. Existing methods often struggle with handling uncertainties and vagueness in parametric chemistry, which can be ambiguous, obscure, or equivocal. Researchers have been exploring new mathematical tools and theories to overcome these limitations. One of these is the soft set (SS) introduced by Molodtsov^19^. This general mathematical tool has proven useful in addressing vague or uncertain problems. Maji et al.^20^ combined FS and SS to create fuzzy soft sets (FSS) to build upon the SS theory. Maji et al.^21^ extended this intuitionistic fuzzy soft set (IFSS) with essential operations and properties. In addition to these developments, Garg and Arora^22^ proposed a leeway of the TOPSIS method based on correlation coefficients (CC). This extension addressed the complexities involved in MADM. Jiang et al.^23^ introduced an extension to the IFSS called interval-valued IFSS (IVIFSS). They presented fundamental operations for IVIFSS to address uncertainties and vagueness in DM problems. Ma et al.^24^ utilized the choice and score values of IVIFSS and proposed an innovative DM method to handle complex problems that involve multiple criteria. The proposed method enables decision-makers to consider the importance of criteria and the alternatives' performances more efficiently. Khan et al.^25^ developed MADM approaches for generalized IVIFSS, which includes basic operations such as union, intersection, and complement, to address realistic complications. The proposed approach considers both the preference and non-preference information and provides a more comprehensive evaluation of alternatives. To deal with MADM issues in IVIFSS, Zulqarnain et al.^26^ established the TOPSIS and AOs for IVIFSS. The proposed approach enables decision-makers to handle complex DM problems involving multiple attributes, and the AOs provide flexibility in selecting the appropriate operator based on the problem's nature. Garg and Arora^27^ offered a nonlinear programming method to determine MADM convolutions under IVIFSS, considering the weight of each criterion and the degree of importance of each alternative's performance in a more effective way. The proposed method provides an optimal solution that considers the uncertainties and vagueness of the DM problem. Researchers have been exploring new mathematical tools and theories to address the limitations of existing methods in handling uncertainties and vagueness in parametric chemistry. One such tool is the Pythagorean fuzzy soft set (PFSS), which combines two existing theories, PFS and SS. Peng et al.^28^ proposed Pythagorean fuzzy soft sets (PFSSs), which combine PFS and SS and possess desirable characteristics. Athira et al.^29^ developed and used entropy and distance measures for PFSSs in DM. They also introduced new similarity and distance measures^30^ for PFSS that offer greater flexibility than IFSS or FSS. In addition to these advancements, several extensions have been made to PFSS. For instance, Riaz et al.^31,32^ developed m polar PFSS, while Hua et al.^33^ extended PFSS to possibility PFSS. Zulqarnain et al.^34^ extended the Einstein operational laws for PFSS and introduced the Einstein-ordered weighted ordered geometric AO for PFSS, which was utilized to establish a MAGDM technique. They also proposed the Einstein-ordered weighted average AO for PFSS^35^ and applied CC to settle the TOPSIS method for PFSS^36^. All of these advancements offer promising avenues for addressing the challenges posed by uncertainties and vagueness in parametric chemistry, and they demonstrate the vast potential of these tools and theories for solving multifaceted, realistic complications. Zulqarnain et al.^37^ settled AOs for IVPFSS and presented a MAGDM approach for solving real-world problems. 

When dealing with DM problems, models based on SS settings have proven useful due to their simplicity, as they only require single-parameter assessments. However, in some situations, dividing parameters into more specific subcategories may be necessary. This is where hypersoft sets (HSS)^38^ come into play, allowing for incorporating multiple sub-parameters into DM. While SS models may be sufficient in certain scenarios, they cannot differentiate parameters into subcategories. In several DM situations, it is necessary to consider parameters as sub-parameters to obtain more accurate results. Incorporating HSS into the DM process makes creating more comprehensive models considering multiple sub-parameters possible, resulting in a more accurate decision assessment. There are various approaches to HSS, each with corresponding DM methods. For example, Rahman et al.^39^ introduced the possibility IFHSS and established DM methods using similarity measures. Rahman et al.^40^ demonstrated a DM methodology for neutrosophic HSS. Zulqarnain et al.^41^ extended the TOPSIS for IFHSS and the DM methodologies using the AOs. Debnath^42^ introduced the basic operations for the interval-valued intuitionistic fuzzy hypersoft set (IVIFHSS) to regulate MCDM obstacles. The correlation-based TOPSIS method is introduced in^43^ to choose the most appropriate thermal energy storage technique. Zulqarnain et al.^44^ extended IFHSS to PFHSS and presented the fundamental operations. Zulqarnain et al.^45^ raised the AOs in the IVPFHSS setting and demonstrated an MCDM technique to resolve DM complications.

Recently, the spread of major transmissible viruses has posed a main risk to worldwide human health, life safety, and monetary evolution. The COVID-19 pandemic has exaggerated the world, with millions of inveterate cases and demises reported by the World Health Organization. The rapid spread of the virus has led to a shortage of hospital beds, making it difficult to provide proper care for both COVID-19 patients and non-COVID-19 patients. This has increased the threat of infection transmission and persistent decease, highlighting the urgent need for hospital administrators to allocate beds properly. In response to this need, our work aims to develop an efficient and effective bed allocation technique during the COVID-19 pandemic to ensure that hospitals can adequately treat patients while preventing the virus's spread. Due to the time-varying and highly uncertain needs of hospital resource requirements, hospital managers face the challenge of balancing the limited assets of dissimilar kinds of zones and patients. Research on cross-infection prevention in infectious disease hospitalizations in Singapore and Italy has focused on three categories: placing patients at risk of COVID-19 in isolation beds to manage isolated patients^46,47^. In response to the COVID-19 pandemic, hospitals have implemented various measures to prevent the spread of the virus and ensure patient safety. One such measure is providing each patient with personal protective equipment (PPE) upon admission. This is important in preventing the transmission of the virus from infected patients to others in the hospital. In addition, various hospitals have set up bumper zones in reserve rooms, passable rooms, or common wards^48^. These buffer wards are used to separate new patients hospitalized for a specific period and screen COVID-19 patients to prevent the spread of the virus. This methodology has been implemented in countries like Egypt and China^49,50^ and has proven effective in reducing the risk of cross-infection and improving patient outcomes. But, the first preference can clue to a severe deficiency of isolation beds, and the second route, despite the endowment of surplus particular protecting equipment to hospitalized patients, can quiet chief to nosocomial infection.

On the contrary, asymptomatic and latent COVID-19 patients can be successfully recognized by patients' opinions in a buffer ward. During the COVID-19 pandemic, there has been a surge in demand for hospital services, leading to a strain on resources and medical staff. To manage this situation, hospitals have implemented various measures to prioritize COVID-19 patients while ensuring that non-COVID-19 patients receive timely and appropriate treatment. One such measure is using buffer wards, designated areas within hospitals where patients are monitored and treated for a specific period. To improve DM processes and address flaws, an integrated approach is needed. However, spies may be hesitant to share information due to concerns about security and confidentiality. This can lead to incomplete or asymmetrical information, further complicating the DM process. Considering all available information is emphasized to tackle this issue, and the concept of IVPFHSS was introduced. IVPFHSS is a mathematical tool that enables decision-makers to account for uncertainty and vagueness in the data and make informed decisions by considering the full range of possibilities. By using IVPFHSS, decision-makers can consider a wide range of factors and assess the importance of each in the overall DM process. This helps create a comprehensive structure for DM that considers all relevant information and provides a more accurate picture of the situation.

### Research motivation

IVPFHSS sets incorporate higher levels of uncertainty and frequently encourage a higher degree of vagueness. To effectively deal with convoluted inconsistency during human cognitive and DM projects, the effective TOPSIS approach, which has formerly concentrated on particular contexts and fuzzy settings, needs to be extended to the IVPFHSS environment. As the TOPSIS strategy is a highly competent method for handling DM procedures, it has gained limited application in medical DM and healthcare facilities. IVPFHSS is a tool that utilizes HSS and IVPFS to assist in managing inconsistencies, variations, and inaccurate data. TOPSIS plays an important role in DM challenges for integrating disparate aims into an organized assessment.

On the other hand, the currently available TOPSIS strategy has limitations in that it cannot measure composite interval-valued Pythagorean fuzzy hypersoft numbers (IVPFHSNs). The CC and WCC have been modified to reflect the specific circumstances of IVPFHSS better. Because of this improvement, the model suggested has grown better than conventional TOPSIS methodologies. Still, the previous approach’s outcomes are unfavourable, and assessing the bias toward the more favourable model can be complicated. Prior studies showed that the TOPSIS strategy, particularly^26^, can’t be sufficiently flexible for delivering beneficial concepts and reliable outcomes since it cannot identify sub-attributes of a possible solution, reducing its efficiency. Furthermore, the TOPSIS approach described in^43^ can understand various alternative sub-attributes, making it more flexible and productive. Moreover, the model does not differentiate between different MD (NMD) functionality levels in the overall development.

Consequently, there is a bias towards the alternatives, making it challenging to determine their suitability partiality. TOPSIS will also be developed for MADM obstacles, employing the correlation measures produced to resolve these obstacles. The suggested strategy improvement has culminated in a more pragmatic methodology for navigating interval form alternatives in the IVPFHSS area. It is because of alterations performed to the CC and WCC that enables the framework to become more adaptable and more capable of delivering reliable outcomes. Despite these developments, the present approach continues to face difficulties in establishing partiality for the different approaches, and the outcomes are not always favourable. The correlation coefficient, a well-known statistical measure, relates to IVPFHSS information in this research. It is an innovative utilization of the correlation coefficient that has never been explored in this setting. This study enriches our comprehension of the interactions among several factors in IVPFHSS information by employing this measure. In contrast, our suggested TOPSIS approach takes into account sub-attributes in interval form and can manage a large number of alternative sub-attributes. The suggested approach performs MADM complications more effectively and equitably.

#### Main contributions

The most recent decision-making (DM) research emphasized the significance of integrating unpredictability and insufficient information during DM procedures. It is due to real-world issues commonly including insufficient or undetermined information, keeping sound choices impossible. A significant approach for overcoming such obstacles is IVPFHSS, which integrates the favourable aspects of both HSS and IVPFS. The frequently applied CC measure can not specifically mimic the viable assessment of alternatives in DM protocols utilizing IVPFHSS since it fails to interpret for assured aspects. IVPFHSS is a systematic technique for dealing with hesitancy, discrepancies, and incomplete data. To overcome this issue, this study aims to develop new CC and weighted CC (WCC) metrics based on imprecise data in the context of IVPFHSS. The following are the study's objectives:

- One of the major achievements of this investigation includes a structure for examining the informational energies that comprise IVPFHSS circumstances. These energies indicate how much knowledge is contained within an FS, and determining these individuals is important to developing appropriate correlation measures.
- The researchers used informational energies and correlation measures to establish the CC and WCC measures stated in this research have been optimized for IVPFHSS. Within the IVPFHSS structure, these measures are intended to measure the associations among various characteristics or parameters in a more precise way. This innovation expands the available statistical approaches for interpreting and analyzing IVPFHSS information. The requirements mentioned above consider the insufficient information in IVPFHSS to deliver an improved exact assessment of the real significance of substitutes in DM techniques.
- Based on the presented correlation measures (CC and WCC), this study constructs a TOPSIS technique using IVPFHSS data. This distinctive strategy helps decision-makers examine and prioritize alternatives in MADM challenges properly. The combination of TOPSIS and the suggested correlation measures boosts the decision-making procedure's precision as well as dependability.
- This study demonstrates that integrating the correlation measures and TOPSIS in the proposed method contributed to a reliable MADM strategy. This technique offers significant benefits by offering decision-makers an entire structure for evaluating and assigning priority to prospects in the environment of IVPFHSS. This strategy's adaptability promotes the reliability as well as the efficacy of decision-making procedures in several kinds of fields. Moreover, we utilized it in evaluating DM distraction and bed allocation during covid 19 pandemic, and picking the most practical for benchmarking.
- Comparative analyses have been presented to ensure the feasibility of the developed TOPSIS methodology, demonstrating that the suggested method is more productive and effective than existing TOPSIS methodologies. This study seeks to add to the field of DM under uncertainty by providing these new techniques and supporting decision-makers to make better-informed and more precise choices.

In Section “Introduction”, the introduction provides an overview of the need for a new TOPSIS method for handling uncertainty and incomplete information. Section “Preliminaries” outlines the basic concepts that support the research, including an overview of IVPFHSS and the existing TOPSIS technique. Section “Correlation coefficient for interval valued pythagorean fuzzy hypersoft set” focuses on introducing informational energies for IVPFHSS and developing CC correlation measures with significant properties. In section “Weighted correlation coefficient for interval valued pythagorean fuzzy hypersoft set”, the WCC correlation measure is introduced, along with its crucial properties. In section “Proposed TOPSIS approach based on correlation coefficient to resolve MADM problem under IVPFHSS”, the paper explains how the TOPSIS technique has been developed using newly developed correlation measures to solve multiple attribute decision-making (MADM) problems. This section details the methodology employed to develop the TOPSIS technique and how it addresses the limitations of the existing MADM techniques. Section “I. Challenges in implementing fair bed allocation policies during the COVID-19 Pandemic” presents a numerical exploration that aims to determine the bed allocation for the most critical patients of COVID-19. Finally, section “Discussion and comparative analysis” provides a comparative analysis of the proposed model to ensure its pragmatism. The section compares the performance of the proposed model with other existing models and highlights the advantages of the proposed model. The comparative analysis also includes a discussion of the limitations of the proposed model and potential avenues for future research.

## Preliminaries

This section will discuss some basic concepts necessary to understand our research’s structure and organization. These concepts will provide a foundation for the topics and ideas presented later in the paper. By recalling these fundamental notions, we hope to provide a clear and concise overview of the background and context of our research.

### Definition 1

^17^ An interval-valued Pythagorean fuzzy set ${\mathfrak{T}}$ over a universe of discourse ${U}$, such as ${\mathfrak{T}} \subseteq {U}$ it is defined as

$${\mathfrak{T}} = \left\{ {\left. { \left( {{\mathfrak{u}}_{i},\left( {{\mathcal{T}}_{{{\mathfrak{T}}_{j} }} \left( {{\mathfrak{u}}_{i} } \right), {\mathcal{J}}_{{{\mathfrak{T}}_{j} }} \left( {{\mathfrak{u}}_{i} } \right)} \right)} \right)} \right|{\mathfrak{u}}_{i} \in {U}} \right\}$$

where, ${\mathcal{T}}_{{{\mathfrak{T}}_{j} }} \left( {{\mathfrak{u}}_{i} } \right) = \left[ {{\mathcal{T}}_{{{\mathfrak{T}}_{j} }}^{\ell } \left( {{\mathfrak{u}}_{i} } \right),{ }{\mathcal{T}}_{{{\mathfrak{T}}_{j} }}^{\upsilon } \left( {{\mathfrak{u}}_{i} } \right)} \right]$ and ${\mathcal{J}}_{{{\mathfrak{T}}_{j} }} \left( {{\mathfrak{u}}_{i} } \right) = \left[ {{\mathcal{J}}_{{{\mathfrak{T}}_{j} }}^{\ell } \left( {{\mathfrak{u}}_{i} } \right),{ }{\mathcal{J}}_{{{\mathfrak{T}}_{j} }}^{\upsilon } \left( {{\mathfrak{u}}_{i} } \right)} \right]$ be the MD and NMD intervals. Also, $\left[ {{\mathcal{T}}_{{{\mathfrak{T}}_{j} }}^{\ell } \left( {{\mathfrak{u}}_{i} } \right),{ }{\mathcal{T}}_{{{\mathfrak{T}}_{j} }}^{\upsilon } \left( {{\mathfrak{u}}_{i} } \right)} \right] \subseteq \left[ {0, 1} \right]$ and $\left[ {{\mathcal{J}}_{{{\mathfrak{T}}_{j} }}^{\ell } \left( {{\mathfrak{u}}_{i} } \right),{ }{\mathcal{J}}_{{{\mathfrak{T}}_{j} }}^{\upsilon } \left( {{\mathfrak{u}}_{i} } \right)} \right] \subseteq \left[ {0, 1} \right]$, $0 \le {\mathcal{T}}_{{{\mathfrak{T}}_{j} }}^{\ell } \left( {{\mathfrak{u}}_{i} } \right),{ }{\mathcal{T}}_{{{\mathfrak{T}}_{j} }}^{\upsilon } \left( {{\mathfrak{u}}_{i} } \right), {\mathcal{J}}_{{{\mathfrak{T}}_{j} }}^{\ell } \left( {{\mathfrak{u}}_{i} } \right),{ }{\mathcal{J}}_{{{\mathfrak{T}}_{j} }}^{\upsilon } \left( {{\mathfrak{u}}_{i} } \right) \le 1$, such as $0 \le \left( {{\mathcal{T}}_{{{\mathfrak{T}}_{j} }}^{\upsilon } \left( {{\mathfrak{u}}_{i} } \right)} \right)^{2} + \left( {{\mathcal{J}}_{{{\mathfrak{T}}_{j} }}^{\upsilon } \left( {{\mathfrak{u}}_{i} } \right)} \right)^{2} \le 1$.

### Definition 2

^19^ A soft set over a universe of discourse ${U}$ and a set of attributes $\varsigma$ is a pair $\left( {\Lambda,{\mathfrak{T}}} \right)$ where $\Lambda$ is a mapping from ${\mathfrak{T}}$ to the power set of ${U}$, denoted as $P\left( {U} \right)$ and defined as follows:

$$\left( {\Lambda ,{\mathfrak{T}}} \right) = \left\{ {\Lambda \left( \varsigma \right) \in {\mathcal{P}}\left( {U} \right):\varsigma \in \varsigma,\Lambda \left( \varsigma \right) = \emptyset if \varsigma \notin {\mathfrak{T}}} \right\}$$

### Definition 3

^28^ Let ${U}$ and $\varsigma$ be a universe of discourse and a set of attributes. Then, the Pythagorean fuzzy soft set over ${U}$ is defined as follows:

$${\mathfrak{T}}_{{{\mathfrak{u}}_{i} }} \left( {\varsigma_{j} } \right){ } = \left\{ {\left. {\left( {{\mathfrak{u}}_{i},\left( {{\mathcal{T}}_{{{\mathfrak{T}}_{j} }} \left( {{\mathfrak{u}}_{i} } \right), {\mathcal{J}}_{{{\mathfrak{T}}_{j} }} \left( {{\mathfrak{u}}_{i} } \right)} \right)} \right)} \right| {\mathfrak{u}}_{i} \in {U}} \right\}$$

where ${\mathfrak{T}}$ be a mapping such as ${\mathfrak{T}}:\varsigma \to P^{{U}}$, $P^{{U}}$ shows the subsets of Pythagorean fuzzy soft sets $\forall {\mathfrak{u}}_{i} \in {U}$ and satisfied the following conditions such as: ${\mathcal{T}}_{{{\mathfrak{T}}_{j} }} \left( {{\mathfrak{u}}_{i} } \right), {\mathcal{J}}_{{{\mathfrak{T}}_{j} }} \left( {{\mathfrak{u}}_{i} } \right) \in \left[ {0, 1} \right]$ and $0 \le \left( {{\mathcal{T}}_{{{\mathfrak{T}}_{j} }} \left( {{\mathfrak{u}}_{i} } \right)} \right)^{2} + \left( {{\mathcal{J}}_{{{\mathfrak{T}}_{j} }} \left( {{\mathfrak{u}}_{i} } \right)} \right)^{2} \le 1$.

### Definition 4

^38^ Suppose we have a universe of discourse, denoted by ${U}$, which contains $n$ elements $\left\{ {{\mathfrak{u}}_{1},{\mathfrak{u}}_{2},{\mathfrak{u}}_{3},\ldots,{\mathfrak{u}}_{n} } \right\}$, $\left( {n \ge 1} \right)$. We also have a set of attributes, denoted by $\varsigma$, which contains $m$ attributes $\left\{ {\varsigma_{1} ,{ }\varsigma_{2} ,{ }\varsigma_{3} ,{ } \ldots ,{ }\varsigma_{m} } \right\}$ indicates the set of parameters and ${\mathfrak{T}}_{i}$ be the conforming sub-parameters, such as ${\mathfrak{T}}_{i} \cap {\mathfrak{T}}_{j} = {{\varphi }}$, where $i \ne j$ and $i, j \in \left\{ {1,2,3 \ldots n} \right\}$. Let ${\mathfrak{T}}_{1} \times {\mathfrak{T}}_{2} \times {\mathfrak{T}}_{3} \times \cdots \times {\mathfrak{T}}_{n} = {\mathop {\mathfrak{T}}\limits^{{...}}} = \left\{ {\left. {\left( {\varsigma_{{1j_{1} }},\varsigma_{{2j_{2} }},\ldots,\varsigma_{{nj_{n} }} } \right)} \right|\varsigma_{{1j_{1} }} \in {\mathfrak{T}}_{1} ,\varsigma_{{2j_{2} }} \in {\mathfrak{T}}_{2},\ldots,\varsigma_{{nj_{n} }} \in {\mathfrak{T}}_{n} } \right\}$ be a collection of multi-sub-attributes, where $1 \le j_{1} \le \alpha$, $1 \le j_{2} \le \beta$, and $1 \le j_{n} \le \gamma$, and $\alpha ,\beta ,\gamma \in {\text{N}}$. A pair $\left( {\Lambda ,{\mathfrak{T}}_{1} \times {\mathfrak{T}}_{2} \times {\mathfrak{T}}_{3} \times \cdots \times {\mathfrak{T}}_{n} = \left( {\Lambda, {\mathop {\mathfrak{T}}\limits^{{...}}} } \right)} \right)$ is entitled as HSS, and its mapping is defined as:

$$\Lambda :{\mathfrak{T}}_{1} \times {\mathfrak{T}}_{2} \times {\mathfrak{T}}_{3} \times \cdots \times {\mathfrak{T}}_{n} = {\mathop {\mathfrak{T}}\limits^{{...}}} \to {\mathcal{P}}\left( {U} \right)$$.

Also, it can be defined as.

$$\left( {\Lambda, {\mathop {\mathfrak{T}}\limits^{{...}}} } \right)$$ = $$\left\{ {\widehat{{\varsigma_{ij} }}, \Lambda_{{ {\mathop {\mathfrak{T}}\limits^{{...}}} }} \left( {\widehat{{\varsigma_{ij} }}} \right): \widehat{{\varsigma_{ij} }} \in {\mathop {\mathfrak{T}}\limits^{{...}}} , \Lambda_{{ {\mathop {\mathfrak{T}}\limits^{{...}}} }} \left( {\widehat{{\varsigma_{ij} }}} \right) \in {\mathcal{P}}\left( {U} \right)} \right\}$$.

### Definition 5

^42^ Suppose we have a universe of discourse, denoted by ${U}$, which contains $n$ elements $\left\{ {{\mathfrak{u}}_{1},{\mathfrak{u}}_{2},{\mathfrak{u}}_{3},\ldots,{\mathfrak{u}}_{n} } \right\}$, $\left( {n \ge 1} \right)$. We also have a set of attributes, denoted by $\varsigma$, which contains $m$ attributes $\left\{ {\varsigma_{1} ,{ }\varsigma_{2} ,{ }\varsigma_{3} ,{ } \ldots ,{ }\varsigma_{m} } \right\}$ denotes the set of attributes and ${\mathfrak{T}}_{i}$ be the conforming sub-attributes, such as ${\mathfrak{T}}_{i} \cap {\mathfrak{T}}_{j} = {{\varphi }}$, where $i \ne j$ and $i, j \in \left\{ {1,2,3 \ldots n} \right\}$. Let ${\mathfrak{T}}_{1} \times {\mathfrak{T}}_{2} \times {\mathfrak{T}}_{3} \times \cdots \times {\mathfrak{T}}_{n} = {\mathop {\mathfrak{T}}\limits^{{...}}} = \left\{ {\left. {\left( {\varsigma_{{1j_{1} }},\varsigma_{{2j_{2} }},\ldots,\varsigma_{{nj_{n} }} } \right)} \right|\varsigma_{{1j_{1} }} \in {\mathfrak{T}}_{1} ,\varsigma_{{2j_{2} }} \in {\mathfrak{T}}_{2},\ldots,\varsigma_{{nj_{n} }} \in {\mathfrak{T}}_{n} } \right\}$ be a collection of multi-sub-attributes, where $1 \le j_{1} \le \alpha$, $1 \le j_{2} \le \beta$, and $1 \le j_{n} \le \gamma$, and $\alpha ,\beta ,\gamma \in {\text{N}}$. A pair $\left( {\Lambda ,{\mathfrak{T}}_{1} \times {\mathfrak{T}}_{2} \times {\mathfrak{T}}_{3} \times \cdots \times {\mathfrak{T}}_{n} = \left( {\Lambda, {\mathop {\mathfrak{T}}\limits^{{...}}} } \right)} \right)$ is entitled an IVIFHSS, and its mapping is defined as:

$$\Lambda :{\mathfrak{T}}_{1} \times {\mathfrak{T}}_{2} \times {\mathfrak{T}}_{3} \times \cdots \times {\mathfrak{T}}_{n} = {\mathop {\mathfrak{T}}\limits^{{...}}} \to IVIFS^{U}$$.

Also, it can be defined as $\left( {\Lambda, {\mathop {\mathfrak{T}}\limits^{{...}}} } \right) = \left\{ {\left( {\widehat{{\varsigma_{ij} }}, \Lambda_{{ {\mathop {\mathfrak{T}}\limits^{{...}}} }} \left( {\widehat{{\varsigma_{ij} }}} \right)} \right): \widehat{{\varsigma_{ij} }} \in {\mathop {\mathfrak{T}}\limits^{{...}}} , \Lambda_{{ {\mathop {\mathfrak{T}}\limits^{{...}}} }} \left( {\widehat{{\varsigma_{ij} }}} \right) \in IVIFS^{U} \in \left[ {0, 1} \right]} \right\}$, where $\Lambda_{{ {\mathop {\mathfrak{T}}\limits^{{...}}} }} \left( {\widehat{{\varsigma_{ij} }}} \right) = \left\{ {{\mathfrak{u}}, \left( { {\mathcal{T}}_{{\Lambda \left( {\widehat{{\varsigma_{ij} }}} \right)}} \left( {\mathfrak{u}} \right),{ }{\mathcal{J}}_{{\Lambda \left( {\widehat{{\varsigma_{ij} }}} \right)}} \left( {\mathfrak{u}} \right){ }} \right): {\mathfrak{u}} \in {U}} \right\}$, and ${\mathcal{T}}_{{\Lambda \left( {\widehat{{\varsigma_{ij} }}} \right)}} \left( {\mathfrak{u}} \right) = \left[ {{\mathcal{T}}_{{\Lambda_{{\widehat{{\varsigma_{ij} }}}} }}^{\ell } \left( {{\mathfrak{u}}_{i} } \right),{ }{\mathcal{T}}_{{\Lambda_{{\widehat{{\varsigma_{ij} }}}} }}^{\upsilon } \left( {{\mathfrak{u}}_{i} } \right)} \right]$ and ${\mathcal{J}}_{{\Lambda \left( {\widehat{{\varsigma_{ij} }}} \right)}} \left( {\mathfrak{u}} \right) = \left[ {{\mathcal{J}}_{{\Lambda_{{\widehat{{\varsigma_{ij} }}}} }}^{\ell } \left( {{\mathfrak{u}}_{i} } \right),{ }{\mathcal{J}}_{{\Lambda_{{\widehat{{\varsigma_{ij} }}}} }}^{\upsilon } \left( {{\mathfrak{u}}_{i} } \right)} \right]$ represents the MD and NMD, respectively, and ${\mathcal{T}}_{{\Lambda_{{\widehat{{\varsigma_{ij} }}}} }}^{\ell } \left( {{\mathfrak{u}}_{i} } \right),{ }{\mathcal{T}}_{{\Lambda_{{\widehat{{\varsigma_{ij} }}}} }}^{\upsilon } \left( {{\mathfrak{u}}_{i} } \right), {\mathcal{J}}_{{\Lambda_{{\widehat{{\varsigma_{ij} }}}} }}^{\ell } \left( {{\mathfrak{u}}_{i} } \right),{ }{\mathcal{J}}_{{\Lambda_{{\widehat{{\varsigma_{ij} }}}} }}^{\upsilon } \left( {{\mathfrak{u}}_{i} } \right) \in \left[ {0, 1} \right]$, such as $0 \le {\mathcal{T}}_{{\Lambda_{{\widehat{{\varsigma_{ij} }}}} }}^{\upsilon } \left( {{\mathfrak{u}}_{i} } \right) + {\mathcal{J}}_{{\Lambda_{{\widehat{{\varsigma_{ij} }}}} }}^{\upsilon } \left( {{\mathfrak{u}}_{i} } \right) \le 1$.

The IVIFHSN can be written as $\Lambda = \left\{ {\left( { \left[ {{\mathcal{T}}_{{\Lambda_{{\widehat{{\varsigma_{ij} }}}} }}^{\ell } \left( {{\mathfrak{u}}_{i} } \right),{ }{\mathcal{T}}_{{\Lambda_{{\widehat{{\varsigma_{ij} }}}} }}^{\upsilon } \left( {{\mathfrak{u}}_{i} } \right)} \right],{ }\left[ {{\mathcal{J}}_{{\Lambda_{{\widehat{{\varsigma_{ij} }}}} }}^{\ell } \left( {{\mathfrak{u}}_{i} } \right),{ }{\mathcal{J}}_{{\Lambda_{{\widehat{{\varsigma_{ij} }}}} }}^{\upsilon } \left( {{\mathfrak{u}}_{i} } \right)} \right]} \right)} \right\}$.

### Definition 6

^44^ Suppose we have a universe of discourse, denoted by ${U}$, which contains $n$ elements $\left\{ {{\mathfrak{u}}_{1},{\mathfrak{u}}_{2},{\mathfrak{u}}_{3},\ldots,{\mathfrak{u}}_{n} } \right\}$, $\left( {n \ge 1} \right)$. We also have a set of attributes, denoted by $\varsigma$, which contains $m$ attributes $\left\{ {\varsigma_{1} ,{ }\varsigma_{2} ,{ }\varsigma_{3} ,{ } \ldots ,{ }\varsigma_{m} } \right\}$ denotes the set of attributes and ${\mathfrak{T}}_{i}$ be the conforming sub-attributes, such as ${\mathfrak{T}}_{i} \cap {\mathfrak{T}}_{j} = {{\varphi }}$, where $i \ne j$ and $i, j \in \left\{ {1,2,3 \ldots n} \right\}$. Let ${\mathfrak{T}}_{1} \times {\mathfrak{T}}_{2} \times {\mathfrak{T}}_{3} \times \cdots \times {\mathfrak{T}}_{n} = {\mathop {\mathfrak{T}}\limits^{{...}}} = \left\{ {\left. {\left( {\varsigma_{{1j_{1} }},\varsigma_{{2j_{2} }},\ldots,\varsigma_{{nj_{n} }} } \right)} \right|\varsigma_{{1j_{1} }} \in {\mathfrak{T}}_{1},\varsigma_{{2j_{2} }} \in {\mathfrak{T}}_{2},\ldots,\varsigma_{{nj_{n} }} \in {\mathfrak{T}}_{n} } \right\}$ be a collection of multi-sub-attributes, where $1 \le j_{1} \le \alpha$, $1 \le j_{2} \le \beta$, and $1 \le j_{n} \le \gamma$, and $\alpha ,\beta ,\gamma \in {\text{N}}$. A pair $\left( {\Lambda ,{\mathfrak{T}}_{1} \times {\mathfrak{T}}_{2} \times {\mathfrak{T}}_{3} \times \cdots \times {\mathfrak{T}}_{n} = \left( {\Lambda, {\mathop {\mathfrak{T}}\limits^{{...}}} } \right)} \right)$ is entitled as PFHSS, and its mapping is defined as:

$$\Lambda :{\mathfrak{T}}_{1} \times {\mathfrak{T}}_{2} \times {\mathfrak{T}}_{3} \times \cdots \times {\mathfrak{T}}_{n} = {\mathop {\mathfrak{T}}\limits^{{...}}} \to PFHS^{U}$$.

Also, it can be defined as

$\left( {\Lambda, {\mathop {\mathfrak{T}}\limits^{{...}}} } \right) = \left\{ {\left( {\widehat{{\varsigma_{ij} }}, \Lambda_{{ {\mathop {\mathfrak{T}}\limits^{{...}}} }} \left( {\widehat{{\varsigma_{ij} }}} \right)} \right): \widehat{{\varsigma_{ij} }} \in {\mathop {\mathfrak{T}}\limits^{{...}}} , \Lambda_{{ {\mathop {\mathfrak{T}}\limits^{{...}}} }} \left( {\widehat{{\varsigma_{ij} }}} \right) \in PFS^{U} \in \left[ {0, 1} \right]} \right\}$, where $\Lambda _{{\mathop {\mathfrak{T}}\limits^{{...}}}} \left( {\widehat{{\varsigma _{{ij}} }}} \right) = \left\{ {\left\langle {{\mathfrak{u}},~{\mathcal{T}}_{{\Lambda \left( {\widehat{{\varsigma _{{ij}} }}} \right)}} \left( {\mathfrak{u}} \right),~{\mathcal{J}}_{{\Lambda \left( {\widehat{{\varsigma _{{ij}} }}} \right)}} \left( {\mathfrak{u}} \right)} \right\rangle :~{\mathfrak{u}} \in {U}} \right\}$, where ${\mathcal{T}}_{{\Lambda \left( {\widehat{{\varsigma_{ij} }}} \right)}} \left( {\mathfrak{u}} \right)$ and ${\mathcal{J}}_{{\Lambda \left( {\widehat{{\varsigma_{ij} }}} \right)}} \left( {\mathfrak{u}} \right)$ represents the MD and NMD, respectively, such as ${\mathcal{T}}_{{\Lambda \left( {\widehat{{\varsigma_{ij} }}} \right)}} \left( {\mathfrak{u}} \right)$, ${\mathcal{J}}_{{\Lambda \left( {\widehat{{\varsigma_{ij} }}} \right)}} \left( {\mathfrak{u}} \right) \in \left[ {0, 1} \right]$, and $0 \le \left( {{\mathcal{T}}_{{\Lambda \left( {\widehat{{\varsigma_{ij} }}} \right)}} \left( {\mathfrak{u}} \right)} \right)^{2} + \left( {{\mathcal{J}}_{{\Lambda \left( {\widehat{{\varsigma_{ij} }}} \right)}} \left( {\mathfrak{u}} \right)} \right)^{2} \le 1$.

The PFHSN can be specified as $\Lambda = \left\{ {\left( { {\mathcal{T}}_{{\Lambda \left( {\widehat{{\varsigma_{ij} }}} \right)}} \left( {\mathfrak{u}} \right),{ }{\mathcal{J}}_{{\Lambda \left( {\widehat{{\varsigma_{ij} }}} \right)}} \left( {\mathfrak{u}} \right){ }} \right)} \right\}$.

### Definition 7

^45^ Suppose we have a universe of discourse, denoted by ${U}$, which contains $n$ elements $\left\{ {{\mathfrak{u}}_{1},{\mathfrak{u}}_{2},{\mathfrak{u}}_{3},\ldots,{\mathfrak{u}}_{n} } \right\}$, $\left( {n \ge 1} \right)$. We also have a set of attributes, denoted by $\varsigma$, which contains $m$ attributes $\left\{ {\varsigma_{1} ,{ }\varsigma_{2} ,\varsigma_{3} ,{ } \ldots ,{ }\varsigma_{m} } \right\}$ indicates the set of parameters and ${\mathfrak{T}}_{i}$ be the conforming sub-parameters, such as ${\mathfrak{T}}_{i} \cap {\mathfrak{T}}_{j} = {{\varphi }}$, where $i \ne j$ and $i, j \in \left\{ {1,2,3 \ldots n} \right\}$. Let ${\mathfrak{T}}_{1} \times {\mathfrak{T}}_{2} \times {\mathfrak{T}}_{3} \times \cdots \times {\mathfrak{T}}_{n} = {\mathop {\mathfrak{T}}\limits^{{...}}} = \left\{ {\left. {\left( {\varsigma_{{1j_{1} }} ,\varsigma_{{2j_{2} }},\ldots,\varsigma_{{nj_{n} }} } \right)} \right|\varsigma_{{1j_{1} }} \in {\mathfrak{T}}_{1},\varsigma_{{2j_{2} }} \in {\mathfrak{T}}_{2},\ldots,\varsigma_{{nj_{n} }} \in {\mathfrak{T}}_{n} } \right\}$ be a collection of multi-sub-attributes, where $1 \le j_{1} \le \alpha$, $1 \le j_{2} \le \beta$, and $1 \le j_{n} \le \gamma$, and $\alpha ,\beta ,\gamma \in {\text{N}}$. A pair $\left( {\Lambda ,{\mathfrak{T}}_{1} \times {\mathfrak{T}}_{2} \times {\mathfrak{T}}_{3} \times \cdots \times {\mathfrak{T}}_{n} = \left( {\Lambda, {\mathop {\mathfrak{T}}\limits^{{...}}} } \right)} \right)$ is called an IVPFHSS, and its mapping is defined as:

$$\Lambda :{\mathfrak{T}}_{1} \times {\mathfrak{T}}_{2} \times {\mathfrak{T}}_{3} \times \cdots \times {\mathfrak{T}}_{n} = {\mathop {\mathfrak{T}}\limits^{{...}}} \to IVPFS^{U}$$.

Also, it can be defined as

$\left( {\Lambda, {\mathop {\mathfrak{T}}\limits^{{...}}} } \right) = \left\{ {\left( {\widehat{{\varsigma_{ij} }}, \Lambda_{{ {\mathop {\mathfrak{T}}\limits^{{...}}} }} \left( {\widehat{{\varsigma_{ij} }}} \right)} \right): \widehat{{\varsigma_{ij} }} \in {\mathop {\mathfrak{T}}\limits^{{...}}} , \Lambda_{{ {\mathop {\mathfrak{T}}\limits^{{...}}} }} \left( {\widehat{{\varsigma_{ij} }}} \right) \in IVPFS^{U} \in \left[ {0, 1} \right]} \right\}$, where $\Lambda _{{ {\mathop {\mathfrak{T}}\limits^{{...}}} }}\left( {\widehat{{\varsigma _{{ij}} }}} \right) = \left\{ {\left\langle {{\mathfrak{u}},~{\mathcal{T}}_{{\Lambda \left( {\widehat{{\varsigma _{{ij}} }}} \right)}} \left( {\mathfrak{u}} \right),~{\mathcal{J}}_{{\Lambda \left( {\widehat{{\varsigma _{{ij}} }}} \right)}} \left( {\mathfrak{u}} \right)} \right\rangle :~{\mathfrak{u}} \in {U}} \right\}$, and ${\mathcal{T}}_{{\Lambda \left( {\widehat{{\varsigma_{ij} }}} \right)}} \left( {\mathfrak{u}} \right) = \left[ {{\mathcal{T}}_{{\Lambda_{{\widehat{{\varsigma_{ij} }}}} }}^{\ell } \left( {{\mathfrak{u}}_{i} } \right),{ }{\mathcal{T}}_{{\Lambda_{{\widehat{{\varsigma_{ij} }}}} }}^{\upsilon } \left( {{\mathfrak{u}}_{i} } \right)} \right]$ and ${\mathcal{J}}_{{\Lambda \left( {\widehat{{\varsigma_{ij} }}} \right)}} \left( {\mathfrak{u}} \right) = \left[ {{\mathcal{J}}_{{\Lambda_{{\widehat{{\varsigma_{ij} }}}} }}^{\ell } \left( {{\mathfrak{u}}_{i} } \right),{ }{\mathcal{J}}_{{\Lambda_{{\widehat{{\varsigma_{ij} }}}} }}^{\upsilon } \left( {{\mathfrak{u}}_{i} } \right)} \right]$ represents the MD and NMD, respectively, and ${\mathcal{T}}_{{\Lambda_{{\widehat{{\varsigma_{ij} }}}} }}^{\ell } \left( {{\mathfrak{u}}_{i} } \right),{ }{\mathcal{T}}_{{\Lambda_{{\widehat{{\varsigma_{ij} }}}} }}^{\upsilon } \left( {{\mathfrak{u}}_{i} } \right), {\mathcal{J}}_{{\Lambda_{{\widehat{{\varsigma_{ij} }}}} }}^{\ell } \left( {{\mathfrak{u}}_{i} } \right),{ }{\mathcal{J}}_{{\Lambda_{{\widehat{{\varsigma_{ij} }}}} }}^{\upsilon } \left( {{\mathfrak{u}}_{i} } \right) \in \left[ {0, 1} \right]$, such as $0 \le \left( {{\mathcal{T}}_{{\Lambda_{{\widehat{{\varsigma_{ij} }}}} }}^{\upsilon } \left( {{\mathfrak{u}}_{i} } \right)} \right)^{2} + \left( {{\mathcal{J}}_{{\Lambda_{{\widehat{{\varsigma_{ij} }}}} }}^{\upsilon } \left( {{\mathfrak{u}}_{i} } \right)} \right)^{2} \le 1$.

The IVPFHSN can be written as $\Lambda = \left\{ {\left( { \left[ {{\mathcal{T}}_{{\Lambda_{{\widehat{{\varsigma_{ij} }}}} }}^{\ell } \left( {{\mathfrak{u}}_{i} } \right),{ }{\mathcal{T}}_{{\Lambda_{{\widehat{{\varsigma_{ij} }}}} }}^{\upsilon } \left( {{\mathfrak{u}}_{i} } \right)} \right],{ }\left[ {{\mathcal{J}}_{{\Lambda_{{\widehat{{\varsigma_{ij} }}}} }}^{\ell } \left( {{\mathfrak{u}}_{i} } \right),{ }{\mathcal{J}}_{{\Lambda_{{\widehat{{\varsigma_{ij} }}}} }}^{\upsilon } \left( {{\mathfrak{u}}_{i} } \right)} \right]} \right)} \right\}$.

Zulqarnain et al.^45^ introduced a set of algebraic operational laws for IVPFHSS. They used these operational laws to introduce a set of AOs for IVPFHSS.

$$\begin{gathered} {\text{IVPFHSWA}}\left( {\Lambda_{{\widehat{{\varsigma_{11} }}}} ,\Lambda_{{\widehat{{\varsigma_{12} }}}},\ldots \ldots \ldots ,\Lambda_{{\widehat{{\varsigma_{nm} }}}} } \right) \hfill \\ = \left( {\sqrt {1 - \mathop \prod \limits_{j = 1}^{m} \left( {\mathop \prod \limits_{i = 1}^{n} \left( {1 - \left[ {{\mathcal{T}}_{{\Lambda_{{\widehat{{\varsigma_{ij} }}}} }}^{\ell } \left( {{\mathfrak{u}}_{i} } \right),{ }{\mathcal{T}}_{{\Lambda_{{\widehat{{\varsigma_{ij} }}}} }}^{\upsilon } \left( {{\mathfrak{u}}_{i} } \right)} \right]^{2} } \right)^{{\Omega _{i} }} } \right)^{{{\upgamma }_{j} }} } ,\mathop \prod \limits_{j = 1}^{m} \left( {\mathop \prod \limits_{i = 1}^{n} \left( {\left[ {{\mathcal{J}}_{{\Lambda_{{\widehat{{\varsigma_{ij} }}}} }}^{\ell } \left( {{\mathfrak{u}}_{i} } \right),{ }{\mathcal{J}}_{{\Lambda_{{\widehat{{\varsigma_{ij} }}}} }}^{\upsilon } \left( {{\mathfrak{u}}_{i} } \right)} \right]} \right)^{{\Omega _{i} }} } \right)^{{{\upgamma }_{j} }} } \right) \hfill \\ \end{gathered}$$

$$\begin{gathered} {\text{IVPFHSWG}}\left( {\Lambda_{{\widehat{{\varsigma_{11} }}}} ,\Lambda_{{\widehat{{\varsigma_{12} }}}},\ldots \ldots \ldots ,\Lambda_{{\widehat{{\varsigma_{nm} }}}} } \right) \hfill \\ = \left( {\mathop \prod \limits_{j = 1}^{m} \left( {\mathop \prod \limits_{i = 1}^{n} \left( {\left[ {{\mathcal{T}}_{{\Lambda_{{\widehat{{\varsigma_{ij} }}}} }}^{\ell } \left( {{\mathfrak{u}}_{i} } \right),{ }{\mathcal{T}}_{{\Lambda_{{\widehat{{\varsigma_{ij} }}}} }}^{\upsilon } \left( {{\mathfrak{u}}_{i} } \right)} \right]} \right)^{{\Omega _{i} }} } \right)^{{{\upgamma }_{j} }} ,\sqrt {1 - \mathop \prod \limits_{j = 1}^{m} \left( {\mathop \prod \limits_{i = 1}^{n} \left( {1 - \left[ {{\mathcal{J}}_{{\Lambda_{{\widehat{{\varsigma_{ij} }}}} }}^{\ell } \left( {{\mathfrak{u}}_{i} } \right),{ }{\mathcal{J}}_{{\Lambda_{{\widehat{{\varsigma_{ij} }}}} }}^{\upsilon } \left( {{\mathfrak{u}}_{i} } \right)} \right]^{2} } \right)^{{\Omega _{i} }} } \right)^{{{\upgamma }_{j} }} } } \right) \hfill \\ \end{gathered}$$

The weight vectors $\Omega _{i}$ and ${\upgamma }_{j}$ are used in a DM process where experts evaluate attributes of alternatives. The vector $\Omega _{i}$ represents the weights given by the experts, where $i = 1,2,...,n$ and $n$ is the total number of experts, such as $\Omega _{i} > 0, \mathop \sum \nolimits_{i = 1}^{n}\Omega _{i} = 1$. Similarly, the vector ${\upgamma }_{j}$ represents the weights given by the experts, where $j = 1,2,...,m$ and $m$ is the total number of attributes, such as ${\upgamma }_{j} > 0, \mathop \sum \nolimits_{j = 1}^{m} {\upgamma }_{j} = 1$. The above-presented AOs for IVPFHSS cannot compute the most appropriate alternative using the closeness coefficient. To overcome these drawbacks, we are going to introduce correlation coefficients for IVPFHSS.

## Correlation coefficient for interval valued pythagorean fuzzy hypersoft set

In the consequent section, we present the CC for IVPFHSS with their essential properties.

### Definition 8

Let $\left( {\Lambda ,{\mathcal{A}}} \right) = \left\{ {\left. {\left( {{\mathfrak{u}}_{i},\left( {\left[ {{\mathcal{T}}_{{\Lambda_{{\widehat{{\varsigma_{k} }}}} }}^{\ell } \left( {{\mathfrak{u}}_{i} } \right),{ }{\mathcal{T}}_{{\Lambda_{{\widehat{{\varsigma_{k} }}}} }}^{\upsilon } \left( {{\mathfrak{u}}_{i} } \right)} \right], \left[ {{\mathcal{J}}_{{\Lambda_{{\widehat{{\varsigma_{k} }}}} }}^{\ell } \left( {{\mathfrak{u}}_{i} } \right),{ }{\mathcal{J}}_{{\Lambda_{{\widehat{{\varsigma_{k} }}}} }}^{\upsilon } \left( {{\mathfrak{u}}_{i} } \right)} \right]} \right)} \right)} \right| {\mathfrak{u}}_{i} \in {U}} \right\}$ and $\left( {{\mathcal{G}},{ \mathcal{B}}} \right) = \left\{ {\left. {\left( {{\mathfrak{u}}_{i},\left( {\left[ {{\mathcal{T}}_{{{\mathcal{G}}_{{\widehat{{\varsigma_{k} }}}} }}^{\ell } \left( {{\mathfrak{u}}_{i} } \right),{ }{\mathcal{T}}_{{{\mathcal{G}}_{{\widehat{{\varsigma_{k} }}}} }}^{\upsilon } \left( {{\mathfrak{u}}_{i} } \right)} \right], \left[ {{\mathcal{J}}_{{{\mathcal{G}}_{{\widehat{{\varsigma_{k} }}}} }}^{\ell } \left( {{\mathfrak{u}}_{i} } \right),{ }{\mathcal{J}}_{{{\mathcal{G}}_{{\widehat{{\varsigma_{k} }}}} }}^{\upsilon } \left( {{\mathfrak{u}}_{i} } \right)} \right]} \right)} \right) } \right|{\mathfrak{u}}_{i} \in {U}} \right\}$ be two IVPFHSS. Then, the informational energies between $\left( {\Lambda ,{\mathcal{A}}} \right)$ and $\left( {{\mathcal{G}},{ \mathcal{B}}} \right)$ can be defined as:

1 $${\mathcal{E}}_{IVPFHSS} \left( {\Lambda ,{\mathcal{A}}} \right) = \mathop \sum \limits_{k = 1}^{m} \mathop \sum \limits_{i = 1}^{n} \left( {\left( {{\mathcal{T}}_{{\Lambda_{{\widehat{{\varsigma_{k} }}}} }}^{\ell } \left( {{\mathfrak{u}}_{i} } \right)} \right)^{4} + \left( {{\mathcal{T}}_{{\Lambda_{{\widehat{{\varsigma_{k} }}}} }}^{\upsilon } \left( {{\mathfrak{u}}_{i} } \right)} \right)^{4} + \left( {{\mathcal{J}}_{{\Lambda_{{\widehat{{\varsigma_{k} }}}} }}^{\ell } \left( {{\mathfrak{u}}_{i} } \right)} \right)^{4} + \left( {{\mathcal{J}}_{{\Lambda_{{\widehat{{\varsigma_{k} }}}} }}^{\upsilon } \left( {{\mathfrak{u}}_{i} } \right)} \right)^{4} } \right)$$

2 $${\mathcal{E}}_{IVPFHSS} \left( {{\mathcal{G}},{ \mathcal{B}}} \right) = \mathop \sum \limits_{k = 1}^{m} \mathop \sum \limits_{i = 1}^{n} \left( {\left( {{\mathcal{T}}_{{{\mathcal{G}}_{{\widehat{{\varsigma_{k} }}}} }}^{\ell } \left( {{\mathfrak{u}}_{i} } \right)} \right)^{4} + \left( {{\mathcal{T}}_{{{\mathcal{G}}_{{\widehat{{\varsigma_{k} }}}} }}^{\upsilon } \left( {{\mathfrak{u}}_{i} } \right)} \right)^{4} + \left( {{\mathcal{J}}_{{{\mathcal{G}}_{{\widehat{{\varsigma_{k} }}}} }}^{\ell } \left( {{\mathfrak{u}}_{i} } \right)} \right)^{4} + \left( {{\mathcal{J}}_{{{\mathcal{G}}_{{\widehat{{\varsigma_{k} }}}} }}^{\upsilon } \left( {{\mathfrak{u}}_{i} } \right)} \right)^{4} } \right) .$$

These informational energies measure the total amount of information contained in the IVPFHSS.

### Definition 9

Let $\left( {\Lambda ,{\mathcal{A}}} \right) = \left\{ {\left. {\left( {{\mathfrak{u}}_{i},\left( {\left[ {{\mathcal{T}}_{{\Lambda_{{\widehat{{\varsigma_{k} }}}} }}^{\ell } \left( {{\mathfrak{u}}_{i} } \right),{ }{\mathcal{T}}_{{\Lambda_{{\widehat{{\varsigma_{k} }}}} }}^{\upsilon } \left( {{\mathfrak{u}}_{i} } \right)} \right], \left[ {{\mathcal{J}}_{{\Lambda_{{\widehat{{\varsigma_{k} }}}} }}^{\ell } \left( {{\mathfrak{u}}_{i} } \right),{ }{\mathcal{J}}_{{\Lambda_{{\widehat{{\varsigma_{k} }}}} }}^{\upsilon } \left( {{\mathfrak{u}}_{i} } \right)} \right]} \right)} \right) } \right|{\mathfrak{u}}_{i} \in {U}} \right\}$ and $\left( {{\mathcal{G}},{ \mathcal{B}}} \right) = \left\{ {\left. {\left( {{\mathfrak{u}}_{i},\left( {\left[ {{\mathcal{T}}_{{{\mathcal{G}}_{{\widehat{{\varsigma_{k} }}}} }}^{\ell } \left( {{\mathfrak{u}}_{i} } \right),{ }{\mathcal{T}}_{{{\mathcal{G}}_{{\widehat{{\varsigma_{k} }}}} }}^{\upsilon } \left( {{\mathfrak{u}}_{i} } \right)} \right], \left[ {{\mathcal{J}}_{{{\mathcal{G}}_{{\widehat{{\varsigma_{k} }}}} }}^{\ell } \left( {{\mathfrak{u}}_{i} } \right),{ }{\mathcal{J}}_{{{\mathcal{G}}_{{\widehat{{\varsigma_{k} }}}} }}^{\upsilon } \left( {{\mathfrak{u}}_{i} } \right)} \right]} \right)} \right) } \right|{\mathfrak{u}}_{i} \in {U}} \right\}$ be two IVPFHSS. Then, the correlation between $\left( {\Lambda ,{\mathcal{A}}} \right)$ and $\left( {{\mathcal{G}},{ \mathcal{B}}} \right)$ can be defined as:

3 $${\mathcal{C}}_{IVPFHSS} \left( {\left( {\Lambda ,{\mathcal{A}}} \right),{ }\left( {{\mathcal{G}},{ \mathcal{B}}} \right)} \right) = \sum\limits_{k = 1}^{m} {\sum\limits_{i = 1}^{n} {\left( {\begin{array}{*{20}c} {\left( {{\mathcal{T}}_{{\Lambda_{{\widehat{{\varsigma_{k} }}}} }}^{\ell } \left( {{\mathfrak{u}}_{i} } \right)} \right)^{2} *\left( {{\mathcal{T}}_{{{\mathcal{G}}_{{\widehat{{\varsigma_{k} }}}} }}^{\ell } \left( {{\mathfrak{u}}_{i} } \right)} \right)^{2} + \left( {{\mathcal{T}}_{{\Lambda_{{\widehat{{\varsigma_{k} }}}} }}^{\upsilon } \left( {{\mathfrak{u}}_{i} } \right)} \right)^{2} *\left( {{\mathcal{T}}_{{{\mathcal{G}}_{{\widehat{{\varsigma_{k} }}}} }}^{\upsilon } \left( {{\mathfrak{u}}_{i} } \right)} \right)^{2} + } \\ {\left( {{\mathcal{J}}_{{\Lambda_{{\widehat{{\varsigma_{k} }}}} }}^{\ell } \left( {{\mathfrak{u}}_{i} } \right)} \right)^{2} *\left( {{\mathcal{J}}_{{{\mathcal{G}}_{{\widehat{{\varsigma_{k} }}}} }}^{\ell } \left( {{\mathfrak{u}}_{i} } \right)} \right)^{2} + \left( {{\mathcal{J}}_{{\Lambda_{{\widehat{{\varsigma_{k} }}}} }}^{\upsilon } \left( {{\mathfrak{u}}_{i} } \right)} \right)^{2} *\left( {{\mathcal{J}}_{{{\mathcal{G}}_{{\widehat{{\varsigma_{k} }}}} }}^{\upsilon } \left( {{\mathfrak{u}}_{i} } \right)} \right)^{2} } \\ \end{array} } \right)} }$$

### Proposition 10

Let $\left( {\Lambda ,{\mathcal{A}}} \right) = \left\{ {\left. {\left( {{\mathfrak{u}}_{i},\left( {\left[ {{\mathcal{T}}_{{\Lambda_{{\widehat{{\varsigma_{k} }}}} }}^{\ell } \left( {{\mathfrak{u}}_{i} } \right),{ }{\mathcal{T}}_{{\Lambda_{{\widehat{{\varsigma_{k} }}}} }}^{\upsilon } \left( {{\mathfrak{u}}_{i} } \right)} \right], \left[ {{\mathcal{J}}_{{\Lambda_{{\widehat{{\varsigma_{k} }}}} }}^{\ell } \left( {{\mathfrak{u}}_{i} } \right),{ }{\mathcal{J}}_{{\Lambda_{{\widehat{{\varsigma_{k} }}}} }}^{\upsilon } \left( {{\mathfrak{u}}_{i} } \right)} \right]} \right)} \right)} \right| {\mathfrak{u}}_{i} \in {U}} \right\}$ and $\left( {{\mathcal{G}},{ \mathcal{B}}} \right) = \left\{ {\left. {\left( {{\mathfrak{u}}_{i},\left( {\left[ {{\mathcal{T}}_{{{\mathcal{G}}_{{\widehat{{\varsigma_{k} }}}} }}^{\ell } \left( {{\mathfrak{u}}_{i} } \right),{ }{\mathcal{T}}_{{{\mathcal{G}}_{{\widehat{{\varsigma_{k} }}}} }}^{\upsilon } \left( {{\mathfrak{u}}_{i} } \right)} \right], \left[ {{\mathcal{J}}_{{{\mathcal{G}}_{{\widehat{{\varsigma_{k} }}}} }}^{\ell } \left( {{\mathfrak{u}}_{i} } \right),{ }{\mathcal{J}}_{{{\mathcal{G}}_{{\widehat{{\varsigma_{k} }}}} }}^{\upsilon } \left( {{\mathfrak{u}}_{i} } \right)} \right]} \right)} \right)} \right| {\mathfrak{u}}_{i} \in {U}} \right\}$ be two IVPFHSS. Then

1. $${\mathcal{C}}_{IVPFHSS} \left( {\left( {\Lambda ,{\mathcal{A}}} \right),{ }\left( {\Lambda ,{\mathcal{A}}} \right)} \right) = {\mathcal{E}}_{IVPFHSS} \left( {\Lambda ,{\mathcal{A}}} \right)$$
2. $${\mathcal{C}}_{IVPFHSS} \left( {\left( {\Lambda ,{\mathcal{A}}} \right),{ }\left( {{\mathcal{G}},{ \mathcal{B}}} \right)} \right) = {\mathcal{C}}_{IVPFHSS} \left( {\left( {{\mathcal{G}},{ \mathcal{B}}} \right), \left( {\Lambda ,{\mathcal{A}}} \right)} \right)$$.

### Proof 1:

As $\left( {\Lambda ,{\mathcal{A}}} \right) = \left\{ {\left. {\left( {{\mathfrak{u}}_{i},\left( {\left[ {{\mathcal{T}}_{{\Lambda_{{\widehat{{\varsigma_{k} }}}} }}^{\ell } \left( {{\mathfrak{u}}_{i} } \right),{ }{\mathcal{T}}_{{\Lambda_{{\widehat{{\varsigma_{k} }}}} }}^{\upsilon } \left( {{\mathfrak{u}}_{i} } \right)} \right], \left[ {{\mathcal{J}}_{{\Lambda_{{\widehat{{\varsigma_{k} }}}} }}^{\ell } \left( {{\mathfrak{u}}_{i} } \right),{ }{\mathcal{J}}_{{\Lambda_{{\widehat{{\varsigma_{k} }}}} }}^{\upsilon } \left( {{\mathfrak{u}}_{i} } \right)} \right]} \right)} \right)} \right| {\mathfrak{u}}_{i} \in {U}} \right\}$ be an IVPFHSS. Using Eq. (3), we get

$${\mathcal{C}}_{IVPFHSS}\left(\left(\Lambda ,\mathcal{A}\right), \left(\Lambda ,\mathcal{A}\right)\right)=\sum_{k=1}^{m}\sum_{i=1}^{n}\left(\begin{array}{c}{\left({\mathcal{T}}_{{\Lambda }_{\widehat{{ \varsigma }_{k}}}}^{\ell}\left({\mathfrak{u}}_{i}\right)\right)}^{2}*{\left({\mathcal{T}}_{{\Lambda }_{\widehat{{ \varsigma }_{k}}}}^{\ell}\left({\mathfrak{u}}_{i}\right)\right)}^{2}+{\left({\mathcal{T}}_{{\Lambda }_{\widehat{{ \varsigma }_{k}}}}^{\upsilon}\left({\mathfrak{u}}_{i}\right)\right)}^{2}*{\left({\mathcal{T}}_{{\Lambda }_{\widehat{{ \varsigma }_{k}}}}^{\upsilon}\left({\mathfrak{u}}_{i}\right)\right)}^{2}+\\ {\left({\mathcal{J}}_{{\Lambda }_{\widehat{{ \varsigma }_{k}}}}^{\ell}\left({\mathfrak{u}}_{i}\right)\right)}^{2}*{\left({\mathcal{J}}_{{\Lambda }_{\widehat{{ \varsigma }_{k}}}}^{\ell}\left({\mathfrak{u}}_{i}\right)\right)}^{2}+{\left({\mathcal{J}}_{{\Lambda }_{\widehat{{ \varsigma }_{k}}}}^{\upsilon}\left({\mathfrak{u}}_{i}\right)\right)}^{2}*{\left({\mathcal{J}}_{{\Lambda }_{\widehat{{ \varsigma }_{k}}}}^{\upsilon}\left({\mathfrak{u}}_{i}\right)\right)}^{2}\end{array}\right)$$

$${\mathcal{C}}_{IVPFHSS} \left( {\left( {\Lambda ,{\mathcal{A}}} \right),{ }\left( {\Lambda ,{\mathcal{A}}} \right)} \right) = \mathop \sum \limits_{k = 1}^{m} \mathop \sum \limits_{i = 1}^{n} \left( {\left( {{\mathcal{T}}_{{\Lambda_{{\widehat{{\varsigma_{k} }}}} }}^{\ell } \left( {{\mathfrak{u}}_{i} } \right)} \right)^{2} + \left( {{\mathcal{T}}_{{\Lambda_{{\widehat{{\varsigma_{k} }}}} }}^{\upsilon } \left( {{\mathfrak{u}}_{i} } \right)} \right)^{2} + \left( {{\mathcal{J}}_{{\Lambda_{{\widehat{{\varsigma_{k} }}}} }}^{\ell } \left( {{\mathfrak{u}}_{i} } \right)} \right)^{2} + \left( {{\mathcal{J}}_{{\Lambda_{{\widehat{{\varsigma_{k} }}}} }}^{\upsilon } \left( {{\mathfrak{u}}_{i} } \right)} \right)^{2} } \right)$$

$${\mathcal{C}}_{IVPFHSS} \left( {\left( {\Lambda ,{\mathcal{A}}} \right),{ }\left( {\Lambda ,{\mathcal{A}}} \right)} \right) = {\mathcal{E}}_{IVPFHSS} \left( {\Lambda ,{\mathcal{A}}} \right)$$.

### Proof 2:

Proof is very simple and straightforward.

### Definition 11

Let $\left( {\Lambda ,{\mathcal{A}}} \right) = \left\{ {\left. {\left( {{\mathfrak{u}}_{i},\left( {\left[ {{\mathcal{T}}_{{\Lambda_{{\widehat{{\varsigma_{k} }}}} }}^{\ell } \left( {{\mathfrak{u}}_{i} } \right),{ }{\mathcal{T}}_{{\Lambda_{{\widehat{{\varsigma_{k} }}}} }}^{\upsilon } \left( {{\mathfrak{u}}_{i} } \right)} \right], \left[ {{\mathcal{J}}_{{\Lambda_{{\widehat{{\varsigma_{k} }}}} }}^{\ell } \left( {{\mathfrak{u}}_{i} } \right),{ }{\mathcal{J}}_{{\Lambda_{{\widehat{{\varsigma_{k} }}}} }}^{\upsilon } \left( {{\mathfrak{u}}_{i} } \right)} \right]} \right)} \right)} \right| {\mathfrak{u}}_{i} \in {U}} \right\}$ and $\left( {{\mathcal{G}},{ \mathcal{B}}} \right) = \left\{ {\left. {\left( {{\mathfrak{u}}_{i},\left( {\left[ {{\mathcal{T}}_{{{\mathcal{G}}_{{\widehat{{\varsigma_{k} }}}} }}^{\ell } \left( {{\mathfrak{u}}_{i} } \right),{ }{\mathcal{T}}_{{{\mathcal{G}}_{{\widehat{{\varsigma_{k} }}}} }}^{\upsilon } \left( {{\mathfrak{u}}_{i} } \right)} \right], \left[ {{\mathcal{J}}_{{{\mathcal{G}}_{{\widehat{{\varsigma_{k} }}}} }}^{\ell } \left( {{\mathfrak{u}}_{i} } \right),{ }{\mathcal{J}}_{{{\mathcal{G}}_{{\widehat{{\varsigma_{k} }}}} }}^{\upsilon } \left( {{\mathfrak{u}}_{i} } \right)} \right]} \right)} \right) } \right|{\mathfrak{u}}_{i} \in {U}} \right\}$ be two IVPFHSS. Then, CC between them is defined as:

4 $$\begin{aligned} & {\mathbb{C}}_{{IVPFHSS}} \left( {\left( {\Lambda ,{\mathcal{A}}} \right),{}\left( {{\mathcal{G}},{~\mathcal{B}}} \right)} \right) = \frac{{{\mathcal{C}}_{{IVPFHSS}} \left( {\left( {\Lambda ,{\mathcal{A}}} \right),{}\left( {{\mathcal{G}},{~\mathcal{B}}} \right)} \right)}}{{\sqrt {{\mathcal{E}}_{{IVPFHSS}} \left( {\Lambda ,{\mathcal{A}}} \right)} \sqrt {{\mathcal{E}}_{{IVPFHSS}} \left( {{\mathcal{G}},{~\mathcal{B}}} \right)} }} \hfill \\ & \quad= \frac{{\mathop \sum \limits_{{k = 1}}^{m} \mathop \sum \limits_{{i = 1}}^{n} \left( {\begin{aligned} & {\left( {{\mathcal{T}}_{{\Lambda _{{\widehat{{\varsigma _{k} }}}} }}^{\ell } \left( {{\mathfrak{u}}_{i} } \right)} \right)^{2} *\left( {{\mathcal{T}}_{{{\mathcal{G}}_{{\widehat{{\varsigma _{k} }}}} }}^{\ell } \left( {{\mathfrak{u}}_{i} } \right)} \right)^{2} + \left( {{\mathcal{T}}_{{\Lambda _{{\widehat{{\varsigma _{k} }}}} }}^{\upsilon } \left( {{\mathfrak{u}}_{i} } \right)} \right)^{2} *\left( {{\mathcal{T}}_{{{\mathcal{G}}_{{\widehat{{\varsigma _{k} }}}} }}^{\upsilon } \left( {{\mathfrak{u}}_{i} } \right)} \right)^{2} }\\ & \quad {+ \left( {{\mathcal{J}}_{{\Lambda _{{\widehat{{\varsigma _{k} }}}} }}^{\ell } \left( {{\mathfrak{u}}_{i} } \right)} \right)^{2} *\left( {{\mathcal{J}}_{{{\mathcal{G}}_{{\widehat{{\varsigma _{k} }}}} }}^{\ell } \left( {{\mathfrak{u}}_{i} } \right)} \right)^{2} + \left( {{\mathcal{J}}_{{\Lambda _{{\widehat{{\varsigma _{k} }}}} }}^{\upsilon } \left( {{\mathfrak{u}}_{i} } \right)} \right)^{2} *\left( {{\mathcal{J}}_{{{\mathcal{G}}_{{\widehat{{\varsigma _{k} }}}} }}^{\upsilon } \left( {{\mathfrak{u}}_{i} } \right)} \right)^{2} } \\ \end{aligned} } \right)}}{\begin{aligned}& {\sqrt {\mathop \sum \limits_{{k = 1}}^{m} \mathop \sum \limits_{{i = 1}}^{n} \left( {\left( {{\mathcal{T}}_{{\Lambda _{{\widehat{{\varsigma _{k} }}}} }}^{\ell } \left( {{\mathfrak{u}}_{i} } \right)} \right)^{4} + \left( {{\mathcal{T}}_{{\Lambda _{{\widehat{{\varsigma _{k} }}}} }}^{\upsilon } \left( {{\mathfrak{u}}_{i} } \right)} \right)^{4} + \left( {{\mathcal{J}}_{{\Lambda _{{\widehat{{\varsigma _{k} }}}} }}^{\ell } \left( {{\mathfrak{u}}_{i} } \right)} \right)^{4} + \left( {{\mathcal{J}}_{{\Lambda _{{\widehat{{\varsigma _{k} }}}} }}^{\upsilon } \left( {{\mathfrak{u}}_{i} } \right)} \right)^{4} } \right)}}\\ & \quad{ \sqrt {\mathop \sum \limits_{{k = 1}}^{m} \mathop \sum \limits_{{i = 1}}^{n} \left( {\left( {{\mathcal{T}}_{{{\mathcal{G}}_{{\widehat{{\varsigma _{k} }}}} }}^{\ell } \left( {{\mathfrak{u}}_{i} } \right)} \right)^{4} + \left( {{\mathcal{T}}_{{{\mathcal{G}}_{{\widehat{{\varsigma _{k} }}}} }}^{\upsilon } \left( {{\mathfrak{u}}_{i} } \right)} \right)^{4} + \left( {{\mathcal{J}}_{{{\mathcal{G}}_{{\widehat{{\varsigma _{k} }}}} }}^{\ell } \left( {{\mathfrak{u}}_{i} } \right)} \right)^{4} + \left( {{\mathcal{J}}_{{{\mathcal{G}}_{{\widehat{{\varsigma _{k} }}}} }}^{\upsilon } \left( {{\mathfrak{u}}_{i} } \right)} \right)^{4} } \right)} }\end{aligned} } \hfill \\ \end{aligned}$$

### Theorem 12

Let $\left( {\Lambda ,{\mathcal{A}}} \right) = \left\{ {\left. {\left( {{\mathfrak{u}}_{i},\left( {\left[ {{\mathcal{T}}_{{\Lambda_{{\widehat{{\varsigma_{k} }}}} }}^{\ell } \left( {{\mathfrak{u}}_{i} } \right),{ }{\mathcal{T}}_{{\Lambda_{{\widehat{{\varsigma_{k} }}}} }}^{\upsilon } \left( {{\mathfrak{u}}_{i} } \right)} \right], \left[ {{\mathcal{J}}_{{\Lambda_{{\widehat{{\varsigma_{k} }}}} }}^{\ell } \left( {{\mathfrak{u}}_{i} } \right),{ }{\mathcal{J}}_{{\Lambda_{{\widehat{{\varsigma_{k} }}}} }}^{\upsilon } \left( {{\mathfrak{u}}_{i} } \right)} \right]} \right)} \right) } \right|{\mathfrak{u}}_{i} \in {U}} \right\}$ and $\left( {{\mathcal{G}},{ \mathcal{B}}} \right) = \left\{ {\left. {\left( {{\mathfrak{u}}_{i},\left( {\left[ {{\mathcal{T}}_{{{\mathcal{G}}_{{\widehat{{\varsigma_{k} }}}} }}^{\ell } \left( {{\mathfrak{u}}_{i} } \right),{ }{\mathcal{T}}_{{{\mathcal{G}}_{{\widehat{{\varsigma_{k} }}}} }}^{\upsilon } \left( {{\mathfrak{u}}_{i} } \right)} \right], \left[ {{\mathcal{J}}_{{{\mathcal{G}}_{{\widehat{{\varsigma_{k} }}}} }}^{\ell } \left( {{\mathfrak{u}}_{i} } \right),{ }{\mathcal{J}}_{{{\mathcal{G}}_{{\widehat{{\varsigma_{k} }}}} }}^{\upsilon } \left( {{\mathfrak{u}}_{i} } \right)} \right]} \right)} \right) } \right|{\mathfrak{u}}_{i} \in {U}} \right\}$ be two IVPFHSS. Then, the following possessions are held:

1. $$0 \le {\mathbb{C}}_{IVPFHSS} \left( {\left( {\Lambda ,{\mathcal{A}}} \right),\left( {{\mathcal{G}},{ \mathcal{B}}} \right)} \right) \le 1$$
2. $${\mathbb{C}}_{IVPFHSS} \left( {\left( {\Lambda ,{\mathcal{A}}} \right),\left( {{\mathcal{G}},{ \mathcal{B}}} \right)} \right) = {\mathbb{C}}_{IVPFHSS} \left( {\left( {{\mathcal{G}},{ \mathcal{B}}} \right),\left( {\Lambda ,{\mathcal{A}}} \right)} \right)$$
3. If $\left( {\Lambda ,{\mathcal{A}}} \right) = \left( {{\mathcal{G}},{ \mathcal{B}}} \right)$, i.e., I$\forall$ $i$, $j$, ${\mathcal{T}}_{{\Lambda_{{\widehat{{\varsigma_{k} }}}} }}^{\ell } \left( {{\mathfrak{u}}_{i} } \right) = {\mathcal{T}}_{{{\mathcal{G}}_{{\widehat{{\varsigma_{k} }}}} }}^{\ell } \left( {{\mathfrak{u}}_{i} } \right)$, ${\mathcal{T}}_{{\Lambda_{{\widehat{{\varsigma_{k} }}}} }}^{\upsilon } \left( {{\mathfrak{u}}_{i} } \right) = {\mathcal{T}}_{{{\mathcal{G}}_{{\widehat{{\varsigma_{k} }}}} }}^{\upsilon } \left( {{\mathfrak{u}}_{i} } \right)$, ${\mathcal{J}}_{{\Lambda_{{\widehat{{\varsigma_{k} }}}} }}^{\ell } \left( {{\mathfrak{u}}_{i} } \right) = {\mathcal{J}}_{{{\mathcal{G}}_{{\widehat{{\varsigma_{k} }}}} }}^{\ell } \left( {{\mathfrak{u}}_{i} } \right)$, and ${\mathcal{J}}_{{\Lambda_{{\widehat{{\varsigma_{k} }}}} }}^{\upsilon } \left( {{\mathfrak{u}}_{i} } \right) = {\mathcal{J}}_{{{\mathcal{G}}_{{\widehat{{\varsigma_{k} }}}} }}^{\upsilon } \left( {{\mathfrak{u}}_{i} } \right)$, then ${\mathbb{C}}_{IVPFHSS} \left( {\left( {\Lambda ,{\mathcal{A}}} \right),{ }\left( {{\mathcal{G}},{ \mathcal{B}}} \right)} \right) =$ 1.

### Proof 1.

${\mathbb{C}}_{IVPFHSS} \left( {\left( {\Lambda ,{\mathcal{A}}} \right),\left( {{\mathcal{G}},{ \mathcal{B}}} \right)} \right) \ge 0$ is obvious. Now, weIwill demonstrate ${\mathbb{C}}_{IVPFHSS} \left( {\left( {\Lambda ,{\mathcal{A}}} \right),\left( {{\mathcal{G}},{ \mathcal{B}}} \right)} \right) \le 1$. Employing Eq. (3).

$${\mathcal{C}}_{IVPFHSS} \left( {\left( {\Lambda ,{\mathcal{A}}} \right),{ }\left( {{\mathcal{G}},{ \mathcal{B}}} \right)} \right) =$$

$$\begin{aligned}& \mathop \sum \limits_{k = 1}^{m} \mathop \sum \limits_{i = 1}^{n} \left( {{\left( {{\mathcal{T}}_{{\Lambda_{{\widehat{{\varsigma_{k} }}}} }}^{\ell } \left( {{\mathfrak{u}}_{i} } \right)} \right)^{2} *\left( {{\mathcal{T}}_{{{\mathcal{G}}_{{\widehat{{\varsigma_{k} }}}} }}^{\ell } \left( {{\mathfrak{u}}_{i} } \right)} \right)^{2} + \left( {{\mathcal{T}}_{{\Lambda_{{\widehat{{\varsigma_{k} }}}} }}^{\upsilon } \left( {{\mathfrak{u}}_{i} } \right)} \right)^{2} *\left( {{\mathcal{T}}_{{{\mathcal{G}}_{{\widehat{{\varsigma_{k} }}}} }}^{\upsilon } \left( {{\mathfrak{u}}_{i} } \right)} \right)^{2}}}\right. \\ & \quad + \left.{ \left( {{\mathcal{J}}_{{\Lambda_{{\widehat{{\varsigma_{k} }}}} }}^{\ell } \left( {{\mathfrak{u}}_{i} } \right)} \right)^{2} {*}\left( {{\mathcal{J}}_{{{\mathcal{G}}_{{\widehat{{\varsigma_{k} }}}} }}^{\ell } \left( {{\mathfrak{u}}_{i} } \right)} \right)^{2} + \left( {{\mathcal{J}}_{{\Lambda_{{\widehat{{\varsigma_{k} }}}} }}^{\upsilon } \left( {{\mathfrak{u}}_{i} } \right)} \right)^{2} *\left( {{\mathcal{J}}_{{{\mathcal{G}}_{{\widehat{{\varsigma_{k} }}}} }}^{\upsilon } \left( {{\mathfrak{u}}_{i} } \right)} \right)^{2} } \right) \end{aligned}$$

$$\begin{aligned} & = \mathop \sum \limits_{k = 1}^{m} \left( { \left( {{\mathcal{T}}_{{\Lambda_{{\widehat{{\varsigma_{k} }}}} }}^{\ell } \left( {{\mathfrak{u}}_{1} } \right)} \right)^{2} *\left( {{\mathcal{T}}_{{{\mathcal{G}}_{{\widehat{{\varsigma_{k} }}}} }}^{\ell } \left( {{\mathfrak{u}}_{1} } \right)} \right)^{2} + \left( {{\mathcal{T}}_{{\Lambda_{{\widehat{{\varsigma_{k} }}}} }}^{\upsilon } \left( {{\mathfrak{u}}_{1} } \right)} \right)^{2} *\left( {{\mathcal{T}}_{{{\mathcal{G}}_{{\widehat{{\varsigma_{k} }}}} }}^{\upsilon } \left( {{\mathfrak{u}}_{1} } \right)} \right)^{2}} \right. \\ & \quad + \left. { \left( {{\mathcal{J}}_{{\Lambda_{{\widehat{{\varsigma_{k} }}}} }}^{\ell } \left( {{\mathfrak{u}}_{1} } \right)} \right)^{2} {*}\left( {{\mathcal{J}}_{{{\mathcal{G}}_{{\widehat{{\varsigma_{k} }}}} }}^{\ell } \left( {{\mathfrak{u}}_{1} } \right)} \right)^{2} + \left( {{\mathcal{J}}_{{\Lambda_{{\widehat{{\varsigma_{k} }}}} }}^{\upsilon } \left( {{\mathfrak{u}}_{1} } \right)} \right)^{2} *\left( {{\mathcal{J}}_{{{\mathcal{G}}_{{\widehat{{\varsigma_{k} }}}} }}^{\upsilon } \left( {{\mathfrak{u}}_{1} } \right)} \right)^{2} } \right)\end{aligned}$$

$$\begin{aligned} & + \mathop \sum \limits_{{k = 1}}^{m} \left( {\left( {{\mathcal{T}}_{{\Lambda _{{\widehat{{\varsigma _{k} }}}} }}^{\ell } \left( {{\mathfrak{u}}_{2} } \right)} \right)^{2} *\left( {{\mathcal{T}}_{{{\mathcal{G}}f_{{\widehat{{\varsigma _{k} }}}} }}^{\ell } \left( {{\mathfrak{u}}_{2} } \right)} \right)^{2} + \left( {{\mathcal{T}}_{{\Lambda _{{\widehat{{\varsigma _{k} }}}} }}^{\upsilon } \left( {{\mathfrak{u}}_{2} } \right)} \right)^{2} *\left( {{\mathcal{T}}_{{{\mathcal{G}}_{{\widehat{{\varsigma _{k} }}}} }}^{\upsilon } \left( {{\mathfrak{u}}_{2} } \right)} \right)^{2} } \right. \\ & \quad + \left. { \left( {{\mathcal{J}}_{{\Lambda _{{\widehat{{\varsigma _{k} }}}} }}^{\ell } \left( {{\mathfrak{u}}_{2} } \right)} \right)^{2} {\text{*}}\left( {{\mathcal{J}}_{{{\mathcal{G}}_{{\widehat{{\varsigma _{k} }}}} }}^{\ell } \left( {{\mathfrak{u}}_{2} } \right)} \right)^{2} + \left( {{\mathcal{J}}_{{\Lambda _{{\widehat{{\varsigma _{k} }}}} }}^{\upsilon } \left( {{\mathfrak{u}}_{2} } \right)} \right)^{2} *\left( {{\mathcal{J}}_{{{\mathcal{G}}_{{\widehat{{\varsigma _{k} }}}} }}^{\upsilon } \left( {{\mathfrak{u}}_{2} } \right)} \right)^{2} } \right) \end{aligned}$$.

$$+$$

$$\vdots$$

$$+$$

$$\begin{aligned} & \mathop \sum \limits_{k = 1}^{m} \left( { \left( {{\mathcal{T}}_{{\Lambda_{{\widehat{{\varsigma_{k} }}}} }}^{\ell } \left( {{\mathfrak{u}}_{n} } \right)} \right)^{2} *\left( {{\mathcal{T}}_{{{\mathcal{G}}_{{\widehat{{\varsigma_{k} }}}} }}^{\ell } \left( {{\mathfrak{u}}_{n} } \right)} \right)^{2} + \left( {{\mathcal{T}}_{{\Lambda_{{\widehat{{\varsigma_{k} }}}} }}^{\upsilon } \left( {{\mathfrak{u}}_{n} } \right)} \right)^{2} *\left( {{\mathcal{T}}_{{{\mathcal{G}}_{{\widehat{{\varsigma_{k} }}}} }}^{\upsilon } \left( {{\mathfrak{u}}_{n} } \right)} \right)^{2} }\right.\\ & \quad\left.{+ \left( {{\mathcal{J}}_{{\Lambda_{{\widehat{{\varsigma_{k} }}}} }}^{\ell } \left( {{\mathfrak{u}}_{n} } \right)} \right)^{2} {*}\left( {{\mathcal{J}}_{{{\mathcal{G}}_{{\widehat{{\varsigma_{k} }}}} }}^{\ell } \left( {{\mathfrak{u}}_{n} } \right)} \right)^{2} + \left( {{\mathcal{J}}_{{\Lambda_{{\widehat{{\varsigma_{k} }}}} }}^{\upsilon } \left( {{\mathfrak{u}}_{n} } \right)} \right)^{2} *\left( {{\mathcal{J}}_{{{\mathcal{G}}_{{\widehat{{\varsigma_{k} }}}} }}^{\upsilon } \left( {{\mathfrak{u}}_{n} } \right)} \right)^{2} } \right) \end{aligned}$$

$$\begin{aligned} & {\mathcal{C}}_{IVPFHSS}\left(\left(\Lambda ,\mathcal{A}\right), \left(\mathcal{G},\mathcal{B}\right)\right)\\ &\quad=\left\{\begin{array}{c}\left(\begin{array}{l}{\left({\mathcal{T}}_{{\Lambda }_{\widehat{{ \varsigma }_{1}}}}^{\ell}\left({\mathfrak{u}}_{1}\right)\right)}^{2}*{\left({\mathcal{T}}_{{\mathcal{G}}_{\widehat{{ \varsigma }_{1}}}}^{\ell}\left({\mathfrak{u}}_{1}\right)\right)}^{2}+{\left({\mathcal{T}}_{{\Lambda }_{\widehat{{ \varsigma }_{1}}}}^{\upsilon}\left({\mathfrak{u}}_{1}\right)\right)}^{2}*{\left({\mathcal{T}}_{{\mathcal{G}}_{\widehat{{ \varsigma }_{1}}}}^{\upsilon}\left({\mathfrak{u}}_{1}\right)\right)}^{2}\\\quad +{\left({\mathcal{J}}_{{\Lambda }_{\widehat{{ \varsigma }_{1}}}}^{\ell}\left({\mathfrak{u}}_{1}\right)\right)}^{2}*{\left({\mathcal{J}}_{{\mathcal{G}}_{\widehat{{ \varsigma }_{1}}}}^{\ell}\left({\mathfrak{u}}_{1}\right)\right)}^{2}+{\left({\mathcal{J}}_{{\Lambda }_{\widehat{{ \varsigma }_{1}}}}^{\upsilon}\left({\mathfrak{u}}_{1}\right)\right)}^{2}*{\left({\mathcal{J}}_{{\mathcal{G}}_{\widehat{{ \varsigma }_{1}}}}^{\upsilon}\left({\mathfrak{u}}_{1}\right)\right)}^{2}\end{array}\right)+\\ \ \left(\begin{array}{l}{\left({\mathcal{T}}_{{\Lambda }_{\widehat{{ \varsigma }_{2}}}}^{\ell}\left({\mathfrak{u}}_{1}\right)\right)}^{2}*{\left({\mathcal{T}}_{{\mathcal{G}}_{\widehat{{ \varsigma }_{2}}}}^{\ell}\left({\mathfrak{u}}_{1}\right)\right)}^{2}+{\left({\mathcal{T}}_{{\Lambda }_{\widehat{{ \varsigma }_{2}}}}^{\upsilon}\left({\mathfrak{u}}_{1}\right)\right)}^{2}*{\left({\mathcal{T}}_{{\mathcal{G}}_{\widehat{{ \varsigma }_{2}}}}^{\upsilon}\left({\mathfrak{u}}_{1}\right)\right)}^{2}\\ \quad +{\left({\mathcal{J}}_{{\Lambda }_{\widehat{{ \varsigma }_{2}}}}^{\ell}\left({\mathfrak{u}}_{1}\right)\right)}^{2}*{\left({\mathcal{J}}_{{\mathcal{G}}_{\widehat{{ \varsigma }_{2}}}}^{\ell}\left({\mathfrak{u}}_{1}\right)\right)}^{2}+{\left({\mathcal{J}}_{{\Lambda }_{\widehat{{ \varsigma }_{2}}}}^{\upsilon}\left({\mathfrak{u}}_{1}\right)\right)}^{2}*{\left({\mathcal{J}}_{{\mathcal{G}}_{\widehat{{ \varsigma }_{2}}}}^{\upsilon}\left({\mathfrak{u}}_{1}\right)\right)}^{2}\end{array}\right)+\\ \vdots \\ +\\ \left(\begin{array}{l}{\left({\mathcal{T}}_{{\Lambda }_{\widehat{{ \varsigma }_{m}}}}^{\ell}\left({\mathfrak{u}}_{1}\right)\right)}^{2}*{\left({\mathcal{T}}_{{\mathcal{G}}_{\widehat{{ \varsigma }_{m}}}}^{\ell}\left({\mathfrak{u}}_{1}\right)\right)}^{2}+{\left({\mathcal{T}}_{{\Lambda }_{\widehat{{ \varsigma }_{m}}}}^{\upsilon}\left({\mathfrak{u}}_{1}\right)\right)}^{2}*{\left({\mathcal{T}}_{{\mathcal{G}}_{\widehat{{ \varsigma }_{m}}}}^{\upsilon}\left({\mathfrak{u}}_{1}\right)\right)}^{2}\\\quad +{\left({\mathcal{J}}_{{\Lambda }_{\widehat{{ \varsigma }_{m}}}}^{\ell}\left({\mathfrak{u}}_{1}\right)\right)}^{2}*{\left({\mathcal{J}}_{{\mathcal{G}}_{\widehat{{ \varsigma }_{m}}}}^{\ell}\left({\mathfrak{u}}_{1}\right)\right)}^{2}+{\left({\mathcal{J}}_{{\Lambda }_{\widehat{{ \varsigma }_{m}}}}^{\upsilon}\left({\mathfrak{u}}_{1}\right)\right)}^{2}*{\left({\mathcal{J}}_{{\mathcal{G}}_{\widehat{{ \varsigma }_{m}}}}^{\upsilon}\left({\mathfrak{u}}_{1}\right)\right)}^{2}\end{array}\right)\end{array}\right\}\end{aligned}$$

$$+$$

$$\left\{\begin{array}{c}\left(\begin{array}{l}{\left({\mathcal{T}}_{{\Lambda }_{\widehat{{{{ \varsigma }}}_{1}}}}^{\ell}\left({\mathfrak{u}}_{2}\right)\right)}^{2}*{\left({\mathcal{T}}_{{\mathcal{G}}_{\widehat{{{{ \varsigma }}}_{1}}}}^{\ell}\left({\mathfrak{u}}_{2}\right)\right)}^{2}+{\left({\mathcal{T}}_{{\Lambda }_{\widehat{{{{ \varsigma }}}_{1}}}}^{{{\upsilon}}}\left({\mathfrak{u}}_{2}\right)\right)}^{2}*{\left({\mathcal{T}}_{{\mathcal{G}}_{\widehat{{{{ \varsigma }}}_{1}}}}^{{{\upsilon}}}\left({\mathfrak{u}}_{2}\right)\right)}^{2}\\ \quad +{\left({\mathcal{J}}_{{\Lambda }_{\widehat{{{{ \varsigma }}}_{1}}}}^{\ell}\left({\mathfrak{u}}_{2}\right)\right)}^{2}*{\left({\mathcal{J}}_{{\mathcal{G}}_{\widehat{{{{ \varsigma }}}_{1}}}}^{\ell}\left({\mathfrak{u}}_{2}\right)\right)}^{2}+{\left({\mathcal{J}}_{{\Lambda }_{\widehat{{{{ \varsigma }}}_{1}}}}^{{{\upsilon}}}\left({\mathfrak{u}}_{2}\right)\right)}^{2}*{\left({\mathcal{J}}_{{\mathcal{G}}_{\widehat{{{{ \varsigma }}}_{1}}}}^{{{\upsilon}}}\left({\mathfrak{u}}_{2}\right)\right)}^{2}\end{array}\right)+\\ \left(\begin{array}{l}{\left({\mathcal{T}}_{{\Lambda }_{\widehat{{{{ \varsigma }}}_{2}}}}^{\ell}\left({\mathfrak{u}}_{2}\right)\right)}^{2}*{\left({\mathcal{T}}_{{\mathcal{G}}_{\widehat{{{{ \varsigma }}}_{2}}}}^{\ell}\left({\mathfrak{u}}_{2}\right)\right)}^{2}+{\left({\mathcal{T}}_{{\Lambda }_{\widehat{{{{ \varsigma }}}_{2}}}}^{{{\upsilon}}}\left({\mathfrak{u}}_{2}\right)\right)}^{2}*{\left({\mathcal{T}}_{{\mathcal{G}}_{\widehat{{{{ \varsigma }}}_{2}}}}^{{{\upsilon}}}\left({\mathfrak{u}}_{2}\right)\right)}^{2}\\ \quad +{\left({\mathcal{J}}_{{\Lambda }_{\widehat{{{{ \varsigma }}}_{2}}}}^{\ell}\left({\mathfrak{u}}_{2}\right)\right)}^{2}*{\left({\mathcal{J}}_{{\mathcal{G}}_{\widehat{{{{ \varsigma }}}_{2}}}}^{\ell}\left({\mathfrak{u}}_{2}\right)\right)}^{2}+{\left({\mathcal{J}}_{{\Lambda }_{\widehat{{{{ \varsigma }}}_{2}}}}^{{{\upsilon}}}\left({\mathfrak{u}}_{2}\right)\right)}^{2}*{\left({\mathcal{J}}_{{\mathcal{G}}_{\widehat{{{{ \varsigma }}}_{2}}}}^{{{\upsilon}}}\left({\mathfrak{u}}_{2}\right)\right)}^{2}\end{array}\right)+\\ \vdots \\ +\\ \left(\begin{array}{l}{\left({\mathcal{T}}_{{\Lambda }_{\widehat{{{{ \varsigma }}}_{m}}}}^{\ell}\left({\mathfrak{u}}_{2}\right)\right)}^{2}*{\left({\mathcal{T}}_{{\mathcal{G}}_{\widehat{{{{ \varsigma }}}_{m}}}}^{\ell}\left({\mathfrak{u}}_{2}\right)\right)}^{2}+{\left({\mathcal{T}}_{{\Lambda }_{\widehat{{{{ \varsigma }}}_{m}}}}^{{{\upsilon}}}\left({\mathfrak{u}}_{2}\right)\right)}^{2}*{\left({\mathcal{T}}_{{\mathcal{G}}_{\widehat{{{{ \varsigma }}}_{m}}}}^{{{\upsilon}}}\left({\mathfrak{u}}_{2}\right)\right)}^{2}\\ \quad +{\left({\mathcal{J}}_{{\Lambda }_{\widehat{{{{ \varsigma }}}_{m}}}}^{\ell}\left({\mathfrak{u}}_{2}\right)\right)}^{2}*{\left({\mathcal{J}}_{{\mathcal{G}}_{\widehat{{{{ \varsigma }}}_{m}}}}^{\ell}\left({\mathfrak{u}}_{2}\right)\right)}^{2}+{\left({\mathcal{J}}_{{\Lambda }_{\widehat{{{{ \varsigma }}}_{m}}}}^{{{\upsilon}}}\left({\mathfrak{u}}_{2}\right)\right)}^{2}*{\left({\mathcal{J}}_{{\mathcal{G}}_{\widehat{{{{ \varsigma }}}_{m}}}}^{{{\upsilon}}}\left({\mathfrak{u}}_{2}\right)\right)}^{2}\end{array}\right)\end{array}\right\}$$

$$+$$

$$\vdots$$

$$+$$

$$\left\{\begin{array}{c}\left(\begin{array}{l}{\left({\mathcal{T}}_{{\Lambda }_{\widehat{{ \varsigma }_{1}}}}^{\ell}\left({\mathfrak{u}}_{n}\right)\right)}^{2}*{\left({\mathcal{T}}_{{\mathcal{G}}_{\widehat{{ \varsigma }_{1}}}}^{\ell}\left({\mathfrak{u}}_{n}\right)\right)}^{2}+{\left({\mathcal{T}}_{{\Lambda }_{\widehat{{ \varsigma }_{1}}}}^{\upsilon}\left({\mathfrak{u}}_{n}\right)\right)}^{2}*{\left({\mathcal{T}}_{{\mathcal{G}}_{\widehat{{ \varsigma }_{1}}}}^{\upsilon}\left({\mathfrak{u}}_{n}\right)\right)}^{2}\\\quad +{\left({\mathcal{J}}_{{\Lambda }_{\widehat{{ \varsigma }_{1}}}}^{\ell}\left({\mathfrak{u}}_{n}\right)\right)}^{2}*{\left({\mathcal{J}}_{{\mathcal{G}}_{\widehat{{ \varsigma }_{1}}}}^{\ell}\left({\mathfrak{u}}_{n}\right)\right)}^{2}+{\left({\mathcal{J}}_{{\Lambda }_{\widehat{{ \varsigma }_{1}}}}^{\upsilon}\left({\mathfrak{u}}_{n}\right)\right)}^{2}*{\left({\mathcal{J}}_{{\mathcal{G}}_{\widehat{{ \varsigma }_{1}}}}^{\upsilon}\left({\mathfrak{u}}_{n}\right)\right)}^{2}\end{array}\right)+\\ \left(\begin{array}{l}{\left({\mathcal{T}}_{{\Lambda }_{\widehat{{ \varsigma }_{2}}}}^{\ell}\left({\mathfrak{u}}_{n}\right)\right)}^{2}*{\left({\mathcal{T}}_{{\mathcal{G}}_{\widehat{{ \varsigma }_{2}}}}^{\ell}\left({\mathfrak{u}}_{n}\right)\right)}^{2}+{\left({\mathcal{T}}_{{\Lambda }_{\widehat{{ \varsigma }_{2}}}}^{\upsilon}\left({\mathfrak{u}}_{n}\right)\right)}^{2}*{\left({\mathcal{T}}_{{\mathcal{G}}_{\widehat{{ \varsigma }_{2}}}}^{\upsilon}\left({\mathfrak{u}}_{n}\right)\right)}^{2}\\ \quad +{\left({\mathcal{J}}_{{\Lambda }_{\widehat{{ \varsigma }_{2}}}}^{\ell}\left({\mathfrak{u}}_{n}\right)\right)}^{2}*{\left({\mathcal{J}}_{{\mathcal{G}}_{\widehat{{ \varsigma }_{2}}}}^{\ell}\left({\mathfrak{u}}_{n}\right)\right)}^{2}+{\left({\mathcal{J}}_{{\Lambda }_{\widehat{{ \varsigma }_{2}}}}^{\upsilon}\left({\mathfrak{u}}_{n}\right)\right)}^{2}*{\left({\mathcal{J}}_{{\mathcal{G}}_{\widehat{{ \varsigma }_{2}}}}^{\upsilon}\left({\mathfrak{u}}_{n}\right)\right)}^{2}\end{array}\right)+\\ \vdots \\ +\\ \left(\begin{array}{l}{\left({\mathcal{T}}_{{\Lambda }_{\widehat{{ \varsigma }_{m}}}}^{\ell}\left({\mathfrak{u}}_{n}\right)\right)}^{2}*{\left({\mathcal{T}}_{{\mathcal{G}}_{\widehat{{ \varsigma }_{m}}}}^{\ell}\left({\mathfrak{u}}_{n}\right)\right)}^{2}+{\left({\mathcal{T}}_{{\Lambda }_{\widehat{{ \varsigma }_{m}}}}^{\upsilon}\left({\mathfrak{u}}_{n}\right)\right)}^{2}*{\left({\mathcal{T}}_{{\mathcal{G}}_{\widehat{{ \varsigma }_{m}}}}^{\upsilon}\left({\mathfrak{u}}_{n}\right)\right)}^{2}\\ \quad +{\left({\mathcal{J}}_{{\Lambda }_{\widehat{{ \varsigma }_{m}}}}^{\ell}\left({\mathfrak{u}}_{n}\right)\right)}^{2}*{\left({\mathcal{J}}_{{\mathcal{G}}_{\widehat{{ \varsigma }_{m}}}}^{\ell}\left({\mathfrak{u}}_{n}\right)\right)}^{2}+{\left({\mathcal{J}}_{{\Lambda }_{\widehat{{ \varsigma }_{m}}}}^{\upsilon}\left({\mathfrak{u}}_{n}\right)\right)}^{2}*{\left({\mathcal{J}}_{{\mathcal{G}}_{\widehat{{ \varsigma }_{m}}}}^{\upsilon}\left({\mathfrak{u}}_{n}\right)\right)}^{2}\end{array}\right)\end{array}\right\}$$

$$+\sum_{k=1}^{m}\left(\begin{array}{l}\left({\left({\mathcal{J}}_{{\Lambda }_{\widehat{{ \varsigma }_{k}}}}^{\ell}\left({\mathfrak{u}}_{1}\right)\right)}^{2}*{\left({\mathcal{J}}_{{\mathcal{G}}_{\widehat{{ \varsigma }_{k}}}}^{\ell}\left({\mathfrak{u}}_{1}\right)\right)}^{2}+{\left({\mathcal{J}}_{{\Lambda }_{\widehat{{ \varsigma }_{k}}}}^{\upsilon}\left({\mathfrak{u}}_{1}\right)\right)}^{2}*{\left({\mathcal{J}}_{{\mathcal{G}}_{\widehat{{ \varsigma }_{k}}}}^{\upsilon}\left({\mathfrak{u}}_{1}\right)\right)}^{2}\right)\\ \quad+\left({\left({\mathcal{J}}_{{\Lambda }_{\widehat{{ \varsigma }_{k}}}}^{\ell}\left({\mathfrak{u}}_{2}\right)\right)}^{2}*{\left({\mathcal{J}}_{{\mathcal{G}}_{\widehat{{ \varsigma }_{k}}}}^{\ell}\left({\mathfrak{u}}_{2}\right)\right)}^{2}+{\left({\mathcal{J}}_{{\Lambda }_{\widehat{{ \varsigma }_{k}}}}^{\upsilon}\left({\mathfrak{u}}_{2}\right)\right)}^{2}*{\left({\mathcal{J}}_{{\mathcal{G}}_{\widehat{{ \varsigma }_{k}}}}^{\upsilon}\left({\mathfrak{u}}_{2}\right)\right)}^{2}\right)\\\quad +\cdots \cdots +\left({\left({\mathcal{J}}_{{\Lambda }_{\widehat{{ \varsigma }_{k}}}}^{\ell}\left({\mathfrak{u}}_{n}\right)\right)}^{2}*{\left({\mathcal{J}}_{{\mathcal{G}}_{\widehat{{ \varsigma }_{k}}}}^{\ell}\left({\mathfrak{u}}_{n}\right)\right)}^{2}+{\left({\mathcal{J}}_{{\Lambda }_{\widehat{{ \varsigma }_{k}}}}^{\upsilon}\left({\mathfrak{u}}_{n}\right)\right)}^{2}*{\left({\mathcal{J}}_{{\mathcal{G}}_{\widehat{{ \varsigma }_{k}}}}^{\upsilon}\left({\mathfrak{u}}_{n}\right)\right)}^{2}\right)\end{array}\right)$$

$$+\sum_{k=1}^{m}\left(\begin{array}{c}\left({\left({\mathcal{J}}_{{\Lambda }_{\widehat{{ \varsigma }_{k}}}}^{\ell}\left({\mathfrak{u}}_{1}\right)\right)}^{2}*{\left({\mathcal{J}}_{{\mathcal{G}}_{\widehat{{ \varsigma }_{k}}}}^{\ell}\left({\mathfrak{u}}_{1}\right)\right)}^{2}+{\left({\mathcal{J}}_{{\Lambda }_{\widehat{{ \varsigma }_{k}}}}^{\upsilon}\left({\mathfrak{u}}_{1}\right)\right)}^{2}*{\left({\mathcal{J}}_{{\mathcal{G}}_{\widehat{{ \varsigma }_{k}}}}^{\upsilon}\left({\mathfrak{u}}_{1}\right)\right)}^{2}\right)+\left({\left({\mathcal{J}}_{{\Lambda }_{\widehat{{ \varsigma }_{k}}}}^{\ell}\left({\mathfrak{u}}_{2}\right)\right)}^{2}*{\left({\mathcal{J}}_{{\mathcal{G}}_{\widehat{{ \varsigma }_{k}}}}^{\ell}\left({\mathfrak{u}}_{2}\right)\right)}^{2}+{\left({\mathcal{J}}_{{\Lambda }_{\widehat{{ \varsigma }_{k}}}}^{\upsilon}\left({\mathfrak{u}}_{2}\right)\right)}^{2}*{\left({\mathcal{J}}_{{\mathcal{G}}_{\widehat{{ \varsigma }_{k}}}}^{\upsilon}\left({\mathfrak{u}}_{2}\right)\right)}^{2}\right)\\ +\cdots \cdots +\left({\left({\mathcal{J}}_{{\Lambda }_{\widehat{{ \varsigma }_{k}}}}^{\ell}\left({\mathfrak{u}}_{n}\right)\right)}^{2}*{\left({\mathcal{J}}_{{\mathcal{G}}_{\widehat{{ \varsigma }_{k}}}}^{\ell}\left({\mathfrak{u}}_{n}\right)\right)}^{2}+{\left({\mathcal{J}}_{{\Lambda }_{\widehat{{ \varsigma }_{k}}}}^{\upsilon}\left({\mathfrak{u}}_{n}\right)\right)}^{2}*{\left({\mathcal{J}}_{{\mathcal{G}}_{\widehat{{ \varsigma }_{k}}}}^{\upsilon}\left({\mathfrak{u}}_{n}\right)\right)}^{2}\right)\end{array}\right)$$

Employing the Cauchy Schwarz inequality

$$\begin{aligned} & {{\mathcal{C}}_{IVPFHSS}\left(\left(\Lambda ,\mathcal{A}\right),\left(\mathcal{G},\mathcal{B}\right)\right)}^{2}\\ &\quad\le \sum_{k=1}^{m}\left\{\begin{array}{l}\left({\left({\mathcal{T}}_{{\Lambda }_{\widehat{{ \varsigma }_{k}}}}^{\ell}\left({\mathfrak{u}}_{1}\right)\right)}^{4}+{\left({\mathcal{T}}_{{\Lambda }_{\widehat{{ \varsigma }_{k}}}}^{\upsilon}\left({\mathfrak{u}}_{1}\right)\right)}^{4}\right)+\left({\left({\mathcal{T}}_{{\Lambda }_{\widehat{{ \varsigma }_{k}}}}^{\ell}\left({\mathfrak{u}}_{2}\right)\right)}^{4}+{\left({\mathcal{T}}_{{\Lambda }_{\widehat{{ \varsigma }_{k}}}}^{\upsilon}\left({\mathfrak{u}}_{2}\right)\right)}^{4}\right)+\\ \quad \dots +\left({\left({\mathcal{T}}_{{\Lambda }_{\widehat{{ \varsigma }_{k}}}}^{\ell}\left({\mathfrak{u}}_{n}\right)\right)}^{4}+{\left({\mathcal{T}}_{{\Lambda }_{\widehat{{ \varsigma }_{k}}}}^{\upsilon}\left({\mathfrak{u}}_{n}\right)\right)}^{4}\right)+\\ \left({\left({\mathcal{J}}_{{\Lambda }_{\widehat{{ \varsigma }_{k}}}}^{\ell}\left({\mathfrak{u}}_{1}\right)\right)}^{4}+{\left({\mathcal{J}}_{{\Lambda }_{\widehat{{ \varsigma }_{k}}}}^{\upsilon}\left({\mathfrak{u}}_{1}\right)\right)}^{4}\right)+\left({\left({\mathcal{J}}_{{\Lambda }_{\widehat{{ \varsigma }_{k}}}}^{\ell}\left({\mathfrak{u}}_{2}\right)\right)}^{4}+{\left({\mathcal{J}}_{{\Lambda }_{\widehat{{ \varsigma }_{k}}}}^{\upsilon}\left({\mathfrak{u}}_{2}\right)\right)}^{4}\right)+\\ \quad \dots +\left({\left({\mathcal{J}}_{{\Lambda }_{\widehat{{ \varsigma }_{k}}}}^{\ell}\left({\mathfrak{u}}_{n}\right)\right)}^{4}+{\left({\mathcal{J}}_{{\Lambda }_{\widehat{{ \varsigma }_{k}}}}^{\upsilon}\left({\mathfrak{u}}_{n}\right)\right)}^{4}\right)\end{array}\right\}\\ &\quad\quad\times \sum_{k=1}^{m}\left\{\begin{array}{l}\left({\left({\mathcal{T}}_{{\mathcal{G}}_{\widehat{{ \varsigma }_{k}}}}^{\ell}\left({\mathfrak{u}}_{1}\right)\right)}^{4}+{\left({\mathcal{T}}_{{\mathcal{G}}_{\widehat{{ \varsigma }_{k}}}}^{\upsilon}\left({\mathfrak{u}}_{1}\right)\right)}^{4}\right)+\left({\left({\mathcal{T}}_{{\mathcal{G}}_{\widehat{{ \varsigma }_{k}}}}^{\ell}\left({\mathfrak{u}}_{2}\right)\right)}^{4}+{\left({\mathcal{T}}_{{\mathcal{G}}_{\widehat{{ \varsigma }_{k}}}}^{\upsilon}\left({\mathfrak{u}}_{2}\right)\right)}^{4}\right)+\\ \quad \dots +\left({\left({\mathcal{T}}_{{\mathcal{G}}_{\widehat{{ \varsigma }_{k}}}}^{\ell}\left({\mathfrak{u}}_{n}\right)\right)}^{4}+{\left({\mathcal{T}}_{{\mathcal{G}}_{\widehat{{ \varsigma }_{k}}}}^{\upsilon}\left({\mathfrak{u}}_{n}\right)\right)}^{4}\right)+\\ \left({\left({\mathcal{J}}_{{\mathcal{G}}_{\widehat{{ \varsigma }_{k}}}}^{\ell}\left({\mathfrak{u}}_{1}\right)\right)}^{4}+{\left({\mathcal{J}}_{{\mathcal{G}}_{\widehat{{ \varsigma }_{k}}}}^{\upsilon}\left({\mathfrak{u}}_{1}\right)\right)}^{4}\right)+\left({\left({\mathcal{J}}_{{\mathcal{G}}_{\widehat{{ \varsigma }_{k}}}}^{\ell}\left({\mathfrak{u}}_{2}\right)\right)}^{4}+{\left({\mathcal{J}}_{{\mathcal{G}}_{\widehat{{ \varsigma }_{k}}}}^{\upsilon}\left({\mathfrak{u}}_{2}\right)\right)}^{4}\right)+\\ \quad \dots +\left({\left({\mathcal{J}}_{{\mathcal{G}}_{\widehat{{ \varsigma }_{k}}}}^{\ell}\left({\mathfrak{u}}_{n}\right)\right)}^{4}+{\left({\mathcal{J}}_{{\mathcal{G}}_{\widehat{{ \varsigma }_{k}}}}^{\upsilon}\left({\mathfrak{u}}_{n}\right)\right)}^{4}\right)\end{array}\right\}\end{aligned}$$

$$\begin{aligned} & {{\mathcal{C}}_{IVPFHSS}\left(\left(\Lambda ,\mathcal{A}\right),\left(\mathcal{G},\mathcal{B}\right)\right)}^{2}\\ &\quad\le \sum_{k=1}^{m}\sum_{i=1}^{n}\left\{\left({\left({\mathcal{T}}_{{\Lambda }_{\widehat{{ \varsigma }_{k}}}}^{\ell}\left({\mathfrak{u}}_{i}\right)\right)}^{4}+{\left({\mathcal{T}}_{{\Lambda }_{\widehat{{ \varsigma }_{k}}}}^{\upsilon}\left({\mathfrak{u}}_{i}\right)\right)}^{4}\right)+\left({\left({\mathcal{J}}_{{\Lambda }_{\widehat{{ \varsigma }_{k}}}}^{\ell}\left({\mathfrak{u}}_{i}\right)\right)}^{4}+{\left({\mathcal{J}}_{{\Lambda }_{\widehat{{ \varsigma }_{k}}}}^{\upsilon}\left({\mathfrak{u}}_{i}\right)\right)}^{4}\right)\right\}\\ &\quad\quad\times \sum_{k=1}^{m}\sum_{i=1}^{n}\left\{\left({\left({\mathcal{T}}_{{\mathcal{G}}_{\widehat{{ \varsigma }_{k}}}}^{\ell}\left({\mathfrak{u}}_{i}\right)\right)}^{4}+{\left({\mathcal{T}}_{{\mathcal{G}}_{\widehat{{ \varsigma }_{k}}}}^{\upsilon}\left({\mathfrak{u}}_{i}\right)\right)}^{4}\right)+\left({\left({\mathcal{J}}_{{\mathcal{G}}_{\widehat{{ \varsigma }_{k}}}}^{\ell}\left({\mathfrak{u}}_{i}\right)\right)}^{4}+{\left({\mathcal{J}}_{{\mathcal{G}}_{\widehat{{ \varsigma }_{k}}}}^{\upsilon}\left({\mathfrak{u}}_{i}\right)\right)}^{4}\right)\right\}\end{aligned}$$

$${{\mathcal{C}}_{IVPFHSS}\left(\left(\Lambda ,\mathcal{A}\right), \left(\mathcal{G},\mathcal{B}\right)\right)}^{2}\le {\mathcal{E}}_{IVPFHSS}\left(\Lambda ,\mathcal{A}\right)\times {\mathcal{E}}_{IVPFHSS}\left(\mathcal{G},\mathcal{B}\right)$$

Using Definition 11, we get .

$${\mathbb{C}}_{IVPFHSS}\left(\left(\Lambda ,\mathcal{A}\right), \left(\mathcal{G},\mathcal{B}\right)\right)\le 1.$$

So, it is verified that $0\le {\mathbb{C}}_{IVPFHSS}\left(\left(\Lambda ,\mathcal{A}\right), \left(\mathcal{G},\mathcal{B}\right)\right)\le 1.$

### Proof 2.

As $\left(\Lambda ,\mathcal{A}\right)=\left\{\left({\mathfrak{u}}_{i}, \left(\left[{\mathcal{T}}_{{\Lambda }_{\widehat{{ \varsigma }_{k}}}}^{\ell}\left({\mathfrak{u}}_{i}\right), {\mathcal{T}}_{{\Lambda }_{\widehat{{ \varsigma }_{k}}}}^{\upsilon}\left({\mathfrak{u}}_{i}\right)\right], \left[{\mathcal{J}}_{{\Lambda }_{\widehat{{ \varsigma }_{k}}}}^{\ell}\left({\mathfrak{u}}_{i}\right), {\mathcal{J}}_{{\Lambda }_{\widehat{{ \varsigma }_{k}}}}^{\upsilon}\left({\mathfrak{u}}_{i}\right)\right]\right)\right) \left. {} \right| {\mathfrak{u}}_{i}\in \mathsf{U}\right\}$ and $\left(\mathcal{G},\mathcal{B}\right)=\left\{\left({\mathfrak{u}}_{i}, \left(\left[{\mathcal{T}}_{{\mathcal{G}}_{\widehat{{ \varsigma }_{k}}}}^{\ell}\left({\mathfrak{u}}_{i}\right), {\mathcal{T}}_{{\mathcal{G}}_{\widehat{{ \varsigma }_{k}}}}^{\upsilon}\left({\mathfrak{u}}_{i}\right)\right], \left[{\mathcal{J}}_{{\mathcal{G}}_{\widehat{{ \varsigma }_{k}}}}^{\ell}\left({\mathfrak{u}}_{i}\right), {\mathcal{J}}_{{\mathcal{G}}_{\widehat{{ \varsigma }_{k}}}}^{\upsilon}\left({\mathfrak{u}}_{i}\right)\right]\right)\right) \left. {} \right| {\mathfrak{u}}_{i}\in \mathsf{U}\right\}$ be two IVPFHSS. From Eq. (4)

$$\begin{aligned}& {\mathbb{C}}_{IVPFHSS}\left(\left(\Lambda ,\mathcal{A}\right), \left(\mathcal{G},\mathcal{B}\right)\right)\\ & \quad=\frac{\sum_{k=1}^{m}\sum_{i=1}^{n}\left(\begin{array}{l}{\left({\mathcal{T}}_{{\Lambda }_{\widehat{{ \varsigma }_{k}}}}^{\ell}\left({\mathfrak{u}}_{i}\right)\right)}^{2}*{\left({\mathcal{T}}_{{\mathcal{G}}_{\widehat{{ \varsigma }_{k}}}}^{\ell}\left({\mathfrak{u}}_{i}\right)\right)}^{2}+{\left({\mathcal{T}}_{{\Lambda }_{\widehat{{ \varsigma }_{k}}}}^{\upsilon}\left({\mathfrak{u}}_{i}\right)\right)}^{2}*{\left({\mathcal{T}}_{{\mathcal{G}}_{\widehat{{ \varsigma }_{k}}}}^{\upsilon}\left({\mathfrak{u}}_{i}\right)\right)}^{2}\\ \quad +{\left({\mathcal{J}}_{{\Lambda }_{\widehat{{ \varsigma }_{k}}}}^{\ell}\left({\mathfrak{u}}_{i}\right)\right)}^{2}*{\left({\mathcal{J}}_{{\mathcal{G}}_{\widehat{{ \varsigma }_{k}}}}^{\ell}\left({\mathfrak{u}}_{i}\right)\right)}^{2}+{\left({\mathcal{J}}_{{\Lambda }_{\widehat{{ \varsigma }_{k}}}}^{\upsilon}\left({\mathfrak{u}}_{i}\right)\right)}^{2}*{\left({\mathcal{J}}_{{\mathcal{G}}_{\widehat{{ \varsigma }_{k}}}}^{\upsilon}\left({\mathfrak{u}}_{i}\right)\right)}^{2}\end{array}\right)}{\begin{array}{l}\sqrt{\sum_{k=1}^{m}\sum_{i=1}^{n}\left({\left({\mathcal{T}}_{{\Lambda }_{\widehat{{ \varsigma }_{k}}}}^{\ell}\left({\mathfrak{u}}_{i}\right)\right)}^{4}+{\left({\mathcal{T}}_{{\Lambda }_{\widehat{{ \varsigma }_{k}}}}^{\upsilon}\left({\mathfrak{u}}_{i}\right)\right)}^{4}+{\left({\mathcal{J}}_{{\Lambda }_{\widehat{{ \varsigma }_{k}}}}^{\ell}\left({\mathfrak{u}}_{i}\right)\right)}^{4}+{\left({\mathcal{J}}_{{\Lambda }_{\widehat{{ \varsigma }_{k}}}}^{\upsilon}\left({\mathfrak{u}}_{i}\right)\right)}^{4}\right)}\\ \quad \sqrt{\sum_{k=1}^{m}\sum_{i=1}^{n}\left({\left({\mathcal{T}}_{{\mathcal{G}}_{\widehat{{ \varsigma }_{k}}}}^{\ell}\left({\mathfrak{u}}_{i}\right)\right)}^{4}+{\left({\mathcal{T}}_{{\mathcal{G}}_{\widehat{{ \varsigma }_{k}}}}^{\upsilon}\left({\mathfrak{u}}_{i}\right)\right)}^{4}+{\left({\mathcal{J}}_{{\mathcal{G}}_{\widehat{{ \varsigma }_{k}}}}^{\ell}\left({\mathfrak{u}}_{i}\right)\right)}^{4}+{\left({\mathcal{J}}_{{\mathcal{G}}_{\widehat{{ \varsigma }_{k}}}}^{\upsilon}\left({\mathfrak{u}}_{i}\right)\right)}^{4}\right)}\end{array}}\end{aligned}$$

$$\begin{aligned}=\frac{\sum_{k=1}^{m}\sum_{i=1}^{n}\left(\begin{array}{l}{\left({\mathcal{T}}_{{\mathcal{G}}_{\widehat{{ \varsigma }_{k}}}}^{\ell}\left({\mathfrak{u}}_{i}\right)\right)}^{2}*{\left({\mathcal{T}}_{{\Lambda }_{\widehat{{ \varsigma }_{k}}}}^{\ell}\left({\mathfrak{u}}_{i}\right)\right)}^{2}+{\left({\mathcal{T}}_{{\mathcal{G}}_{\widehat{{ \varsigma }_{k}}}}^{\upsilon}\left({\mathfrak{u}}_{i}\right)\right)}^{2}*{\left({\mathcal{T}}_{{\Lambda }_{\widehat{{ \varsigma }_{k}}}}^{\upsilon}\left({\mathfrak{u}}_{i}\right)\right)}^{2}\\ \quad+{\left({\mathcal{J}}_{{\mathcal{G}}_{\widehat{{ \varsigma }_{k}}}}^{\ell}\left({\mathfrak{u}}_{i}\right)\right)}^{2}*{\left({\mathcal{J}}_{{\Lambda }_{\widehat{{ \varsigma }_{k}}}}^{\ell}\left({\mathfrak{u}}_{i}\right)\right)}^{2}+{\left({\mathcal{J}}_{{\mathcal{G}}_{\widehat{{ \varsigma }_{k}}}}^{\upsilon}\left({\mathfrak{u}}_{i}\right)\right)}^{2}*{\left({\mathcal{J}}_{{\Lambda }_{\widehat{{ \varsigma }_{k}}}}^{\upsilon}\left({\mathfrak{u}}_{i}\right)\right)}^{2}\end{array}\right)}{\begin{array}{l}\sqrt{\sum_{k=1}^{m}\sum_{i=1}^{n}\left({\left({\mathcal{T}}_{{\mathcal{G}}_{\widehat{{ \varsigma }_{k}}}}^{\ell}\left({\mathfrak{u}}_{i}\right)\right)}^{4}+{\left({\mathcal{T}}_{{\mathcal{G}}_{\widehat{{ \varsigma }_{k}}}}^{\upsilon}\left({\mathfrak{u}}_{i}\right)\right)}^{4}+{\left({\mathcal{J}}_{{\mathcal{G}}_{\widehat{{ \varsigma }_{k}}}}^{\ell}\left({\mathfrak{u}}_{i}\right)\right)}^{4}+{\left({\mathcal{J}}_{{\mathcal{G}}_{\widehat{{ \varsigma }_{k}}}}^{\upsilon}\left({\mathfrak{u}}_{i}\right)\right)}^{4}\right)}\\ \sqrt{\sum_{k=1}^{m}\sum_{i=1}^{n}\left({\left({\mathcal{T}}_{{\Lambda }_{\widehat{{ \varsigma }_{k}}}}^{\ell}\left({\mathfrak{u}}_{i}\right)\right)}^{4}+{\left({\mathcal{T}}_{{\Lambda }_{\widehat{{ \varsigma }_{k}}}}^{\upsilon}\left({\mathfrak{u}}_{i}\right)\right)}^{4}+{\left({\mathcal{J}}_{{\Lambda }_{\widehat{{ \varsigma }_{k}}}}^{\ell}\left({\mathfrak{u}}_{i}\right)\right)}^{4}+{\left({\mathcal{J}}_{{\Lambda }_{\widehat{{ \varsigma }_{k}}}}^{\upsilon}\left({\mathfrak{u}}_{i}\right)\right)}^{4}\right)}\end{array}}\end{aligned}$$

$$={\mathbb{C}}_{IVPFHSS}\left(\left(\mathcal{G},\mathcal{B}\right), \left(\Lambda ,\mathcal{A}\right)\right)$$

### Proof 3.

As we know that

$$\begin{aligned}&{\mathbb{C}}_{IVPFHSS}\left(\left(\Lambda ,\mathcal{A}\right), \left(\mathcal{G},\mathcal{B}\right)\right)\\ &\quad=\frac{\sum_{k=1}^{m}\sum_{i=1}^{n}\left(\begin{array}{l}{\left({\mathcal{T}}_{{\Lambda }_{\widehat{{ \varsigma }_{k}}}}^{\ell}\left({\mathfrak{u}}_{i}\right)\right)}^{2}*{\left({\mathcal{T}}_{{\mathcal{G}}_{\widehat{{ \varsigma }_{k}}}}^{\ell}\left({\mathfrak{u}}_{i}\right)\right)}^{2}+{\left({\mathcal{T}}_{{\Lambda }_{\widehat{{ \varsigma }_{k}}}}^{\upsilon}\left({\mathfrak{u}}_{i}\right)\right)}^{2}*{\left({\mathcal{T}}_{{\mathcal{G}}_{\widehat{{ \varsigma }_{k}}}}^{\upsilon}\left({\mathfrak{u}}_{i}\right)\right)}^{2}\\ \quad+{\left({\mathcal{J}}_{{\Lambda }_{\widehat{{ \varsigma }_{k}}}}^{\ell}\left({\mathfrak{u}}_{i}\right)\right)}^{2}*{\left({\mathcal{J}}_{{\mathcal{G}}_{\widehat{{ \varsigma }_{k}}}}^{\ell}\left({\mathfrak{u}}_{i}\right)\right)}^{2}+{\left({\mathcal{J}}_{{\Lambda }_{\widehat{{ \varsigma }_{k}}}}^{\upsilon}\left({\mathfrak{u}}_{i}\right)\right)}^{2}*{\left({\mathcal{J}}_{{\mathcal{G}}_{\widehat{{ \varsigma }_{k}}}}^{\upsilon}\left({\mathfrak{u}}_{i}\right)\right)}^{2}\end{array}\right) }{\begin{array}{l}\sqrt{\sum_{k=1}^{m}\sum_{i=1}^{n}\left({\left({\mathcal{T}}_{{\Lambda }_{\widehat{{ \varsigma }_{k}}}}^{\ell}\left({\mathfrak{u}}_{i}\right)\right)}^{4}+{\left({\mathcal{T}}_{{\Lambda }_{\widehat{{ \varsigma }_{k}}}}^{\upsilon}\left({\mathfrak{u}}_{i}\right)\right)}^{4}+{\left({\mathcal{J}}_{{\Lambda }_{\widehat{{ \varsigma }_{k}}}}^{\ell}\left({\mathfrak{u}}_{i}\right)\right)}^{4}+{\left({\mathcal{J}}_{{\Lambda }_{\widehat{{ \varsigma }_{k}}}}^{\upsilon}\left({\mathfrak{u}}_{i}\right)\right)}^{4}\right)} \\ \quad \sqrt{\sum_{k=1}^{m}\sum_{i=1}^{n}\left({\left({\mathcal{T}}_{{\mathcal{G}}_{\widehat{{ \varsigma }_{k}}}}^{\ell}\left({\mathfrak{u}}_{i}\right)\right)}^{4}+{\left({\mathcal{T}}_{{\mathcal{G}}_{\widehat{{ \varsigma }_{k}}}}^{\upsilon}\left({\mathfrak{u}}_{i}\right)\right)}^{4}+{\left({\mathcal{J}}_{{\mathcal{G}}_{\widehat{{ \varsigma }_{k}}}}^{\ell}\left({\mathfrak{u}}_{i}\right)\right)}^{4}+{\left({\mathcal{J}}_{{\mathcal{G}}_{\widehat{{ \varsigma }_{k}}}}^{\upsilon}\left({\mathfrak{u}}_{i}\right)\right)}^{4}\right)} \end{array}}\end{aligned}$$

As

$${\mathcal{T}}_{{\Lambda }_{\widehat{{ \varsigma }_{0k}}}}^{\ell}\left({\mathfrak{u}}_{i}\right)={\mathcal{T}}_{{\mathcal{G}}_{\widehat{{ \varsigma }_{k}}}}^{\ell}\left({\mathfrak{u}}_{i}\right)$$

$${\mathcal{T}}_{{\Lambda }_{\widehat{{ \varsigma }_{k}}}}^{\upsilon}\left({\mathfrak{u}}_{i}\right)={\mathcal{T}}_{{\mathcal{G}}_{\widehat{{ \varsigma }_{k}}}}^{\upsilon}\left({\mathfrak{u}}_{i}\right)$$

$${\mathcal{J}}_{{\Lambda }_{\widehat{{ \varsigma }_{k}}}}^{\ell}\left({\mathfrak{u}}_{i}\right)={\mathcal{J}}_{{\mathcal{G}}_{\widehat{{ \varsigma }_{k}}}}^{\ell}\left({\mathfrak{u}}_{i}\right)$$

$${\mathcal{J}}_{{\Lambda }_{\widehat{{ \varsigma }_{k}}}}^{\upsilon}\left({\mathfrak{u}}_{i}\right)={\mathcal{J}}_{{\mathcal{G}}_{\widehat{{ \varsigma }_{k}}}}^{\upsilon}\left({\mathfrak{u}}_{i}\right)$$

$$\begin{aligned}&{\mathbb{C}}_{IVPFHSS}\left(\left(\Lambda ,\mathcal{A}\right), \left(\mathcal{G},\mathcal{B}\right)\right)\\&=\frac{\sum_{k=1}^{m}\sum_{i=1}^{n}\left({\left({\mathcal{T}}_{{\Lambda }_{\widehat{{ \varsigma }_{k}}}}^{\ell}\left({\mathfrak{u}}_{i}\right)\right)}^{4}+{\left({\mathcal{T}}_{{\Lambda }_{\widehat{{ \varsigma }_{k}}}}^{\upsilon}\left({\mathfrak{u}}_{i}\right)\right)}^{4}+{\left({\mathcal{J}}_{{\Lambda }_{\widehat{{ \varsigma }_{k}}}}^{\ell}\left({\mathfrak{u}}_{i}\right)\right)}^{4}+{\left({\mathcal{J}}_{{\Lambda }_{\widehat{{ \varsigma }_{k}}}}^{\upsilon}\left({\mathfrak{u}}_{i}\right)\right)}^{4}\right) }{\begin{array}{l}\sqrt{\sum_{k=1}^{m}\sum_{i=1}^{n}\left({\left({\mathcal{T}}_{{\Lambda }_{\widehat{{ \varsigma }_{k}}}}^{\ell}\left({\mathfrak{u}}_{i}\right)\right)}^{4}+{\left({\mathcal{T}}_{{\Lambda }_{\widehat{{ \varsigma }_{k}}}}^{\upsilon}\left({\mathfrak{u}}_{i}\right)\right)}^{4}+{\left({\mathcal{J}}_{{\Lambda }_{\widehat{{ \varsigma }_{k}}}}^{\ell}\left({\mathfrak{u}}_{i}\right)\right)}^{4}+{\left({\mathcal{J}}_{{\Lambda }_{\widehat{{ \varsigma }_{k}}}}^{\upsilon}\left({\mathfrak{u}}_{i}\right)\right)}^{4}\right)} \\ \quad \sqrt{\sum_{j=1}^{m}\left({\left({\mathcal{T}}_{{\Lambda }_{\widehat{{ \varsigma }_{k}}}}^{\ell}\left({\mathfrak{u}}_{i}\right)\right)}^{4}+{\left({\mathcal{T}}_{{\Lambda }_{\widehat{{ \varsigma }_{k}}}}^{\upsilon}\left({\mathfrak{u}}_{i}\right)\right)}^{4}+{\left({\mathcal{J}}_{{\Lambda }_{\widehat{{ \varsigma }_{k}}}}^{\ell}\left({\mathfrak{u}}_{i}\right)\right)}^{4}+{\left({\mathcal{J}}_{{\Lambda }_{\widehat{{ \varsigma }_{k}}}}^{\upsilon}\left({\mathfrak{u}}_{i}\right)\right)}^{4}\right)}\end{array}}\end{aligned}$$

$${\mathbb{C}}_{IVPFHSS}\left(\left(\Lambda ,\mathcal{A}\right),\left(\mathcal{G},\mathcal{B}\right)\right)=1.$$

### Definition 13.

Let $\left(\Lambda ,\mathcal{A}\right)=\left\{\left({\mathfrak{u}}_{i}, \left(\left[{\mathcal{T}}_{{\Lambda }_{\widehat{{ \varsigma }_{k}}}}^{\ell}\left({\mathfrak{u}}_{i}\right), {\mathcal{T}}_{{\Lambda }_{\widehat{{ \varsigma }_{k}}}}^{\upsilon}\left({\mathfrak{u}}_{i}\right)\right], \left[{\mathcal{J}}_{{\Lambda }_{\widehat{{ \varsigma }_{k}}}}^{\ell}\left({\mathfrak{u}}_{i}\right), {\mathcal{J}}_{{\Lambda }_{\widehat{{ \varsigma }_{k}}}}^{\upsilon}\left({\mathfrak{u}}_{i}\right)\right]\right)\right) \left. {} \right| {\mathfrak{u}}_{i}\in \mathsf{U}\right\}$ and $\left(\mathcal{G},\mathcal{B}\right)=\left\{\left({\mathfrak{u}}_{i}, \left(\left[{\mathcal{T}}_{{\mathcal{G}}_{\widehat{{ \varsigma }_{k}}}}^{\ell}\left({\mathfrak{u}}_{i}\right), {\mathcal{T}}_{{\mathcal{G}}_{\widehat{{ \varsigma }_{k}}}}^{\upsilon}\left({\mathfrak{u}}_{i}\right)\right], \left[{\mathcal{J}}_{{\mathcal{G}}_{\widehat{{ \varsigma }_{k}}}}^{\ell}\left({\mathfrak{u}}_{i}\right), {\mathcal{J}}_{{\mathcal{G}}_{\widehat{{ \varsigma }_{k}}}}^{\upsilon}\left({\mathfrak{u}}_{i}\right)\right]\right)\right) \left. {} \right| {\mathfrak{u}}_{i}\in \mathsf{U}\right\}$ be two IVPFHSS. Then, the CC between them is also defined as:

$${\mathbb{C}}_{IVPFHSS}^{1}\left(\left(\Lambda ,\mathcal{A}\right), \left(\mathcal{G},\mathcal{B}\right)\right)=\frac{{\mathcal{C}}_{IVPFHSS}\left(\left(\Lambda ,\mathcal{A}\right), \left(\mathcal{G},\mathcal{B}\right)\right)}{max\left\{{\mathcal{E}}_{IVPFHSS}\left(\Lambda ,\mathcal{A}\right), {\mathcal{E}}_{IVPFHSS}\left(\mathcal{G},\mathcal{B}\right)\right\}}$$

5 $$\begin{aligned}&{\mathbb{C}}_{IVPFHSS}^{1}\left(\left(\Lambda ,\mathcal{A}\right), \left(\mathcal{G},\mathcal{B}\right)\right)\\\quad&=\frac{\sum_{k=1}^{m}\sum_{i=1}^{n}\left(\begin{array}{l}{\left({\mathcal{T}}_{{\Lambda }_{\widehat{{ \varsigma }_{k}}}}^{\ell}\left({\mathfrak{u}}_{i}\right)\right)}^{2}*{\left({\mathcal{T}}_{{\mathcal{G}}_{\widehat{{ \varsigma }_{k}}}}^{\ell}\left({\mathfrak{u}}_{i}\right)\right)}^{2}+{\left({\mathcal{T}}_{{\Lambda }_{\widehat{{ \varsigma }_{k}}}}^{\upsilon}\left({\mathfrak{u}}_{i}\right)\right)}^{2}*{\left({\mathcal{T}}_{{\mathcal{G}}_{\widehat{{ \varsigma }_{k}}}}^{\upsilon}\left({\mathfrak{u}}_{i}\right)\right)}^{2}\\\quad+{\left({\mathcal{J}}_{{\Lambda }_{\widehat{{ \varsigma }_{k}}}}^{\ell}\left({\mathfrak{u}}_{i}\right)\right)}^{2}*{\left({\mathcal{J}}_{{\mathcal{G}}_{\widehat{{ \varsigma }_{k}}}}^{\ell}\left({\mathfrak{u}}_{i}\right)\right)}^{2}+{\left({\mathcal{J}}_{{\Lambda }_{\widehat{{ \varsigma }_{k}}}}^{\upsilon}\left({\mathfrak{u}}_{i}\right)\right)}^{2}*{\left({\mathcal{J}}_{{\mathcal{G}}_{\widehat{{ \varsigma }_{k}}}}^{\upsilon}\left({\mathfrak{u}}_{i}\right)\right)}^{2}\end{array}\right) }{max\left\{\begin{array}{l}\sum_{k=1}^{m}\sum_{i=1}^{n}\left({\left({\mathcal{T}}_{{\Lambda }_{\widehat{{ \varsigma }_{k}}}}^{\ell}\left({\mathfrak{u}}_{i}\right)\right)}^{4}+{\left({\mathcal{T}}_{{\Lambda }_{\widehat{{ \varsigma }_{k}}}}^{\upsilon}\left({\mathfrak{u}}_{i}\right)\right)}^{4}+{\left({\mathcal{J}}_{{\Lambda }_{\widehat{{ \varsigma }_{k}}}}^{\ell}\left({\mathfrak{u}}_{i}\right)\right)}^{4}+{\left({\mathcal{J}}_{{\Lambda }_{\widehat{{ \varsigma }_{k}}}}^{\upsilon}\left({\mathfrak{u}}_{i}\right)\right)}^{4}\right),\\\quad \sum_{k=1}^{m}\sum_{i=1}^{n}\left({\left({\mathcal{T}}_{{\mathcal{G}}_{\widehat{{ \varsigma }_{k}}}}^{\ell}\left({\mathfrak{u}}_{i}\right)\right)}^{4}+{\left({\mathcal{T}}_{{\mathcal{G}}_{\widehat{{ \varsigma }_{k}}}}^{\upsilon}\left({\mathfrak{u}}_{i}\right)\right)}^{4}+{\left({\mathcal{J}}_{{\mathcal{G}}_{\widehat{{ \varsigma }_{k}}}}^{\ell}\left({\mathfrak{u}}_{i}\right)\right)}^{4}+{\left({\mathcal{J}}_{{\mathcal{G}}_{\widehat{{ \varsigma }_{k}}}}^{\upsilon}\left({\mathfrak{u}}_{i}\right)\right)}^{4}\right)\end{array}\right\}}\end{aligned}$$

### Theorem 14

Let $\left( {\Lambda ,{\mathcal{A}}} \right) = \left\{ {\left. {\left( {{\mathfrak{u}}_{i},\left( {\left[ {{\mathcal{T}}_{{\Lambda_{{\widehat{{\varsigma_{k} }}}} }}^{\ell } \left( {{\mathfrak{u}}_{i} } \right),{ }{\mathcal{T}}_{{\Lambda_{{\widehat{{\varsigma_{k} }}}} }}^{\upsilon } \left( {{\mathfrak{u}}_{i} } \right)} \right], \left[ {{\mathcal{J}}_{{\Lambda_{{\widehat{{\varsigma_{k} }}}} }}^{\ell } \left( {{\mathfrak{u}}_{i} } \right),{ }{\mathcal{J}}_{{\Lambda_{{\widehat{{\varsigma_{k} }}}} }}^{\upsilon } \left( {{\mathfrak{u}}_{i} } \right)} \right]} \right)} \right) } \right|{\mathfrak{u}}_{i} \in {U}} \right\}$ and $\left( {{\mathcal{G}},{ \mathcal{B}}} \right) = \left\{ {\left. {\left( {{\mathfrak{u}}_{i},\left( {\left[ {{\mathcal{T}}_{{{\mathcal{G}}_{{\widehat{{\varsigma_{k} }}}} }}^{\ell } \left( {{\mathfrak{u}}_{i} } \right),{ }{\mathcal{T}}_{{{\mathcal{G}}_{{\widehat{{\varsigma_{k} }}}} }}^{\upsilon } \left( {{\mathfrak{u}}_{i} } \right)} \right], \left[ {{\mathcal{J}}_{{{\mathcal{G}}_{{\widehat{{\varsigma_{k} }}}} }}^{\ell } \left( {{\mathfrak{u}}_{i} } \right),{ }{\mathcal{J}}_{{{\mathcal{G}}_{{\widehat{{\varsigma_{k} }}}} }}^{\upsilon } \left( {{\mathfrak{u}}_{i} } \right)} \right]} \right)} \right) } \right|{\mathfrak{u}}_{i} \in {U}} \right\}$ be two IVPFHSS. Then the following properties hold:

1. $$0 \le {\mathbb{C}}_{IVPFHSS}^{1} \left( {\left( {\Lambda ,{\mathcal{A}}} \right),\left( {{\mathcal{G}},{ \mathcal{B}}} \right)} \right) \le 1$$.
2. $${\mathbb{C}}_{IVPFHSS}^{1} \left( {\left( {\Lambda ,{\mathcal{A}}} \right),\left( {{\mathcal{G}},{ \mathcal{B}}} \right)} \right) = {\mathbb{C}}_{IVPFHSS}^{1} \left( {\left( {{\mathcal{G}},{ \mathcal{B}}} \right),\left( {\Lambda ,{\mathcal{A}}} \right)} \right)$$
3. If ${\mathcal{T}}_{{\Lambda_{{\widehat{{\varsigma_{k} }}}} }}^{\ell } \left( {{\mathfrak{u}}_{i} } \right) = {\mathcal{T}}_{{{\mathcal{G}}_{{\widehat{{\varsigma_{k} }}}} }}^{\ell } \left( {{\mathfrak{u}}_{i} } \right)$, ${\mathcal{T}}_{{\Lambda_{{\widehat{{\varsigma_{k} }}}} }}^{\upsilon } \left( {{\mathfrak{u}}_{i} } \right) = {\mathcal{T}}_{{{\mathcal{G}}_{{\widehat{{\varsigma_{k} }}}} }}^{\upsilon } \left( {{\mathfrak{u}}_{i} } \right)$, ${\mathcal{J}}_{{\Lambda_{{\widehat{{\varsigma_{k} }}}} }}^{\ell } \left( {{\mathfrak{u}}_{i} } \right) = {\mathcal{J}}_{{{\mathcal{G}}_{{\widehat{{\varsigma_{k} }}}} }}^{\ell } \left( {{\mathfrak{u}}_{i} } \right)$, and ${\mathcal{J}}_{{\Lambda_{{\widehat{{\varsigma_{k} }}}} }}^{\upsilon } \left( {{\mathfrak{u}}_{i} } \right) = {\mathcal{J}}_{{{\mathcal{G}}_{{\widehat{{\varsigma_{k} }}}} }}^{\upsilon } \left( {{\mathfrak{u}}_{i} } \right)$ $\forall$ $i, k$. Then ${\mathbb{C}}_{IVPFHSS}^{1} \left( {\left( {\Lambda ,{\mathcal{A}}} \right),\left( {{\mathcal{G}},{ \mathcal{B}}} \right)} \right) = 1$.

### Proof.

TheIproofIof case 2 is straightforward, and case 3 is like Theorem 12, which involves case 3 as well. Also, it's apparent that. ${\mathbb{C}}_{IVPFHSS}^{1} \left( {\left( {\Lambda ,{\mathcal{A}}} \right),{ }\left( {{\mathcal{G}},{ \mathcal{B}}} \right)} \right) \ge 0$ in case 1. To complete the proof, we only need to show that ${\mathbb{C}}_{IVPFHSS}^{1} \left( {\left( {\Lambda ,{\mathcal{A}}} \right),{ }\left( {{\mathcal{G}},{ \mathcal{B}}} \right)} \right) \le 1$. This can be shown by using the inequality ${\mathcal{C}}_{IVPFHSS} \left( {\left( {\Lambda ,{\mathcal{A}}} \right),{ }\left( {{\mathcal{G}},{ \mathcal{B}}} \right)} \right)^{2} \le {\mathcal{E}}_{IVPFHSS} \left( {\Lambda ,{\mathcal{A}}} \right) \times {\mathcal{E}}_{IVPFHSS} \left( {{\mathcal{G}},{ \mathcal{B}}} \right)$. Therefore, ${\mathcal{C}}_{IVPFHSS} \left( {\left( {\Lambda ,{\mathcal{A}}} \right),\left( {{\mathcal{G}},{ \mathcal{B}}} \right)} \right) \le max\left\{ {{\mathcal{E}}_{IVPFHSS} \left( {\Lambda ,{\mathcal{A}}} \right), {\mathcal{E}}_{IVPFHSS} \left( {{\mathcal{G}},{ \mathcal{B}}} \right)} \right\}$. Hence, ${\mathbb{C}}_{IVPFHSS}^{1} \left( {\left( {\Lambda ,{\mathcal{A}}} \right),{ }\left( {{\mathcal{G}},{ \mathcal{B}}} \right)} \right) \le 1$.

Before making a final decision in real-life decision-making applications, examining the weight of factors and expert opinions is critical. This is particularly pertinent in IVPFHSS, while the weights designated to particular alternatives can considerably impact the outcomes. In this study, we propose a novel weighted correlation coefficient (WCC) for IVPFHSS that considers decision makers' and alternatives' weights.

## Weighted correlation coefficient for interval valued pythagorean fuzzy hypersoft set

The proposed method takes into account the weights of experts and parameters such as; $\Omega = \left\{ {\Omega _{1} ,{ }\Omega _{2} ,{ }\Omega _{3},\ldots ,{ }\Omega _{n} } \right\}^{T}$ and $\gamma = \left\{ {{\upgamma }_{1} ,{{ \gamma }}_{2} ,{{ \gamma }}_{3},\ldots ,{{ \gamma }}_{m} } \right\}^{T}$ be the weights for experts and parameters. Where, $\Omega _{i} > 0$, $\mathop \sum \nolimits_{i = 1}^{m}\Omega _{i} = 1$ and ${\upgamma }_{k} > 0$, $\mathop \sum \nolimits_{k = 1}^{m} {\upgamma }_{k} = 1$, they are ensuring that their sum is equal to one. By doing so, the WCC can provide a more accurate representation of the DM process.

### Definition 15

Let $\left( {\Lambda ,{\mathcal{A}}} \right) = \left\{ {\left. {\left( {{\mathfrak{u}}_{i},\left( {\left[ {{\mathcal{T}}_{{\Lambda_{{\widehat{{\varsigma_{k} }}}} }}^{\ell } \left( {{\mathfrak{u}}_{i} } \right),{ }{\mathcal{T}}_{{\Lambda_{{\widehat{{\varsigma_{k} }}}} }}^{\upsilon } \left( {{\mathfrak{u}}_{i} } \right)} \right], \left[ {{\mathcal{J}}_{{\Lambda_{{\widehat{{\varsigma_{k} }}}} }}^{\ell } \left( {{\mathfrak{u}}_{i} } \right),{ }{\mathcal{J}}_{{\Lambda_{{\widehat{{\varsigma_{k} }}}} }}^{\upsilon } \left( {{\mathfrak{u}}_{i} } \right)} \right]} \right)} \right)} \right| {\mathfrak{u}}_{i} \in {U}} \right\}$ and $\left( {{\mathcal{G}},{ \mathcal{B}}} \right) = \left\{ {\left. {\left( {{\mathfrak{u}}_{i},\left( {\left[ {{\mathcal{T}}_{{{\mathcal{G}}_{{\widehat{{\varsigma_{k} }}}} }}^{\ell } \left( {{\mathfrak{u}}_{i} } \right),{ }{\mathcal{T}}_{{{\mathcal{G}}_{{\widehat{{\varsigma_{k} }}}} }}^{\upsilon } \left( {{\mathfrak{u}}_{i} } \right)} \right], \left[ {{\mathcal{J}}_{{{\mathcal{G}}_{{\widehat{{\varsigma_{k} }}}} }}^{\ell } \left( {{\mathfrak{u}}_{i} } \right),{ }{\mathcal{J}}_{{{\mathcal{G}}_{{\widehat{{\varsigma_{k} }}}} }}^{\upsilon } \left( {{\mathfrak{u}}_{i} } \right)} \right]} \right)} \right) } \right|{\mathfrak{u}}_{i} \in {U}} \right\}$ be two IVPFHSS. Then, their weighted informational energies can be defined as:

6 $${\mathcal{E}}_{WIVPFHSS} \left( {\Lambda ,{\mathcal{A}}} \right) = \mathop \sum \limits_{k = 1}^{m} \gamma_{k} \left( {\mathop \sum \limits_{i = 1}^{n}\Omega _{i} \left( {\left( {{\mathcal{T}}_{{\Lambda_{{\widehat{{\varsigma_{k} }}}} }}^{\ell } \left( {{\mathfrak{u}}_{i} } \right)} \right)^{4} + \left( {{\mathcal{T}}_{{\Lambda_{{\widehat{{\varsigma_{k} }}}} }}^{\upsilon } \left( {{\mathfrak{u}}_{i} } \right)} \right)^{4} + \left( {{\mathcal{J}}_{{\Lambda_{{\widehat{{\varsigma_{k} }}}} }}^{\ell } \left( {{\mathfrak{u}}_{i} } \right)} \right)^{4} + \left( {{\mathcal{J}}_{{\Lambda_{{\widehat{{\varsigma_{k} }}}} }}^{\upsilon } \left( {{\mathfrak{u}}_{i} } \right)} \right)^{4} } \right)} \right)$$

7 $${\mathcal{E}}_{WIVPFHSS}\left(\mathcal{G},\mathcal{B}\right)=\sum_{k=1}^{m}{\gamma }_{k}\left(\sum_{i=1}^{n}{\Omega }_{i}\left({\left({\mathcal{T}}_{{\mathcal{G}}_{\widehat{{ \varsigma }_{k}}}}^{\ell}\left({\mathfrak{u}}_{i}\right)\right)}^{4}+{\left({\mathcal{T}}_{{\mathcal{G}}_{\widehat{{ \varsigma }_{k}}}}^{\upsilon}\left({\mathfrak{u}}_{i}\right)\right)}^{4}+{\left({\mathcal{J}}_{{\mathcal{G}}_{\widehat{{ \varsigma }_{k}}}}^{\ell}\left({\mathfrak{u}}_{i}\right)\right)}^{4}+{\left({\mathcal{J}}_{{\mathcal{G}}_{\widehat{{ \varsigma }_{k}}}}^{\upsilon}\left({\mathfrak{u}}_{i}\right)\right)}^{4}\right)\right)$$

### Definition 16

Let $\left(\Lambda ,\mathcal{A}\right)=\left\{\left({\mathfrak{u}}_{i}, \left(\left[{\mathcal{T}}_{{\Lambda }_{\widehat{{ \varsigma }_{k}}}}^{\ell}\left({\mathfrak{u}}_{i}\right), {\mathcal{T}}_{{\Lambda }_{\widehat{{ \varsigma }_{k}}}}^{\upsilon}\left({\mathfrak{u}}_{i}\right)\right], \left[{\mathcal{J}}_{{\Lambda }_{\widehat{{ \varsigma }_{k}}}}^{\ell}\left({\mathfrak{u}}_{i}\right), {\mathcal{J}}_{{\Lambda }_{\widehat{{ \varsigma }_{k}}}}^{\upsilon}\left({\mathfrak{u}}_{i}\right)\right]\right)\right) \left. {} \right| {\mathfrak{u}}_{i}\in \mathsf{U}\right\}$ and $\left(\mathcal{G},\mathcal{B}\right)=\left\{\left({\mathfrak{u}}_{i}, \left(\left[{\mathcal{T}}_{{\mathcal{G}}_{\widehat{{ \varsigma }_{k}}}}^{\ell}\left({\mathfrak{u}}_{i}\right), {\mathcal{T}}_{{\mathcal{G}}_{\widehat{{ \varsigma }_{k}}}}^{\upsilon}\left({\mathfrak{u}}_{i}\right)\right], \left[{\mathcal{J}}_{{\mathcal{G}}_{\widehat{{ \varsigma }_{k}}}}^{\ell}\left({\mathfrak{u}}_{i}\right), {\mathcal{J}}_{{\mathcal{G}}_{\widehat{{ \varsigma }_{k}}}}^{\upsilon}\left({\mathfrak{u}}_{i}\right)\right]\right)\right) \left. {} \right| {\mathfrak{u}}_{i}\in \mathsf{U}\right\}$ be two IVPFHSS. Then, the weighted correlation between them can be defined as:

8 $${\mathcal{C}}_{WIVPFSS}\left(\left(\mathcal{F},\mathcal{A}\right), \left(\mathcal{G},\mathcal{B}\right)\right)=\sum_{k=1}^{m}{\gamma }_{k}\left(\sum_{i=1}^{n}{\Omega }_{i}\left(\begin{array}{c}{\left({\mathcal{T}}_{{\Lambda }_{\widehat{{ \varsigma }_{k}}}}^{\ell}\left({\mathfrak{u}}_{i}\right)\right)}^{2}*{\left({\mathcal{T}}_{{\mathcal{G}}_{\widehat{{ \varsigma }_{k}}}}^{\ell}\left({\mathfrak{u}}_{i}\right)\right)}^{2}+{\left({\mathcal{T}}_{{\Lambda }_{\widehat{{ \varsigma }_{k}}}}^{\upsilon}\left({\mathfrak{u}}_{i}\right)\right)}^{2}*{\left({\mathcal{T}}_{{\mathcal{G}}_{\widehat{{ \varsigma }_{k}}}}^{\upsilon}\left({\mathfrak{u}}_{i}\right)\right)}^{2}+\\ {\left({\mathcal{J}}_{{\Lambda }_{\widehat{{ \varsigma }_{k}}}}^{\ell}\left({\mathfrak{u}}_{i}\right)\right)}^{2}*{\left({\mathcal{J}}_{{\mathcal{G}}_{\widehat{{ \varsigma }_{k}}}}^{\ell}\left({\mathfrak{u}}_{i}\right)\right)}^{2}+{\left({\mathcal{J}}_{{\Lambda }_{\widehat{{ \varsigma }_{k}}}}^{\upsilon}\left({\mathfrak{u}}_{i}\right)\right)}^{2}*{\left({\mathcal{J}}_{{\mathcal{G}}_{\widehat{{ \varsigma }_{k}}}}^{\upsilon}\left({\mathfrak{u}}_{i}\right)\right)}^{2}\end{array}\right)\right)$$

### Proposition 17

Let $\left(\Lambda ,\mathcal{A}\right)=\left\{\left({\mathfrak{u}}_{i}, \left(\left[{\mathcal{T}}_{{\Lambda }_{\widehat{{ \varsigma }_{k}}}}^{\ell}\left({\mathfrak{u}}_{i}\right), {\mathcal{T}}_{{\Lambda }_{\widehat{{ \varsigma }_{k}}}}^{\upsilon}\left({\mathfrak{u}}_{i}\right)\right], \left[{\mathcal{J}}_{{\Lambda }_{\widehat{{ \varsigma }_{k}}}}^{\ell}\left({\mathfrak{u}}_{i}\right), {\mathcal{J}}_{{\Lambda }_{\widehat{{ \varsigma }_{k}}}}^{\upsilon}\left({\mathfrak{u}}_{i}\right)\right]\right)\right) \left. {} \right| {\mathfrak{u}}_{i}\in \mathsf{U}\right\}$ and $\left(\mathcal{G},\mathcal{B}\right)=\left\{\left({\mathfrak{u}}_{i}, \left(\left[{\mathcal{T}}_{{\mathcal{G}}_{\widehat{{ \varsigma }_{k}}}}^{\ell}\left({\mathfrak{u}}_{i}\right), {\mathcal{T}}_{{\mathcal{G}}_{\widehat{{ \varsigma }_{k}}}}^{\upsilon}\left({\mathfrak{u}}_{i}\right)\right], \left[{\mathcal{J}}_{{\mathcal{G}}_{\widehat{{ \varsigma }_{k}}}}^{\ell}\left({\mathfrak{u}}_{i}\right), {\mathcal{J}}_{{\mathcal{G}}_{\widehat{{ \varsigma }_{k}}}}^{\upsilon}\left({\mathfrak{u}}_{i}\right)\right]\right)\right) \left. {} \right| {\mathfrak{u}}_{i}\in \mathsf{U}\right\}$ be two IVPFHSS. Then

1. $${\mathcal{C}}_{WIVPFHSS}\left(\left(\Lambda ,\mathcal{A}\right),\left(\Lambda ,\mathcal{A}\right)\right)={\mathcal{E}}_{WIVPFHSS}\left(\Lambda ,\mathcal{A}\right)$$
2. $${\mathcal{C}}_{WIVPFHSS}\left(\left(\Lambda ,\mathcal{A}\right),\left(\mathcal{G},\mathcal{B}\right)\right)={\mathcal{C}}_{WIVPFHSS}\left(\left(\mathcal{G},\mathcal{B}\right),\left(\Lambda ,\mathcal{A}\right)\right)$$

### Proof:

The proof of the above-stated proposition is straightforward.

### Definition 18

Let $\left(\Lambda ,\mathcal{A}\right)=\left\{\left({\mathfrak{u}}_{i}, \left(\left[{\mathcal{T}}_{{\Lambda }_{\widehat{{ \varsigma }_{k}}}}^{\ell}\left({\mathfrak{u}}_{i}\right), {\mathcal{T}}_{{\Lambda }_{\widehat{{ \varsigma }_{k}}}}^{\upsilon}\left({\mathfrak{u}}_{i}\right)\right], \left[{\mathcal{J}}_{{\Lambda }_{\widehat{{ \varsigma }_{k}}}}^{\ell}\left({\mathfrak{u}}_{i}\right), {\mathcal{J}}_{{\Lambda }_{\widehat{{ \varsigma }_{k}}}}^{\upsilon}\left({\mathfrak{u}}_{i}\right)\right]\right)\right) \left. {} \right| {\mathfrak{u}}_{i}\in \mathsf{U}\right\}$ and $\left(\mathcal{G},\mathcal{B}\right)=\left\{\left({\mathfrak{u}}_{i}, \left(\left[{\mathcal{T}}_{{\mathcal{G}}_{\widehat{{ \varsigma }_{k}}}}^{\ell}\left({\mathfrak{u}}_{i}\right), {\mathcal{T}}_{{\mathcal{G}}_{\widehat{{ \varsigma }_{k}}}}^{\upsilon}\left({\mathfrak{u}}_{i}\right)\right], \left[{\mathcal{J}}_{{\mathcal{G}}_{\widehat{{ \varsigma }_{k}}}}^{\ell}\left({\mathfrak{u}}_{i}\right), {\mathcal{J}}_{{\mathcal{G}}_{\widehat{{ \varsigma }_{k}}}}^{\upsilon}\left({\mathfrak{u}}_{i}\right)\right]\right)\right) \left. {} \right| {\mathfrak{u}}_{i}\in \mathsf{U}\right\}$ be two IVPFHSS. Then, the WCC between them can be defined as:

9 $$\begin{aligned} & {\mathbb{C}}_{WIVPFHSS}\left(\left(\Lambda ,\mathcal{A}\right), \left(\mathcal{G},\mathcal{B}\right)\right)\\ & \quad =\frac{{\mathcal{C}}_{WIVPFHSS}\left(\left(\Lambda ,\mathcal{A}\right), \left(\mathcal{G},\mathcal{B}\right)\right)}{\sqrt{{\mathcal{E}}_{WIVPFHSS}\left(\Lambda ,\mathcal{A}\right)}\sqrt{{\mathcal{E}}_{WIVPFHSS}\left(\mathcal{G},\mathcal{B}\right)}}\\&\quad=\frac{\sum_{k=1}^{m}{\gamma }_{k}\left(\sum_{i=1}^{n}{\Omega }_{i}\left(\begin{array}{l}{\left({\mathcal{T}}_{{\Lambda }_{\widehat{{ \varsigma }_{k}}}}^{\ell}\left({\mathfrak{u}}_{i}\right)\right)}^{2}*{\left({\mathcal{T}}_{{\mathcal{G}}_{\widehat{{ \varsigma }_{k}}}}^{\ell}\left({\mathfrak{u}}_{i}\right)\right)}^{2}+{\left({\mathcal{T}}_{{\Lambda }_{\widehat{{ \varsigma }_{k}}}}^{\upsilon}\left({\mathfrak{u}}_{i}\right)\right)}^{2}*{\left({\mathcal{T}}_{{\mathcal{G}}_{\widehat{{ \varsigma }_{k}}}}^{\upsilon}\left({\mathfrak{u}}_{i}\right)\right)}^{2}\\ \quad+{\left({\mathcal{J}}_{{\Lambda }_{\widehat{{ \varsigma }_{k}}}}^{\ell}\left({\mathfrak{u}}_{i}\right)\right)}^{2}*{\left({\mathcal{J}}_{{\mathcal{G}}_{\widehat{{ \varsigma }_{k}}}}^{\ell}\left({\mathfrak{u}}_{i}\right)\right)}^{2}+{\left({\mathcal{J}}_{{\Lambda }_{\widehat{{ \varsigma }_{k}}}}^{\upsilon}\left({\mathfrak{u}}_{i}\right)\right)}^{2}*{\left({\mathcal{J}}_{{\mathcal{G}}_{\widehat{{ \varsigma }_{k}}}}^{\upsilon}\left({\mathfrak{u}}_{i}\right)\right)}^{2}\end{array}\right)\right)}{\begin{array}{l}\sqrt{\sum_{k=1}^{m}{\gamma }_{k}\left(\sum_{i=1}^{n}{\Omega }_{i}\left({\left({\mathcal{T}}_{{\Lambda }_{\widehat{{ \varsigma }_{k}}}}^{\ell}\left({\mathfrak{u}}_{i}\right)\right)}^{4}+{\left({\mathcal{T}}_{{\Lambda }_{\widehat{{ \varsigma }_{k}}}}^{\upsilon}\left({\mathfrak{u}}_{i}\right)\right)}^{4}+{\left({\mathcal{J}}_{{\Lambda }_{\widehat{{ \varsigma }_{k}}}}^{\ell}\left({\mathfrak{u}}_{i}\right)\right)}^{4}+{\left({\mathcal{J}}_{{\Lambda }_{\widehat{{ \varsigma }_{k}}}}^{\upsilon}\left({\mathfrak{u}}_{i}\right)\right)}^{4}\right)\right)} \\ \quad \sqrt{\sum_{k=1}^{m}{\gamma }_{k}\left(\sum_{i=1}^{n}{\Omega }_{i}\left({\left({\mathcal{T}}_{{\mathcal{G}}_{\widehat{{ \varsigma }_{k}}}}^{\ell}\left({\mathfrak{u}}_{i}\right)\right)}^{4}+{\left({\mathcal{T}}_{{\mathcal{G}}_{\widehat{{ \varsigma }_{k}}}}^{\upsilon}\left({\mathfrak{u}}_{i}\right)\right)}^{4}+{\left({\mathcal{J}}_{{\mathcal{G}}_{\widehat{{ \varsigma }_{k}}}}^{\ell}\left({\mathfrak{u}}_{i}\right)\right)}^{4}+{\left({\mathcal{J}}_{{\mathcal{G}}_{\widehat{{ \varsigma }_{k}}}}^{\upsilon}\left({\mathfrak{u}}_{i}\right)\right)}^{4}\right)\right)}\end{array}}\end{aligned}$$

### Theorem 19

Let $\left(\Lambda ,\mathcal{A}\right)=\left\{\left({\mathfrak{u}}_{i}, \left(\left[{\mathcal{T}}_{{\Lambda }_{\widehat{{ \varsigma }_{k}}}}^{\ell}\left({\mathfrak{u}}_{i}\right), {\mathcal{T}}_{{\Lambda }_{\widehat{{ \varsigma }_{k}}}}^{\upsilon}\left({\mathfrak{u}}_{i}\right)\right], \left[{\mathcal{J}}_{{\Lambda }_{\widehat{{ \varsigma }_{k}}}}^{\ell}\left({\mathfrak{u}}_{i}\right), {\mathcal{J}}_{{\Lambda }_{\widehat{{ \varsigma }_{k}}}}^{\upsilon}\left({\mathfrak{u}}_{i}\right)\right]\right)\right) \left. {} \right| {\mathfrak{u}}_{i}\in \mathsf{U}\right\}$ and $\left(\mathcal{G},\mathcal{B}\right)=\left\{\left({\mathfrak{u}}_{i}, \left(\left[{\mathcal{T}}_{{\mathcal{G}}_{\widehat{{ \varsigma }_{k}}}}^{\ell}\left({\mathfrak{u}}_{i}\right), {\mathcal{T}}_{{\mathcal{G}}_{\widehat{{ \varsigma }_{k}}}}^{\upsilon}\left({\mathfrak{u}}_{i}\right)\right], \left[{\mathcal{J}}_{{\mathcal{G}}_{\widehat{{ \varsigma }_{k}}}}^{\ell}\left({\mathfrak{u}}_{i}\right), {\mathcal{J}}_{{\mathcal{G}}_{\widehat{{ \varsigma }_{k}}}}^{\upsilon}\left({\mathfrak{u}}_{i}\right)\right]\right)\right) \left. {} \right| {\mathfrak{u}}_{i}\in \mathsf{U}\right\}$ be two IVPFHSS. If $\Omega ={\left\{{\Omega }_{1}, {\Omega }_{2}, {\Omega }_{3},\dots,{\Omega }_{n}\right\}}^{T}$ be a weight vector for experts and $\gamma ={\left\{{\gamma }_{1}, {\gamma }_{2}, {\gamma }_{3},\dots,{\gamma }_{m}\right\}}^{T}$ be the weight vector for attributes, such as ${\Omega }_{i}>0$*,* $\sum_{i=1}^{n}{\Omega }_{i}=1$ and ${\gamma }_{k}>0$*,* $\sum_{k=1}^{m}{\gamma }_{k}=1$. Then, the WCC satisfied the subsequent properties:

1. 0 $$\le$$ $${\mathbb{C}}_{WIVPFHSS}\left(\left(\Lambda ,\mathcal{A}\right),\left(\mathcal{G},\mathcal{B}\right)\right)$$ $$\le$$ 1
2. $${\mathbb{C}}_{WIVPFHSS}\left(\left(\Lambda ,\mathcal{A}\right),\left(\mathcal{G},\mathcal{B}\right)\right)={\mathbb{C}}_{WIVPFHSS}\left( \left(\mathcal{G},\mathcal{B}\right),\left(\Lambda ,\mathcal{A}\right)\right)$$
3. If $\left(\Lambda ,\mathcal{A}\right)=\left(\mathcal{G},\mathcal{B}\right)$, i.e., $\forall$ $i, k$ ${\mathcal{T}}_{{\Lambda }_{\widehat{{{{ \varsigma }}}_{k}}}}^{\ell}\left({\mathfrak{u}}_{i}\right)={\mathcal{T}}_{{\mathcal{G}}_{\widehat{{{{ \varsigma }}}_{k}}}}^{\ell}\left({\mathfrak{u}}_{i}\right)$, ${\mathcal{T}}_{{\Lambda }_{\widehat{{{{ \varsigma }}}_{k}}}}^{{{\upsilon}}}\left({\mathfrak{u}}_{i}\right)={\mathcal{T}}_{{\mathcal{G}}_{\widehat{{{{ \varsigma }}}_{k}}}}^{{{\upsilon}}}\left({\mathfrak{u}}_{i}\right)$, ${\mathcal{J}}_{{\Lambda }_{\widehat{{{{ \varsigma }}}_{k}}}}^{\ell}\left({\mathfrak{u}}_{i}\right)={\mathcal{J}}_{{\mathcal{G}}_{\widehat{{{{ \varsigma }}}_{k}}}}^{\ell}\left({\mathfrak{u}}_{i}\right)$, and ${\mathcal{J}}_{{\Lambda }_{\widehat{{{{ \varsigma }}}_{k}}}}^{{{\upsilon}}}\left({\mathfrak{u}}_{i}\right)={\mathcal{J}}_{{\mathcal{G}}_{\widehat{{{{ \varsigma }}}_{k}}}}^{{{\upsilon}}}\left({\mathfrak{u}}_{i}\right)$, then ${\mathbb{C}}_{WIVPFHSS}\left(\left(\Lambda ,\mathcal{A}\right),\left(\mathcal{G},\mathcal{B}\right)\right)=1$.

### Proof 1.

${\mathbb{C}}_{WIVPFHSS}\left(\left(\Lambda ,\mathcal{A}\right), \left(\mathcal{G},\mathcal{B}\right)\right)\ge 0$ is obvious. Now,IweIwillIdemonstrate ${\mathbb{C}}_{WIVPFHSS}\left(\left(\Lambda ,\mathcal{A}\right),\left(\mathcal{G},\mathcal{B}\right)\right)\le 1$. Using Eq. (8).

$${\mathcal{C}}_{WIVPFHSS}\left(\left(\Lambda ,\mathcal{A}\right),\left(\mathcal{G},\mathcal{B}\right)\right)=$$

$$\begin{aligned} & \sum_{k=1}^{m}{\gamma }_{k}\left(\sum_{i=1}^{n}{\Omega }_{i}\left(\begin{array}{c}{\left({\mathcal{T}}_{{\Lambda }_{\widehat{{ \varsigma }_{k}}}}^{\ell}\left({\mathfrak{u}}_{i}\right)\right)}^{2}*{\left({\mathcal{T}}_{{\mathcal{G}}_{\widehat{{ \varsigma }_{k}}}}^{\ell}\left({\mathfrak{u}}_{i}\right)\right)}^{2}+{\left({\mathcal{T}}_{{\Lambda }_{\widehat{{ \varsigma }_{k}}}}^{\upsilon}\left({\mathfrak{u}}_{i}\right)\right)}^{2}*{\left({\mathcal{T}}_{{\mathcal{G}}_{\widehat{{ \varsigma }_{k}}}}^{\upsilon}\left({\mathfrak{u}}_{i}\right)\right)}^{2}\end{array}\right.\right.\\&\quad \left. \left. + {\left({\mathcal{J}}_{{\Lambda }_{\widehat{{ \varsigma }_{k}}}}^{\ell}\left({\mathfrak{u}}_{i}\right)\right)}^{2}*{\left({\mathcal{J}}_{{\mathcal{G}}_{\widehat{{ \varsigma }_{k}}}}^{\ell}\left({\mathfrak{u}}_{i}\right)\right)}^{2} +{\left({\mathcal{J}}_{{\Lambda }_{\widehat{{ \varsigma }_{k}}}}^{\upsilon}\left({\mathfrak{u}}_{i}\right)\right)}^{2}*{\left({\mathcal{J}}_{{\mathcal{G}}_{\widehat{{ \varsigma }_{k}}}}^{\upsilon}\left({\mathfrak{u}}_{i}\right)\right)}^{2}\right)\right)\end{aligned}$$

$$\begin{aligned} & =\sum_{k=1}^{m}{\gamma }_{k}\left({\Omega }_{1}{\left({\mathcal{T}}_{{\Lambda }_{\widehat{{ \varsigma }_{k}}}}^{\ell}\left({\mathfrak{u}}_{1}\right)\right)}^{2}*{\left({\mathcal{T}}_{{\mathcal{G}}_{\widehat{{ \varsigma }_{k}}}}^{\ell}\left({\mathfrak{u}}_{1}\right)\right)}^{2}+{\left({\mathcal{T}}_{{\Lambda }_{\widehat{{ \varsigma }_{k}}}}^{\upsilon}\left({\mathfrak{u}}_{1}\right)\right)}^{2}*{\left({\mathcal{T}}_{{\mathcal{G}}_{\widehat{{ \varsigma }_{k}}}}^{\upsilon}\left({\mathfrak{u}}_{1}\right)\right)}^{2}\right.\\&\quad \left.+{\left({\mathcal{J}}_{{\Lambda }_{\widehat{{ \varsigma }_{k}}}}^{\ell}\left({\mathfrak{u}}_{1}\right)\right)}^{2}*{\left({\mathcal{J}}_{{\mathcal{G}}_{\widehat{{ \varsigma }_{k}}}}^{\ell}\left({\mathfrak{u}}_{1}\right)\right)}^{2}+{\left({\mathcal{J}}_{{\Lambda }_{\widehat{{ \varsigma }_{k}}}}^{\upsilon}\left({\mathfrak{u}}_{1}\right)\right)}^{2}*{\left({\mathcal{J}}_{{\mathcal{G}}_{\widehat{{ \varsigma }_{k}}}}^{\upsilon}\left({\mathfrak{u}}_{1}\right)\right)}^{2}\right)+\end{aligned}$$

$$\begin{aligned} & \sum_{k=1}^{m}{\gamma }_{k}\left({\Omega }_{2}\left({\left({\mathcal{T}}_{{\Lambda }_{\widehat{{ \varsigma }_{k}}}}^{\ell}\left({\mathfrak{u}}_{2}\right)\right)}^{2}*{\left({\mathcal{T}}_{{\mathcal{G}}_{\widehat{{ \varsigma }_{k}}}}^{\ell}\left({\mathfrak{u}}_{2}\right)\right)}^{2}+{\left({\mathcal{T}}_{{\Lambda }_{\widehat{{ \varsigma }_{k}}}}^{\upsilon}\left({\mathfrak{u}}_{2}\right)\right)}^{2}*{\left({\mathcal{T}}_{{\mathcal{G}}_{\widehat{{ \varsigma }_{k}}}}^{\upsilon}\left({\mathfrak{u}}_{2}\right)\right)}^{2}\right.\right.\\ & \quad\left.\left.+{\left({\mathcal{J}}_{{\Lambda }_{\widehat{{ \varsigma }_{k}}}}^{\ell}\left({\mathfrak{u}}_{2}\right)\right)}^{2}*{\left({\mathcal{J}}_{{\mathcal{G}}_{\widehat{{ \varsigma }_{k}}}}^{\ell}\left({\mathfrak{u}}_{2}\right)\right)}^{2}+{\left({\mathcal{J}}_{{\Lambda }_{\widehat{{ \varsigma }_{k}}}}^{\upsilon}\left({\mathfrak{u}}_{2}\right)\right)}^{2}*{\left({\mathcal{J}}_{{\mathcal{G}}_{\widehat{{ \varsigma }_{k}}}}^{\upsilon}\left({\mathfrak{u}}_{2}\right)\right)}^{2}\right)\right)\end{aligned}$$

$$+$$

$$\vdots$$

$$+$$

$$\begin{aligned} & \sum_{k=1}^{m}{\gamma }_{k}\left({\Omega }_{n}\left({\left({\mathcal{T}}_{{\Lambda }_{\widehat{{ \varsigma }_{k}}}}^{\ell}\left({\mathfrak{u}}_{n}\right)\right)}^{2}*{\left({\mathcal{T}}_{{\mathcal{G}}_{\widehat{{ \varsigma }_{k}}}}^{\ell}\left({\mathfrak{u}}_{n}\right)\right)}^{2}+{\left({\mathcal{T}}_{{\Lambda }_{\widehat{{ \varsigma }_{k}}}}^{\upsilon}\left({\mathfrak{u}}_{n}\right)\right)}^{2}*{\left({\mathcal{T}}_{{\mathcal{G}}_{\widehat{{ \varsigma }_{k}}}}^{\upsilon}\left({\mathfrak{u}}_{n}\right)\right)}^{2}\right.\right.\\ & \quad\left.\left.+{\left({\mathcal{J}}_{{\Lambda }_{\widehat{{ \varsigma }_{k}}}}^{\ell}\left({\mathfrak{u}}_{n}\right)\right)}^{2}*{\left({\mathcal{J}}_{{\mathcal{G}}_{\widehat{{ \varsigma }_{k}}}}^{\ell}\left({\mathfrak{u}}_{n}\right)\right)}^{2}+{\left({\mathcal{J}}_{{\Lambda }_{\widehat{{ \varsigma }_{k}}}}^{\upsilon}\left({\mathfrak{u}}_{n}\right)\right)}^{2}*{\left({\mathcal{J}}_{{\mathcal{G}}_{\widehat{{ \varsigma }_{k}}}}^{\upsilon}\left({\mathfrak{u}}_{n}\right)\right)}^{2}\right)\right)\end{aligned}$$

$$\begin{aligned} & {\mathcal{C}}_{WIVPFHSS}\left(\left(\Lambda ,\mathcal{A}\right), \left(\mathcal{G},\mathcal{B}\right)\right) \\ & \quad = \left\{ \begin{array}{c}{\gamma }_{1}\left({\Omega }_{1}\left(\begin{array}{l}{\left({\mathcal{T}}_{{\Lambda }_{\widehat{{ \varsigma }_{1}}}}^{\ell}\left({\mathfrak{u}}_{1}\right)\right)}^{2}*{\left({\mathcal{T}}_{{\mathcal{G}}_{\widehat{{ \varsigma }_{1}}}}^{\ell}\left({\mathfrak{u}}_{1}\right)\right)}^{2}+{\left({\mathcal{T}}_{{\Lambda }_{\widehat{{ \varsigma }_{1}}}}^{\upsilon}\left({\mathfrak{u}}_{1}\right)\right)}^{2}*{\left({\mathcal{T}}_{{\mathcal{G}}_{\widehat{{ \varsigma }_{1}}}}^{\upsilon}\left({\mathfrak{u}}_{1}\right)\right)}^{2}\\ \quad+{\left({\mathcal{J}}_{{\Lambda }_{\widehat{{ \varsigma }_{1}}}}^{\ell}\left({\mathfrak{u}}_{1}\right)\right)}^{2}*{\left({\mathcal{J}}_{{\mathcal{G}}_{\widehat{{ \varsigma }_{1}}}}^{\ell}\left({\mathfrak{u}}_{1}\right)\right)}^{2}+{\left({\mathcal{J}}_{{\Lambda }_{\widehat{{ \varsigma }_{1}}}}^{\upsilon}\left({\mathfrak{u}}_{1}\right)\right)}^{2}*{\left({\mathcal{J}}_{{\mathcal{G}}_{\widehat{{ \varsigma }_{1}}}}^{\upsilon}\left({\mathfrak{u}}_{1}\right)\right)}^{2}\end{array}\right)\right)+\\ {\gamma }_{2}\left({\Omega }_{1}\left(\begin{array}{l}{\left({\mathcal{T}}_{{\Lambda }_{\widehat{{ \varsigma }_{2}}}}^{\ell}\left({\mathfrak{u}}_{1}\right)\right)}^{2}*{\left({\mathcal{T}}_{{\mathcal{G}}_{\widehat{{ \varsigma }_{2}}}}^{\ell}\left({\mathfrak{u}}_{1}\right)\right)}^{2}+{\left({\mathcal{T}}_{{\Lambda }_{\widehat{{ \varsigma }_{2}}}}^{\upsilon}\left({\mathfrak{u}}_{1}\right)\right)}^{2}*{\left({\mathcal{T}}_{{\mathcal{G}}_{\widehat{{ \varsigma }_{2}}}}^{\upsilon}\left({\mathfrak{u}}_{1}\right)\right)}^{2}\\ \quad +{\left({\mathcal{J}}_{{\Lambda }_{\widehat{{ \varsigma }_{2}}}}^{\ell}\left({\mathfrak{u}}_{1}\right)\right)}^{2}*{\left({\mathcal{J}}_{{\mathcal{G}}_{\widehat{{ \varsigma }_{2}}}}^{\ell}\left({\mathfrak{u}}_{1}\right)\right)}^{2}+{\left({\mathcal{J}}_{{\Lambda }_{\widehat{{ \varsigma }_{2}}}}^{\upsilon}\left({\mathfrak{u}}_{1}\right)\right)}^{2}*{\left({\mathcal{J}}_{{\mathcal{G}}_{\widehat{{ \varsigma }_{2}}}}^{\upsilon}\left({\mathfrak{u}}_{1}\right)\right)}^{2}\end{array}\right)\right)+\\ \vdots \\ +\\ {\gamma }_{m}\left({\Omega }_{1}\left(\begin{array}{l}{\left({\mathcal{T}}_{{\Lambda }_{\widehat{{ \varsigma }_{m}}}}^{\ell}\left({\mathfrak{u}}_{1}\right)\right)}^{2}*{\left({\mathcal{T}}_{{\mathcal{G}}_{\widehat{{ \varsigma }_{m}}}}^{\ell}\left({\mathfrak{u}}_{1}\right)\right)}^{2}+{\left({\mathcal{T}}_{{\Lambda }_{\widehat{{ \varsigma }_{m}}}}^{\upsilon}\left({\mathfrak{u}}_{1}\right)\right)}^{2}*{\left({\mathcal{T}}_{{\mathcal{G}}_{\widehat{{ \varsigma }_{m}}}}^{\upsilon}\left({\mathfrak{u}}_{1}\right)\right)}^{2}\\ \quad +{\left({\mathcal{J}}_{{\Lambda }_{\widehat{{ \varsigma }_{m}}}}^{\ell}\left({\mathfrak{u}}_{1}\right)\right)}^{2}*{\left({\mathcal{J}}_{{\mathcal{G}}_{\widehat{{ \varsigma }_{m}}}}^{\ell}\left({\mathfrak{u}}_{1}\right)\right)}^{2}+{\left({\mathcal{J}}_{{\Lambda }_{\widehat{{ \varsigma }_{m}}}}^{\upsilon}\left({\mathfrak{u}}_{1}\right)\right)}^{2}*{\left({\mathcal{J}}_{{\mathcal{G}}_{\widehat{{ \varsigma }_{m}}}}^{\upsilon}\left({\mathfrak{u}}_{1}\right)\right)}^{2}\end{array}\right)\right)\end{array}\right\}\end{aligned}$$

$$+$$

$$\left\{\begin{array}{c}{\gamma }_{1}\left({\Omega }_{2}\left(\begin{array}{c}{\left({\mathcal{T}}_{{\Lambda }_{\widehat{{ \varsigma }_{1}}}}^{\ell}\left({\mathfrak{u}}_{2}\right)\right)}^{2}*{\left({\mathcal{T}}_{{\mathcal{G}}_{\widehat{{ \varsigma }_{1}}}}^{\ell}\left({\mathfrak{u}}_{2}\right)\right)}^{2}+{\left({\mathcal{T}}_{{\Lambda }_{\widehat{{ \varsigma }_{1}}}}^{\upsilon}\left({\mathfrak{u}}_{2}\right)\right)}^{2}*{\left({\mathcal{T}}_{{\mathcal{G}}_{\widehat{{ \varsigma }_{1}}}}^{\upsilon}\left({\mathfrak{u}}_{2}\right)\right)}^{2}+\end{array}{\left({\mathcal{J}}_{{\Lambda }_{\widehat{{ \varsigma }_{1}}}}^{\ell}\left({\mathfrak{u}}_{2}\right)\right)}^{2}*{\left({\mathcal{J}}_{{\mathcal{G}}_{\widehat{{ \varsigma }_{1}}}}^{\ell}\left({\mathfrak{u}}_{2}\right)\right)}^{2}+{\left({\mathcal{J}}_{{\Lambda }_{\widehat{{ \varsigma }_{1}}}}^{\upsilon}\left({\mathfrak{u}}_{2}\right)\right)}^{2}*{\left({\mathcal{J}}_{{\mathcal{G}}_{\widehat{{ \varsigma }_{1}}}}^{\upsilon}\left({\mathfrak{u}}_{2}\right)\right)}^{2}\right)\right)+\\ {\gamma }_{2}\left({\Omega }_{2}\left(\begin{array}{c}{\left({\mathcal{T}}_{{\Lambda }_{\widehat{{ \varsigma }_{2}}}}^{\ell}\left({\mathfrak{u}}_{2}\right)\right)}^{2}*{\left({\mathcal{T}}_{{\mathcal{G}}_{\widehat{{ \varsigma }_{2}}}}^{\ell}\left({\mathfrak{u}}_{2}\right)\right)}^{2}+{\left({\mathcal{T}}_{{\Lambda }_{\widehat{{ \varsigma }_{2}}}}^{\upsilon}\left({\mathfrak{u}}_{2}\right)\right)}^{2}*{\left({\mathcal{T}}_{{\mathcal{G}}_{\widehat{{ \varsigma }_{2}}}}^{\upsilon}\left({\mathfrak{u}}_{2}\right)\right)}^{2}+\end{array}{\left({\mathcal{J}}_{{\Lambda }_{\widehat{{ \varsigma }_{2}}}}^{\ell}\left({\mathfrak{u}}_{2}\right)\right)}^{2}*{\left({\mathcal{J}}_{{\mathcal{G}}_{\widehat{{ \varsigma }_{2}}}}^{\ell}\left({\mathfrak{u}}_{2}\right)\right)}^{2}+{\left({\mathcal{J}}_{{\Lambda }_{\widehat{{ \varsigma }_{2}}}}^{\upsilon}\left({\mathfrak{u}}_{2}\right)\right)}^{2}*{\left({\mathcal{J}}_{{\mathcal{G}}_{\widehat{{ \varsigma }_{2}}}}^{\upsilon}\left({\mathfrak{u}}_{2}\right)\right)}^{2}\right)\right)+\\ \vdots \\ +\\ {\gamma }_{m}\left({\Omega }_{2}\left(\begin{array}{c}{\left({\mathcal{T}}_{{\Lambda }_{\widehat{{ \varsigma }_{m}}}}^{\ell}\left({\mathfrak{u}}_{2}\right)\right)}^{2}*{\left({\mathcal{T}}_{{\mathcal{G}}_{\widehat{{ \varsigma }_{m}}}}^{\ell}\left({\mathfrak{u}}_{2}\right)\right)}^{2}+{\left({\mathcal{T}}_{{\Lambda }_{\widehat{{ \varsigma }_{m}}}}^{\upsilon}\left({\mathfrak{u}}_{2}\right)\right)}^{2}*{\left({\mathcal{T}}_{{\mathcal{G}}_{\widehat{{ \varsigma }_{m}}}}^{\upsilon}\left({\mathfrak{u}}_{2}\right)\right)}^{2}+\end{array}{\left({\mathcal{J}}_{{\Lambda }_{\widehat{{ \varsigma }_{m}}}}^{\ell}\left({\mathfrak{u}}_{2}\right)\right)}^{2}*{\left({\mathcal{J}}_{{\mathcal{G}}_{\widehat{{ \varsigma }_{m}}}}^{\ell}\left({\mathfrak{u}}_{2}\right)\right)}^{2}+{\left({\mathcal{J}}_{{\Lambda }_{\widehat{{ \varsigma }_{m}}}}^{\upsilon}\left({\mathfrak{u}}_{2}\right)\right)}^{2}*{\left({\mathcal{J}}_{{\mathcal{G}}_{\widehat{{ \varsigma }_{m}}}}^{\upsilon}\left({\mathfrak{u}}_{2}\right)\right)}^{2}\right)\right)\end{array}\right\}$$

$$+$$

$$\vdots$$

$$+$$

$$\left\{\begin{array}{c}{\gamma }_{1}\left(\begin{array}{c}{\Omega }_{n}\left(\begin{array}{l}{\left({\mathcal{T}}_{{\Lambda }_{\widehat{{ \varsigma }_{1}}}}^{\ell}\left({\mathfrak{u}}_{n}\right)\right)}^{2}*{\left({\mathcal{T}}_{{\mathcal{G}}_{\widehat{{ \varsigma }_{1}}}}^{\ell}\left({\mathfrak{u}}_{n}\right)\right)}^{2}+{\left({\mathcal{T}}_{{\Lambda }_{\widehat{{ \varsigma }_{1}}}}^{\upsilon}\left({\mathfrak{u}}_{n}\right)\right)}^{2}*{\left({\mathcal{T}}_{{\mathcal{G}}_{\widehat{{ \varsigma }_{1}}}}^{\upsilon}\left({\mathfrak{u}}_{n}\right)\right)}^{2}\\ \quad +{\left({\mathcal{J}}_{{\Lambda }_{\widehat{{ \varsigma }_{1}}}}^{\ell}\left({\mathfrak{u}}_{n}\right)\right)}^{2}*{\left({\mathcal{J}}_{{\mathcal{G}}_{\widehat{{ \varsigma }_{1}}}}^{\ell}\left({\mathfrak{u}}_{n}\right)\right)}^{2}+{\left({\mathcal{J}}_{{\Lambda }_{\widehat{{ \varsigma }_{1}}}}^{\upsilon}\left({\mathfrak{u}}_{n}\right)\right)}^{2}*{\left({\mathcal{J}}_{{\mathcal{G}}_{\widehat{{ \varsigma }_{1}}}}^{\upsilon}\left({\mathfrak{u}}_{n}\right)\right)}^{2}\end{array}\right)\end{array}\right)+\\ {\gamma }_{2}\left({\Omega }_{n}\left(\begin{array}{l}{\left({\mathcal{T}}_{{\Lambda }_{\widehat{{ \varsigma }_{2}}}}^{\ell}\left({\mathfrak{u}}_{n}\right)\right)}^{2}*{\left({\mathcal{T}}_{{\mathcal{G}}_{\widehat{{ \varsigma }_{2}}}}^{\ell}\left({\mathfrak{u}}_{n}\right)\right)}^{2}+{\left({\mathcal{T}}_{{\Lambda }_{\widehat{{ \varsigma }_{2}}}}^{\upsilon}\left({\mathfrak{u}}_{n}\right)\right)}^{2}*{\left({\mathcal{T}}_{{\mathcal{G}}_{\widehat{{ \varsigma }_{2}}}}^{\upsilon}\left({\mathfrak{u}}_{n}\right)\right)}^{2}\\ \quad +{\left({\mathcal{J}}_{{\Lambda }_{\widehat{{ \varsigma }_{2}}}}^{\ell}\left({\mathfrak{u}}_{n}\right)\right)}^{2}*{\left({\mathcal{J}}_{{\mathcal{G}}_{\widehat{{ \varsigma }_{2}}}}^{\ell}\left({\mathfrak{u}}_{n}\right)\right)}^{2}+{\left({\mathcal{J}}_{{\Lambda }_{\widehat{{ \varsigma }_{2}}}}^{\upsilon}\left({\mathfrak{u}}_{n}\right)\right)}^{2}*{\left({\mathcal{J}}_{{\mathcal{G}}_{\widehat{{ \varsigma }_{2}}}}^{\upsilon}\left({\mathfrak{u}}_{n}\right)\right)}^{2}\end{array}\right)\right)+\\ \vdots \\ +\\ {\gamma }_{m}\left({\Omega }_{n}\begin{array}{c}\left(\begin{array}{l}{\left({\mathcal{T}}_{{\Lambda }_{\widehat{{ \varsigma }_{m}}}}^{\ell}\left({\mathfrak{u}}_{n}\right)\right)}^{2}*{\left({\mathcal{T}}_{{\mathcal{G}}_{\widehat{{ \varsigma }_{m}}}}^{\ell}\left({\mathfrak{u}}_{n}\right)\right)}^{2}+{\left({\mathcal{T}}_{{\Lambda }_{\widehat{{ \varsigma }_{m}}}}^{\upsilon}\left({\mathfrak{u}}_{n}\right)\right)}^{2}*{\left({\mathcal{T}}_{{\mathcal{G}}_{\widehat{{ \varsigma }_{m}}}}^{\upsilon}\left({\mathfrak{u}}_{n}\right)\right)}^{2}\\ \quad +{\left({\mathcal{J}}_{{\Lambda }_{\widehat{{ \varsigma }_{m}}}}^{\ell}\left({\mathfrak{u}}_{n}\right)\right)}^{2}*{\left({\mathcal{J}}_{{\mathcal{G}}_{\widehat{{ \varsigma }_{m}}}}^{\ell}\left({\mathfrak{u}}_{n}\right)\right)}^{2}+{\left({\mathcal{J}}_{{\Lambda }_{\widehat{{ \varsigma }_{m}}}}^{\upsilon}\left({\mathfrak{u}}_{n}\right)\right)}^{2}*{\left({\mathcal{J}}_{{\mathcal{G}}_{\widehat{{ \varsigma }_{m}}}}^{\upsilon}\left({\mathfrak{u}}_{n}\right)\right)}^{2}\end{array}\right)\end{array}\right)\end{array}\right\}$$

$$=\left\{\begin{array}{c}{\gamma }_{1}\left(\begin{array}{c}\sqrt{{\Omega }_{1}}{\left({\mathcal{T}}_{{\Lambda }_{\widehat{{ \varsigma }_{1}}}}^{\ell}\left({\mathfrak{u}}_{1}\right)\right)}^{2}*\sqrt{{\Omega }_{1}}{\left({\mathcal{T}}_{{\mathcal{G}}_{\widehat{{ \varsigma }_{1}}}}^{\ell}\left({\mathfrak{u}}_{1}\right)\right)}^{2}+\sqrt{{\Omega }_{1}}{\left({\mathcal{T}}_{{\Lambda }_{\widehat{{ \varsigma }_{1}}}}^{\upsilon}\left({\mathfrak{u}}_{1}\right)\right)}^{2}*\sqrt{{\Omega }_{1}}{\left({\mathcal{T}}_{{\mathcal{G}}_{\widehat{{ \varsigma }_{1}}}}^{\upsilon}\left({\mathfrak{u}}_{1}\right)\right)}^{2}+\\ \sqrt{{\Omega }_{1}}{\left({\mathcal{J}}_{{\Lambda }_{\widehat{{ \varsigma }_{1}}}}^{\ell}\left({\mathfrak{u}}_{1}\right)\right)}^{2}*\sqrt{{\Omega }_{1}}{\left({\mathcal{J}}_{{\mathcal{G}}_{\widehat{{ \varsigma }_{1}}}}^{\ell}\left({\mathfrak{u}}_{1}\right)\right)}^{2}+\sqrt{{\Omega }_{1}}{\left({\mathcal{J}}_{{\Lambda }_{\widehat{{ \varsigma }_{1}}}}^{\upsilon}\left({\mathfrak{u}}_{1}\right)\right)}^{2}*\sqrt{{\Omega }_{1}}{\left({\mathcal{J}}_{{\mathcal{G}}_{\widehat{{ \varsigma }_{1}}}}^{\upsilon}\left({\mathfrak{u}}_{1}\right)\right)}^{2}\end{array}\right)+\\ {\gamma }_{2}\left(\begin{array}{c}\sqrt{{\Omega }_{1}}{\left({\mathcal{T}}_{{\Lambda }_{\widehat{{ \varsigma }_{2}}}}^{\ell}\left({\mathfrak{u}}_{1}\right)\right)}^{2}*\sqrt{{\Omega }_{1}}{\left({\mathcal{T}}_{{\mathcal{G}}_{\widehat{{ \varsigma }_{2}}}}^{\ell}\left({\mathfrak{u}}_{1}\right)\right)}^{2}+\sqrt{{\Omega }_{1}}{\left({\mathcal{T}}_{{\Lambda }_{\widehat{{ \varsigma }_{2}}}}^{\upsilon}\left({\mathfrak{u}}_{1}\right)\right)}^{2}*\sqrt{{\Omega }_{1}}{\left({\mathcal{T}}_{{\mathcal{G}}_{\widehat{{ \varsigma }_{2}}}}^{\upsilon}\left({\mathfrak{u}}_{1}\right)\right)}^{2}+\\ \sqrt{{\Omega }_{1}}{\left({\mathcal{J}}_{{\Lambda }_{\widehat{{ \varsigma }_{2}}}}^{\ell}\left({\mathfrak{u}}_{1}\right)\right)}^{2}*\sqrt{{\Omega }_{1}}{\left({\mathcal{J}}_{{\mathcal{G}}_{\widehat{{ \varsigma }_{2}}}}^{\ell}\left({\mathfrak{u}}_{1}\right)\right)}^{2}+\sqrt{{\Omega }_{1}}{\left({\mathcal{J}}_{{\Lambda }_{\widehat{{ \varsigma }_{2}}}}^{\upsilon}\left({\mathfrak{u}}_{1}\right)\right)}^{2}*\sqrt{{\Omega }_{1}}{\left({\mathcal{J}}_{{\mathcal{G}}_{\widehat{{ \varsigma }_{2}}}}^{\upsilon}\left({\mathfrak{u}}_{1}\right)\right)}^{2}\end{array}\right)+\\ \vdots \\ +\\ {\gamma }_{m}\left(\begin{array}{c}\sqrt{{\Omega }_{1}}{\left({\mathcal{T}}_{{\Lambda }_{\widehat{{ \varsigma }_{m}}}}^{\ell}\left({\mathfrak{u}}_{1}\right)\right)}^{2}*\sqrt{{\Omega }_{1}}{\left({\mathcal{T}}_{{\mathcal{G}}_{\widehat{{ \varsigma }_{m}}}}^{\ell}\left({\mathfrak{u}}_{1}\right)\right)}^{2}+\sqrt{{\Omega }_{1}}{\left({\mathcal{T}}_{{\Lambda }_{\widehat{{ \varsigma }_{m}}}}^{\upsilon}\left({\mathfrak{u}}_{1}\right)\right)}^{2}*\sqrt{{\Omega }_{1}}{\left({\mathcal{T}}_{{\mathcal{G}}_{\widehat{{ \varsigma }_{m}}}}^{\upsilon}\left({\mathfrak{u}}_{1}\right)\right)}^{2}+\\ \sqrt{{\Omega }_{1}}{\left({\mathcal{J}}_{{\Lambda }_{\widehat{{ \varsigma }_{m}}}}^{\ell}\left({\mathfrak{u}}_{1}\right)\right)}^{2}*\sqrt{{\Omega }_{1}}{\left({\mathcal{J}}_{{\mathcal{G}}_{\widehat{{ \varsigma }_{m}}}}^{\ell}\left({\mathfrak{u}}_{1}\right)\right)}^{2}+\sqrt{{\Omega }_{1}}{\left({\mathcal{J}}_{{\Lambda }_{\widehat{{ \varsigma }_{m}}}}^{\upsilon}\left({\mathfrak{u}}_{1}\right)\right)}^{2}*\sqrt{{\Omega }_{1}}{\left({\mathcal{J}}_{{\mathcal{G}}_{\widehat{{ \varsigma }_{m}}}}^{\upsilon}\left({\mathfrak{u}}_{1}\right)\right)}^{2}\end{array}\right)\end{array}\right\}$$

$$+\left\{\begin{array}{c}{\gamma }_{1}\left(\begin{array}{c}\sqrt{{\Omega }_{2}}{\left({\mathcal{T}}_{{\Lambda }_{\widehat{{ \varsigma }_{1}}}}^{\ell}\left({\mathfrak{u}}_{2}\right)\right)}^{2}*\sqrt{{\Omega }_{2}}{\left({\mathcal{T}}_{{\mathcal{G}}_{\widehat{{ \varsigma }_{1}}}}^{\ell}\left({\mathfrak{u}}_{2}\right)\right)}^{2}+\sqrt{{\Omega }_{2}}{\left({\mathcal{T}}_{{\Lambda }_{\widehat{{ \varsigma }_{1}}}}^{\upsilon}\left({\mathfrak{u}}_{2}\right)\right)}^{2}*\sqrt{{\Omega }_{2}}{\left({\mathcal{T}}_{{\mathcal{G}}_{\widehat{{ \varsigma }_{2}}}}^{\upsilon}\left({\mathfrak{u}}_{2}\right)\right)}^{2}+\\ \sqrt{{\Omega }_{2}}{\left({\mathcal{J}}_{{\Lambda }_{\widehat{{ \varsigma }_{1}}}}^{\ell}\left({\mathfrak{u}}_{2}\right)\right)}^{2}*\sqrt{{\Omega }_{2}}{\left({\mathcal{J}}_{{\mathcal{G}}_{\widehat{{ \varsigma }_{1}}}}^{\ell}\left({\mathfrak{u}}_{2}\right)\right)}^{2}+\sqrt{{\Omega }_{2}}{\left({\mathcal{J}}_{{\Lambda }_{\widehat{{ \varsigma }_{1}}}}^{\upsilon}\left({\mathfrak{u}}_{2}\right)\right)}^{2}*\sqrt{{\Omega }_{2}}{\left({\mathcal{J}}_{{\mathcal{G}}_{\widehat{{ \varsigma }_{1}}}}^{\upsilon}\left({\mathfrak{u}}_{2}\right)\right)}^{2}\end{array}\right)+\\ {\gamma }_{2}\left(\begin{array}{c}\sqrt{{\Omega }_{2}}{\left({\mathcal{T}}_{{\Lambda }_{\widehat{{ \varsigma }_{2}}}}^{\ell}\left({\mathfrak{u}}_{2}\right)\right)}^{2}*\sqrt{{\Omega }_{2}}{\left({\mathcal{T}}_{{\mathcal{G}}_{\widehat{{ \varsigma }_{2}}}}^{\ell}\left({\mathfrak{u}}_{2}\right)\right)}^{2}+\sqrt{{\Omega }_{2}}{\left({\mathcal{T}}_{{\Lambda }_{\widehat{{ \varsigma }_{2}}}}^{\upsilon}\left({\mathfrak{u}}_{2}\right)\right)}^{2}*\sqrt{{\Omega }_{2}}{\left({\mathcal{T}}_{{\mathcal{G}}_{\widehat{{ \varsigma }_{2}}}}^{\upsilon}\left({\mathfrak{u}}_{2}\right)\right)}^{2}+\\ \sqrt{{\Omega }_{2}}{\left({\mathcal{J}}_{{\Lambda }_{\widehat{{ \varsigma }_{2}}}}^{\ell}\left({\mathfrak{u}}_{2}\right)\right)}^{2}*\sqrt{{\Omega }_{2}}{\left({\mathcal{J}}_{{\mathcal{G}}_{\widehat{{ \varsigma }_{2}}}}^{\ell}\left({\mathfrak{u}}_{2}\right)\right)}^{2}+\sqrt{{\Omega }_{2}}{\left({\mathcal{J}}_{{\Lambda }_{\widehat{{ \varsigma }_{2}}}}^{\upsilon}\left({\mathfrak{u}}_{2}\right)\right)}^{2}*\sqrt{{\Omega }_{2}}{\left({\mathcal{J}}_{{\mathcal{G}}_{\widehat{{ \varsigma }_{2}}}}^{\upsilon}\left({\mathfrak{u}}_{2}\right)\right)}^{2}\end{array}\right)+\\ \vdots \\ +\\ {\gamma }_{m}\left(\begin{array}{c}\sqrt{{\Omega }_{2}}{\left({\mathcal{T}}_{{\Lambda }_{\widehat{{ \varsigma }_{m}}}}^{\ell}\left({\mathfrak{u}}_{2}\right)\right)}^{2}*\sqrt{{\Omega }_{2}}{\left({\mathcal{T}}_{{\mathcal{G}}_{\widehat{{ \varsigma }_{m}}}}^{\ell}\left({\mathfrak{u}}_{2}\right)\right)}^{2}+\sqrt{{\Omega }_{2}}{\left({\mathcal{T}}_{{\Lambda }_{\widehat{{ \varsigma }_{m}}}}^{\upsilon}\left({\mathfrak{u}}_{2}\right)\right)}^{2}*\sqrt{{\Omega }_{2}}{\left({\mathcal{T}}_{{\mathcal{G}}_{\widehat{{ \varsigma }_{m}}}}^{\upsilon}\left({\mathfrak{u}}_{2}\right)\right)}^{2}+\\ \sqrt{{\Omega }_{2}}{\left({\mathcal{J}}_{{\Lambda }_{\widehat{{ \varsigma }_{m}}}}^{\ell}\left({\mathfrak{u}}_{2}\right)\right)}^{2}*\sqrt{{\Omega }_{2}}{\left({\mathcal{J}}_{{\mathcal{G}}_{\widehat{{ \varsigma }_{m}}}}^{\ell}\left({\mathfrak{u}}_{2}\right)\right)}^{2}+\sqrt{{\Omega }_{2}}{\left({\mathcal{J}}_{{\Lambda }_{\widehat{{ \varsigma }_{m}}}}^{\upsilon}\left({\mathfrak{u}}_{2}\right)\right)}^{2}*\sqrt{{\Omega }_{2}}{\left({\mathcal{J}}_{{\mathcal{G}}_{\widehat{{ \varsigma }_{m}}}}^{\upsilon}\left({\mathfrak{u}}_{2}\right)\right)}^{2}\end{array}\right)\end{array}\right\}$$

$$+$$

$$\vdots$$

$$+$$

$$\left\{\begin{array}{c}{\gamma }_{1}\left(\begin{array}{c}\sqrt{{\Omega }_{n}}{\left({\mathcal{T}}_{{\Lambda }_{\widehat{{ \varsigma }_{1}}}}^{\ell}\left({\mathfrak{u}}_{n}\right)\right)}^{2}*\sqrt{{\Omega }_{n}}{\left({\mathcal{T}}_{{\mathcal{G}}_{\widehat{{ \varsigma }_{1}}}}^{\ell}\left({\mathfrak{u}}_{n}\right)\right)}^{2}+\sqrt{{\Omega }_{n}}{\left({\mathcal{T}}_{{\Lambda }_{\widehat{{ \varsigma }_{1}}}}^{\upsilon}\left({\mathfrak{u}}_{n}\right)\right)}^{2}*\sqrt{{\Omega }_{n}}{\left({\mathcal{T}}_{{\mathcal{G}}_{\widehat{{ \varsigma }_{2}}}}^{\upsilon}\left({\mathfrak{u}}_{n}\right)\right)}^{2}+\\ \sqrt{{\Omega }_{n}}{\left({\mathcal{J}}_{{\Lambda }_{\widehat{{ \varsigma }_{1}}}}^{\ell}\left({\mathfrak{u}}_{n}\right)\right)}^{2}*\sqrt{{\Omega }_{n}}{\left({\mathcal{J}}_{{\mathcal{G}}_{\widehat{{ \varsigma }_{1}}}}^{\ell}\left({\mathfrak{u}}_{n}\right)\right)}^{2}+\sqrt{{\Omega }_{n}}{\left({\mathcal{J}}_{{\Lambda }_{\widehat{{ \varsigma }_{1}}}}^{\upsilon}\left({\mathfrak{u}}_{n}\right)\right)}^{2}*\sqrt{{\Omega }_{n}}{\left({\mathcal{J}}_{{\mathcal{G}}_{\widehat{{ \varsigma }_{1}}}}^{\upsilon}\left({\mathfrak{u}}_{n}\right)\right)}^{2}\end{array}\right)+\\ {\gamma }_{2}\left(\begin{array}{c}\sqrt{{\Omega }_{n}}{\left({\mathcal{T}}_{{\Lambda }_{\widehat{{ \varsigma }_{2}}}}^{\ell}\left({\mathfrak{u}}_{n}\right)\right)}^{2}*\sqrt{{\Omega }_{n}}{\left({\mathcal{T}}_{{\mathcal{G}}_{\widehat{{ \varsigma }_{2}}}}^{\ell}\left({\mathfrak{u}}_{n}\right)\right)}^{2}+\sqrt{{\Omega }_{n}}{\left({\mathcal{T}}_{{\Lambda }_{\widehat{{ \varsigma }_{2}}}}^{\upsilon}\left({\mathfrak{u}}_{n}\right)\right)}^{2}*\sqrt{{\Omega }_{n}}{\left({\mathcal{T}}_{{\mathcal{G}}_{\widehat{{ \varsigma }_{2}}}}^{\upsilon}\left({\mathfrak{u}}_{n}\right)\right)}^{2}+\\ \sqrt{{\Omega }_{n}}{\left({\mathcal{J}}_{{\Lambda }_{\widehat{{ \varsigma }_{2}}}}^{\ell}\left({\mathfrak{u}}_{n}\right)\right)}^{2}*\sqrt{{\Omega }_{n}}{\left({\mathcal{J}}_{{\mathcal{G}}_{\widehat{{ \varsigma }_{2}}}}^{\ell}\left({\mathfrak{u}}_{n}\right)\right)}^{2}+\sqrt{{\Omega }_{n}}{\left({\mathcal{J}}_{{\Lambda }_{\widehat{{ \varsigma }_{2}}}}^{\upsilon}\left({\mathfrak{u}}_{n}\right)\right)}^{2}*\sqrt{{\Omega }_{n}}{\left({\mathcal{J}}_{{\mathcal{G}}_{\widehat{{ \varsigma }_{2}}}}^{\upsilon}\left({\mathfrak{u}}_{n}\right)\right)}^{2}\end{array}\right)+\\ \vdots \\ +\\ {\gamma }_{m}\left(\begin{array}{c}\sqrt{{\Omega }_{n}}{\left({\mathcal{T}}_{{\Lambda }_{\widehat{{ \varsigma }_{m}}}}^{\ell}\left({\mathfrak{u}}_{n}\right)\right)}^{2}*\sqrt{{\Omega }_{n}}{\left({\mathcal{T}}_{{\mathcal{G}}_{\widehat{{ \varsigma }_{m}}}}^{\ell}\left({\mathfrak{u}}_{n}\right)\right)}^{2}+\sqrt{{\Omega }_{n}}{\left({\mathcal{T}}_{{\Lambda }_{\widehat{{ \varsigma }_{m}}}}^{\upsilon}\left({\mathfrak{u}}_{n}\right)\right)}^{2}*\sqrt{{\Omega }_{n}}{\left({\mathcal{T}}_{{\mathcal{G}}_{\widehat{{ \varsigma }_{m}}}}^{\upsilon}\left({\mathfrak{u}}_{n}\right)\right)}^{2}+\\ \sqrt{{\Omega }_{n}}{\left({\mathcal{J}}_{{\Lambda }_{\widehat{{ \varsigma }_{m}}}}^{\ell}\left({\mathfrak{u}}_{n}\right)\right)}^{2}*\sqrt{{\Omega }_{n}}{\left({\mathcal{J}}_{{\mathcal{G}}_{\widehat{{ \varsigma }_{m}}}}^{\ell}\left({\mathfrak{u}}_{n}\right)\right)}^{2}+\sqrt{{\Omega }_{n}}{\left({\mathcal{J}}_{{\Lambda }_{\widehat{{ \varsigma }_{m}}}}^{\upsilon}\left({\mathfrak{u}}_{n}\right)\right)}^{2}*\sqrt{{\Omega }_{n}}{\left({\mathcal{J}}_{{\mathcal{G}}_{\widehat{{ \varsigma }_{m}}}}^{\upsilon}\left({\mathfrak{u}}_{n}\right)\right)}^{2}\end{array}\right)\end{array}\right\}$$

$$=\left\{\begin{array}{c}\left(\begin{array}{c}\sqrt{{\gamma }_{1}}\sqrt{{\Omega }_{1}}{\left({\mathcal{T}}_{{\Lambda }_{\widehat{{ \varsigma }_{1}}}}^{\ell}\left({\mathfrak{u}}_{1}\right)\right)}^{2}*\sqrt{{\gamma }_{1}}\sqrt{{\Omega }_{1}}\left({\mathcal{T}}_{{\mathcal{G}}_{\widehat{{ \varsigma }_{1}}}}^{\ell}\left({\mathfrak{u}}_{1}\right)\right)+\sqrt{{\gamma }_{1}}\sqrt{{\Omega }_{1}}{\left({\mathcal{T}}_{{\Lambda }_{\widehat{{ \varsigma }_{1}}}}^{\upsilon}\left({\mathfrak{u}}_{1}\right)\right)}^{2}*\sqrt{{\gamma }_{1}}\sqrt{{\Omega }_{1}}{\left({\mathcal{T}}_{{\mathcal{G}}_{\widehat{{ \varsigma }_{1}}}}^{\upsilon}\left({\mathfrak{u}}_{1}\right)\right)}^{2}+\\ \sqrt{{\gamma }_{1}}\sqrt{{\Omega }_{1}}{\left({\mathcal{J}}_{{\Lambda }_{\widehat{{ \varsigma }_{1}}}}^{\ell}\left({\mathfrak{u}}_{1}\right)\right)}^{2}*\sqrt{{\gamma }_{1}}\sqrt{{\Omega }_{1}}\left({\mathcal{J}}_{{\mathcal{G}}_{\widehat{{ \varsigma }_{1}}}}^{\ell}\left({\mathfrak{u}}_{1}\right)\right)+\sqrt{{\gamma }_{1}}\sqrt{{\Omega }_{1}}{\left({\mathcal{J}}_{{\Lambda }_{\widehat{{ \varsigma }_{1}}}}^{\upsilon}\left({\mathfrak{u}}_{1}\right)\right)}^{2}*\sqrt{{\gamma }_{1}}\sqrt{{\Omega }_{1}}{\left({\mathcal{J}}_{{\mathcal{G}}_{\widehat{{ \varsigma }_{1}}}}^{\upsilon}\left({\mathfrak{u}}_{1}\right)\right)}^{2}\end{array}\right)+\\ \left(\begin{array}{c}\sqrt{{\gamma }_{2}}\sqrt{{\Omega }_{1}}{\left({\mathcal{T}}_{{\Lambda }_{\widehat{{ \varsigma }_{2}}}}^{\ell}\left({\mathfrak{u}}_{1}\right)\right)}^{2}*\sqrt{{\gamma }_{2}}\sqrt{{\Omega }_{1}}\left({\mathcal{T}}_{{\mathcal{G}}_{\widehat{{ \varsigma }_{2}}}}^{\ell}\left({\mathfrak{u}}_{1}\right)\right)+\sqrt{{\gamma }_{2}}\sqrt{{\Omega }_{1}}{\left({\mathcal{T}}_{{\Lambda }_{\widehat{{ \varsigma }_{2}}}}^{\upsilon}\left({\mathfrak{u}}_{1}\right)\right)}^{2}*\sqrt{{\gamma }_{2}}\sqrt{{\Omega }_{1}}{\left({\mathcal{T}}_{{\mathcal{G}}_{\widehat{{ \varsigma }_{2}}}}^{\upsilon}\left({\mathfrak{u}}_{1}\right)\right)}^{2}+\\ \sqrt{{\gamma }_{2}}\sqrt{{\Omega }_{1}}{\left({\mathcal{J}}_{{\Lambda }_{\widehat{{ \varsigma }_{2}}}}^{\ell}\left({\mathfrak{u}}_{1}\right)\right)}^{2}*\sqrt{{\gamma }_{2}}\sqrt{{\Omega }_{1}}\left({\mathcal{J}}_{{\mathcal{G}}_{\widehat{{ \varsigma }_{2}}}}^{\ell}\left({\mathfrak{u}}_{1}\right)\right)+\sqrt{{\gamma }_{2}}\sqrt{{\Omega }_{1}}{\left({\mathcal{J}}_{{\Lambda }_{\widehat{{ \varsigma }_{2}}}}^{\upsilon}\left({\mathfrak{u}}_{1}\right)\right)}^{2}*\sqrt{{\gamma }_{2}}\sqrt{{\Omega }_{1}}{\left({\mathcal{J}}_{{\mathcal{G}}_{\widehat{{ \varsigma }_{2}}}}^{\upsilon}\left({\mathfrak{u}}_{1}\right)\right)}^{2}\end{array}\right)+\\ \vdots \\ +\\ \begin{array}{c}\left(\begin{array}{c}\sqrt{{\gamma }_{m}}\sqrt{{\Omega }_{1}}{\left({\mathcal{T}}_{{\Lambda }_{\widehat{{ \varsigma }_{m}}}}^{\ell}\left({\mathfrak{u}}_{1}\right)\right)}^{2}*\sqrt{{\gamma }_{m}}\sqrt{{\Omega }_{1}}\left({\mathcal{T}}_{{\mathcal{G}}_{\widehat{{ \varsigma }_{m}}}}^{\ell}\left({\mathfrak{u}}_{1}\right)\right)+\sqrt{{\gamma }_{m}}\sqrt{{\Omega }_{1}}{\left({\mathcal{T}}_{{\Lambda }_{\widehat{{ \varsigma }_{m}}}}^{\upsilon}\left({\mathfrak{u}}_{1}\right)\right)}^{2}*\sqrt{{\gamma }_{m}}\sqrt{{\Omega }_{1}}{\left({\mathcal{T}}_{{\mathcal{G}}_{\widehat{{ \varsigma }_{m}}}}^{\upsilon}\left({\mathfrak{u}}_{1}\right)\right)}^{2}+\\ \sqrt{{\gamma }_{m}}\sqrt{{\Omega }_{1}}{\left({\mathcal{J}}_{{\Lambda }_{\widehat{{ \varsigma }_{m}}}}^{\ell}\left({\mathfrak{u}}_{1}\right)\right)}^{2}*\sqrt{{\gamma }_{m}}\sqrt{{\Omega }_{1}}\left({\mathcal{J}}_{{\mathcal{G}}_{\widehat{{ \varsigma }_{m}}}}^{\ell}\left({\mathfrak{u}}_{1}\right)\right)+\sqrt{{\gamma }_{m}}\sqrt{{\Omega }_{1}}{\left({\mathcal{J}}_{{\Lambda }_{\widehat{{ \varsigma }_{m}}}}^{\upsilon}\left({\mathfrak{u}}_{1}\right)\right)}^{2}*\sqrt{{\gamma }_{m}}\sqrt{{\Omega }_{1}}{\left({\mathcal{J}}_{{\mathcal{G}}_{\widehat{{ \varsigma }_{m}}}}^{\upsilon}\left({\mathfrak{u}}_{1}\right)\right)}^{2}\end{array}\right)\end{array}\end{array}\right\}$$

$$+\left\{\begin{array}{c}\left(\begin{array}{c}\sqrt{{\gamma }_{1}}\sqrt{{\Omega }_{2}}{\left({\mathcal{T}}_{{\Lambda }_{\widehat{{ \varsigma }_{1}}}}^{\ell}\left({\mathfrak{u}}_{2}\right)\right)}^{2}*\sqrt{{\gamma }_{1}}\sqrt{{\Omega }_{2}}\left({\mathcal{T}}_{{\mathcal{G}}_{\widehat{{ \varsigma }_{1}}}}^{\ell}\left({\mathfrak{u}}_{2}\right)\right)+\sqrt{{\gamma }_{1}}\sqrt{{\Omega }_{2}}{\left({\mathcal{T}}_{{\Lambda }_{\widehat{{ \varsigma }_{1}}}}^{\upsilon}\left({\mathfrak{u}}_{2}\right)\right)}^{2}*\sqrt{{\gamma }_{1}}\sqrt{{\Omega }_{2}}{\left({\mathcal{T}}_{{\mathcal{G}}_{\widehat{{ \varsigma }_{1}}}}^{\upsilon}\left({\mathfrak{u}}_{2}\right)\right)}^{2}+\\ \sqrt{{\gamma }_{1}}\sqrt{{\Omega }_{2}}{\left({\mathcal{J}}_{{\Lambda }_{\widehat{{ \varsigma }_{1}}}}^{\ell}\left({\mathfrak{u}}_{2}\right)\right)}^{2}*\sqrt{{\gamma }_{1}}\sqrt{{\Omega }_{2}}\left({\mathcal{J}}_{{\mathcal{G}}_{\widehat{{ \varsigma }_{1}}}}^{\ell}\left({\mathfrak{u}}_{2}\right)\right)+\sqrt{{\gamma }_{1}}\sqrt{{\Omega }_{2}}{\left({\mathcal{J}}_{{\Lambda }_{\widehat{{ \varsigma }_{1}}}}^{\upsilon}\left({\mathfrak{u}}_{2}\right)\right)}^{2}*\sqrt{{\gamma }_{1}}\sqrt{{\Omega }_{2}}{\left({\mathcal{J}}_{{\mathcal{G}}_{\widehat{{ \varsigma }_{1}}}}^{\upsilon}\left({\mathfrak{u}}_{2}\right)\right)}^{2}\end{array}\right)+\\ \left(\begin{array}{c}\sqrt{{\gamma }_{2}}\sqrt{{\Omega }_{2}}{\left({\mathcal{T}}_{{\Lambda }_{\widehat{{ \varsigma }_{2}}}}^{\ell}\left({\mathfrak{u}}_{2}\right)\right)}^{2}*\sqrt{{\gamma }_{2}}\sqrt{{\Omega }_{2}}\left({\mathcal{T}}_{{\mathcal{G}}_{\widehat{{ \varsigma }_{2}}}}^{\ell}\left({\mathfrak{u}}_{2}\right)\right)+\sqrt{{\gamma }_{2}}\sqrt{{\Omega }_{2}}{\left({\mathcal{T}}_{{\Lambda }_{\widehat{{ \varsigma }_{2}}}}^{\upsilon}\left({\mathfrak{u}}_{2}\right)\right)}^{2}*\sqrt{{\gamma }_{2}}\sqrt{{\Omega }_{2}}{\left({\mathcal{T}}_{{\mathcal{G}}_{\widehat{{ \varsigma }_{2}}}}^{\upsilon}\left({\mathfrak{u}}_{2}\right)\right)}^{2}+\\ \sqrt{{\gamma }_{2}}\sqrt{{\Omega }_{2}}{\left({\mathcal{J}}_{{\Lambda }_{\widehat{{ \varsigma }_{2}}}}^{\ell}\left({\mathfrak{u}}_{2}\right)\right)}^{2}*\sqrt{{\gamma }_{2}}\sqrt{{\Omega }_{2}}\left({\mathcal{J}}_{{\mathcal{G}}_{\widehat{{ \varsigma }_{2}}}}^{\ell}\left({\mathfrak{u}}_{2}\right)\right)+\sqrt{{\gamma }_{2}}\sqrt{{\Omega }_{2}}{\left({\mathcal{J}}_{{\Lambda }_{\widehat{{ \varsigma }_{2}}}}^{\upsilon}\left({\mathfrak{u}}_{2}\right)\right)}^{2}*\sqrt{{\gamma }_{2}}\sqrt{{\Omega }_{2}}{\left({\mathcal{J}}_{{\mathcal{G}}_{\widehat{{ \varsigma }_{2}}}}^{\upsilon}\left({\mathfrak{u}}_{2}\right)\right)}^{2}\end{array}\right)+\\ \vdots \\ +\\ \begin{array}{c}\left(\begin{array}{c}\sqrt{{\gamma }_{m}}\sqrt{{\Omega }_{2}}{\left({\mathcal{T}}_{{\Lambda }_{\widehat{{ \varsigma }_{m}}}}^{\ell}\left({\mathfrak{u}}_{2}\right)\right)}^{2}*\sqrt{{\gamma }_{m}}\sqrt{{\Omega }_{2}}\left({\mathcal{T}}_{{\mathcal{G}}_{\widehat{{ \varsigma }_{m}}}}^{\ell}\left({\mathfrak{u}}_{2}\right)\right)+\sqrt{{\gamma }_{m}}\sqrt{{\Omega }_{2}}{\left({\mathcal{T}}_{{\Lambda }_{\widehat{{ \varsigma }_{m}}}}^{\upsilon}\left({\mathfrak{u}}_{2}\right)\right)}^{2}*\sqrt{{\gamma }_{m}}\sqrt{{\Omega }_{2}}{\left({\mathcal{T}}_{{\mathcal{G}}_{\widehat{{ \varsigma }_{m}}}}^{\upsilon}\left({\mathfrak{u}}_{2}\right)\right)}^{2}+\\ \sqrt{{\gamma }_{m}}\sqrt{{\Omega }_{2}}{\left({\mathcal{J}}_{{\Lambda }_{\widehat{{ \varsigma }_{m}}}}^{\ell}\left({\mathfrak{u}}_{2}\right)\right)}^{2}*\sqrt{{\gamma }_{m}}\sqrt{{\Omega }_{2}}\left({\mathcal{J}}_{{\mathcal{G}}_{\widehat{{ \varsigma }_{m}}}}^{\ell}\left({\mathfrak{u}}_{2}\right)\right)+\sqrt{{\gamma }_{m}}\sqrt{{\Omega }_{2}}{\left({\mathcal{J}}_{{\Lambda }_{\widehat{{ \varsigma }_{m}}}}^{\upsilon}\left({\mathfrak{u}}_{2}\right)\right)}^{2}*\sqrt{{\gamma }_{m}}\sqrt{{\Omega }_{2}}{\left({\mathcal{J}}_{{\mathcal{G}}_{\widehat{{ \varsigma }_{m}}}}^{\upsilon}\left({\mathfrak{u}}_{2}\right)\right)}^{2}\end{array}\right)\end{array}\end{array}\right\}$$

$$+$$

$$\vdots$$

$$+$$

$$\left\{\begin{array}{c}\left(\begin{array}{c}\sqrt{{\gamma }_{1}}\sqrt{{\Omega }_{n}}{\left({\mathcal{T}}_{{\Lambda }_{\widehat{{ \varsigma }_{1}}}}^{\ell}\left({\mathfrak{u}}_{n}\right)\right)}^{2}*\sqrt{{\gamma }_{1}}\sqrt{{\Omega }_{n}}\left({\mathcal{T}}_{{\mathcal{G}}_{\widehat{{ \varsigma }_{1}}}}^{\ell}\left({\mathfrak{u}}_{n}\right)\right)+\sqrt{{\gamma }_{1}}\sqrt{{\Omega }_{n}}{\left({\mathcal{T}}_{{\Lambda }_{\widehat{{ \varsigma }_{1}}}}^{\upsilon}\left({\mathfrak{u}}_{n}\right)\right)}^{2}*\sqrt{{\gamma }_{1}}\sqrt{{\Omega }_{n}}{\left({\mathcal{T}}_{{\mathcal{G}}_{\widehat{{ \varsigma }_{1}}}}^{\upsilon}\left({\mathfrak{u}}_{n}\right)\right)}^{2}+\\ \sqrt{{\gamma }_{1}}\sqrt{{\Omega }_{n}}{\left({\mathcal{J}}_{{\Lambda }_{\widehat{{ \varsigma }_{1}}}}^{\ell}\left({\mathfrak{u}}_{n}\right)\right)}^{2}*\sqrt{{\gamma }_{1}}\sqrt{{\Omega }_{n}}\left({\mathcal{J}}_{{\mathcal{G}}_{\widehat{{ \varsigma }_{1}}}}^{\ell}\left({\mathfrak{u}}_{n}\right)\right)+\sqrt{{\gamma }_{1}}\sqrt{{\Omega }_{n}}{\left({\mathcal{J}}_{{\Lambda }_{\widehat{{ \varsigma }_{1}}}}^{\upsilon}\left({\mathfrak{u}}_{n}\right)\right)}^{2}*\sqrt{{\gamma }_{1}}\sqrt{{\Omega }_{n}}{\left({\mathcal{J}}_{{\mathcal{G}}_{\widehat{{ \varsigma }_{1}}}}^{\upsilon}\left({\mathfrak{u}}_{n}\right)\right)}^{2}\end{array}\right)+\\ \left(\begin{array}{c}\sqrt{{\gamma }_{2}}\sqrt{{\Omega }_{n}}{\left({\mathcal{T}}_{{\Lambda }_{\widehat{{ \varsigma }_{2}}}}^{\ell}\left({\mathfrak{u}}_{n}\right)\right)}^{2}*\sqrt{{\gamma }_{2}}\sqrt{{\Omega }_{n}}\left({\mathcal{T}}_{{\mathcal{G}}_{\widehat{{ \varsigma }_{2}}}}^{\ell}\left({\mathfrak{u}}_{n}\right)\right)+\sqrt{{\gamma }_{2}}\sqrt{{\Omega }_{n}}{\left({\mathcal{T}}_{{\Lambda }_{\widehat{{ \varsigma }_{2}}}}^{\upsilon}\left({\mathfrak{u}}_{n}\right)\right)}^{2}*\sqrt{{\gamma }_{2}}\sqrt{{\Omega }_{n}}{\left({\mathcal{T}}_{{\mathcal{G}}_{\widehat{{ \varsigma }_{2}}}}^{\upsilon}\left({\mathfrak{u}}_{n}\right)\right)}^{2}+\\ \sqrt{{\gamma }_{2}}\sqrt{{\Omega }_{n}}{\left({\mathcal{J}}_{{\Lambda }_{\widehat{{ \varsigma }_{2}}}}^{\ell}\left({\mathfrak{u}}_{n}\right)\right)}^{2}*\sqrt{{\gamma }_{2}}\sqrt{{\Omega }_{n}}\left({\mathcal{J}}_{{\mathcal{G}}_{\widehat{{ \varsigma }_{2}}}}^{\ell}\left({\mathfrak{u}}_{n}\right)\right)+\sqrt{{\gamma }_{2}}\sqrt{{\Omega }_{n}}{\left({\mathcal{J}}_{{\Lambda }_{\widehat{{ \varsigma }_{2}}}}^{\upsilon}\left({\mathfrak{u}}_{n}\right)\right)}^{2}*\sqrt{{\gamma }_{2}}\sqrt{{\Omega }_{n}}{\left({\mathcal{J}}_{{\mathcal{G}}_{\widehat{{ \varsigma }_{2}}}}^{\upsilon}\left({\mathfrak{u}}_{n}\right)\right)}^{2}\end{array}\right)+\\ \vdots \\ +\\ \begin{array}{c}\left(\begin{array}{c}\sqrt{{\gamma }_{m}}\sqrt{{\Omega }_{n}}{\left({\mathcal{T}}_{{\Lambda }_{\widehat{{ \varsigma }_{m}}}}^{\ell}\left({\mathfrak{u}}_{n}\right)\right)}^{2}*\sqrt{{\gamma }_{m}}\sqrt{{\Omega }_{n}}\left({\mathcal{T}}_{{\mathcal{G}}_{\widehat{{ \varsigma }_{m}}}}^{\ell}\left({\mathfrak{u}}_{n}\right)\right)+\sqrt{{\gamma }_{m}}\sqrt{{\Omega }_{n}}{\left({\mathcal{T}}_{{\Lambda }_{\widehat{{ \varsigma }_{m}}}}^{\upsilon}\left({\mathfrak{u}}_{n}\right)\right)}^{2}*\sqrt{{\gamma }_{m}}\sqrt{{\Omega }_{n}}{\left({\mathcal{T}}_{{\mathcal{G}}_{\widehat{{ \varsigma }_{m}}}}^{\upsilon}\left({\mathfrak{u}}_{n}\right)\right)}^{2}+\\ \sqrt{{\gamma }_{m}}\sqrt{{\Omega }_{n}}{\left({\mathcal{J}}_{{\Lambda }_{\widehat{{ \varsigma }_{m}}}}^{\ell}\left({\mathfrak{u}}_{n}\right)\right)}^{2}*\sqrt{{\gamma }_{m}}\sqrt{{\Omega }_{n}}\left({\mathcal{J}}_{{\mathcal{G}}_{\widehat{{ \varsigma }_{m}}}}^{\ell}\left({\mathfrak{u}}_{n}\right)\right)+\sqrt{{\gamma }_{m}}\sqrt{{\Omega }_{n}}{\left({\mathcal{J}}_{{\Lambda }_{\widehat{{ \varsigma }_{m}}}}^{\upsilon}\left({\mathfrak{u}}_{n}\right)\right)}^{2}*\sqrt{{\gamma }_{m}}\sqrt{{\Omega }_{n}}{\left({\mathcal{J}}_{{\mathcal{G}}_{\widehat{{ \varsigma }_{m}}}}^{\upsilon}\left({\mathfrak{u}}_{n}\right)\right)}^{2}\end{array}\right)\end{array}\end{array}\right\}$$

Employing the Cauchy Schwarz inequality

$$\begin{aligned} & {\mathcal{C}}_{WIVPFHSS}{\left(\left(\Lambda ,\mathcal{A}\right), \left(\mathcal{G},\mathcal{B}\right)\right)}^{2} \\ & \quad \le \left\{\begin{array}{c}\left(\begin{array}{c}{\gamma }_{1}{\Omega }_{1}\left\{{\left({\mathcal{T}}_{{\Lambda }_{\widehat{{ \varsigma }_{1}}}}^{\ell}\left({\mathfrak{u}}_{1}\right)\right)}^{4}+{\left({\mathcal{T}}_{{\Lambda }_{\widehat{{ \varsigma }_{1}}}}^{\upsilon}\left({\mathfrak{u}}_{1}\right)\right)}^{4}+{\left({\mathcal{J}}_{{\Lambda }_{\widehat{{ \varsigma }_{1}}}}^{\ell}\left({\mathfrak{u}}_{1}\right)\right)}^{4}+{\left({\mathcal{J}}_{{\Lambda }_{\widehat{{ \varsigma }_{1}}}}^{\upsilon}\left({\mathfrak{u}}_{1}\right)\right)}^{4}\right\}+\\ {\gamma }_{2}{\Omega }_{1}\left\{{\left({\mathcal{T}}_{{\Lambda }_{\widehat{{ \varsigma }_{2}}}}^{\ell}\left({\mathfrak{u}}_{1}\right)\right)}^{4}+{\left({\mathcal{T}}_{{\Lambda }_{\widehat{{ \varsigma }_{2}}}}^{\upsilon}\left({\mathfrak{u}}_{1}\right)\right)}^{4}+{\left({\mathcal{J}}_{{\Lambda }_{\widehat{{ \varsigma }_{2}}}}^{\ell}\left({\mathfrak{u}}_{1}\right)\right)}^{4}+{\left({\mathcal{J}}_{{\Lambda }_{\widehat{{ \varsigma }_{2}}}}^{\upsilon}\left({\mathfrak{u}}_{1}\right)\right)}^{4}\right\}+\\ \vdots \\ +\\ {\gamma }_{m}{\Omega }_{1}\left\{{\left({\mathcal{T}}_{{\Lambda }_{\widehat{{ \varsigma }_{m}}}}^{\ell}\left({\mathfrak{u}}_{1}\right)\right)}^{4}+{\left({\mathcal{T}}_{{\Lambda }_{\widehat{{ \varsigma }_{m}}}}^{\upsilon}\left({\mathfrak{u}}_{1}\right)\right)}^{4}+{\left({\mathcal{J}}_{{\Lambda }_{\widehat{{ \varsigma }_{m}}}}^{\ell}\left({\mathfrak{u}}_{1}\right)\right)}^{4}+{\left({\mathcal{J}}_{{\Lambda }_{\widehat{{ \varsigma }_{m}}}}^{\upsilon}\left({\mathfrak{u}}_{1}\right)\right)}^{4}\right\}\end{array}\right)+\\ \left(\begin{array}{c}{\gamma }_{1}{\Omega }_{2}\left\{{\left({\mathcal{T}}_{{\Lambda }_{\widehat{{ \varsigma }_{1}}}}^{\ell}\left({\mathfrak{u}}_{2}\right)\right)}^{4}+{\left({\mathcal{T}}_{{\Lambda }_{\widehat{{ \varsigma }_{1}}}}^{\upsilon}\left({\mathfrak{u}}_{2}\right)\right)}^{4}+{\left({\mathcal{J}}_{{\Lambda }_{\widehat{{ \varsigma }_{1}}}}^{\ell}\left({\mathfrak{u}}_{2}\right)\right)}^{4}+{\left({\mathcal{J}}_{{\Lambda }_{\widehat{{ \varsigma }_{1}}}}^{\upsilon}\left({\mathfrak{u}}_{2}\right)\right)}^{4}\right\}+\\ {\gamma }_{2}{\Omega }_{2}\left\{{\left({\mathcal{T}}_{{\Lambda }_{\widehat{{ \varsigma }_{2}}}}^{\ell}\left({\mathfrak{u}}_{2}\right)\right)}^{4}+{\left({\mathcal{T}}_{{\Lambda }_{\widehat{{ \varsigma }_{2}}}}^{\upsilon}\left({\mathfrak{u}}_{2}\right)\right)}^{4}+{\left({\mathcal{J}}_{{\Lambda }_{\widehat{{ \varsigma }_{2}}}}^{\ell}\left({\mathfrak{u}}_{2}\right)\right)}^{4}+{\left({\mathcal{J}}_{{\Lambda }_{\widehat{{ \varsigma }_{2}}}}^{\upsilon}\left({\mathfrak{u}}_{2}\right)\right)}^{4}\right\}+\\ \vdots \\ +\\ {\gamma }_{m}{\Omega }_{2}\left\{{\left({\mathcal{T}}_{{\Lambda }_{\widehat{{ \varsigma }_{m}}}}^{\ell}\left({\mathfrak{u}}_{2}\right)\right)}^{4}+{\left({\mathcal{T}}_{{\Lambda }_{\widehat{{ \varsigma }_{m}}}}^{\upsilon}\left({\mathfrak{u}}_{2}\right)\right)}^{4}+{\left({\mathcal{J}}_{{\Lambda }_{\widehat{{ \varsigma }_{m}}}}^{\ell}\left({\mathfrak{u}}_{2}\right)\right)}^{4}+{\left({\mathcal{J}}_{{\Lambda }_{\widehat{{ \varsigma }_{m}}}}^{\upsilon}\left({\mathfrak{u}}_{2}\right)\right)}^{4}\right\}\end{array}\right)+\\ \vdots \\ +\\ \begin{array}{c}\left(\begin{array}{c}{\gamma }_{1}{\Omega }_{n}\left\{{\left({\mathcal{T}}_{{\Lambda }_{\widehat{{ \varsigma }_{1}}}}^{\ell}\left({\mathfrak{u}}_{n}\right)\right)}^{4}+{\left({\mathcal{T}}_{{\Lambda }_{\widehat{{ \varsigma }_{1}}}}^{\upsilon}\left({\mathfrak{u}}_{n}\right)\right)}^{4}+{\left({\mathcal{J}}_{{\Lambda }_{\widehat{{ \varsigma }_{1}}}}^{\ell}\left({\mathfrak{u}}_{n}\right)\right)}^{4}+{\left({\mathcal{J}}_{{\Lambda }_{\widehat{{ \varsigma }_{1}}}}^{\upsilon}\left({\mathfrak{u}}_{n}\right)\right)}^{4}\right\}+\\ {\gamma }_{2}{\Omega }_{n}\left\{{\left({\mathcal{T}}_{{\Lambda }_{\widehat{{ \varsigma }_{2}}}}^{\ell}\left({\mathfrak{u}}_{n}\right)\right)}^{4}+{\left({\mathcal{T}}_{{\Lambda }_{\widehat{{ \varsigma }_{2}}}}^{\upsilon}\left({\mathfrak{u}}_{n}\right)\right)}^{4}+{\left({\mathcal{J}}_{{\Lambda }_{\widehat{{ \varsigma }_{2}}}}^{\ell}\left({\mathfrak{u}}_{n}\right)\right)}^{4}+{\left({\mathcal{J}}_{{\Lambda }_{\widehat{{ \varsigma }_{2}}}}^{\upsilon}\left({\mathfrak{u}}_{n}\right)\right)}^{4}\right\}+\\ \vdots \\ +\\ {\gamma }_{m}{\Omega }_{n}\left\{{\left({\mathcal{T}}_{{\Lambda }_{\widehat{{ \varsigma }_{m}}}}^{\ell}\left({\mathfrak{u}}_{n}\right)\right)}^{4}+{\left({\mathcal{T}}_{{\Lambda }_{\widehat{{ \varsigma }_{m}}}}^{\upsilon}\left({\mathfrak{u}}_{n}\right)\right)}^{4}+{\left({\mathcal{J}}_{{\Lambda }_{\widehat{{ \varsigma }_{m}}}}^{\ell}\left({\mathfrak{u}}_{n}\right)\right)}^{4}+{\left({\mathcal{J}}_{{\Lambda }_{\widehat{{ \varsigma }_{m}}}}^{\upsilon}\left({\mathfrak{u}}_{n}\right)\right)}^{4}\right\}\end{array}\right)\end{array}\end{array}\right\} \\ & \quad \times \left\{\begin{array}{c}\left(\begin{array}{c}{\gamma }_{1}{\Omega }_{1}\left\{{\left({\mathcal{T}}_{{\mathcal{G}}_{\widehat{{ \varsigma }_{1}}}}^{\ell}\left({\mathfrak{u}}_{1}\right)\right)}^{4}+{\left({\mathcal{T}}_{{\mathcal{G}}_{\widehat{{ \varsigma }_{1}}}}^{\upsilon}\left({\mathfrak{u}}_{1}\right)\right)}^{4}+{\left({\mathcal{J}}_{{\mathcal{G}}_{\widehat{{ \varsigma }_{1}}}}^{\ell}\left({\mathfrak{u}}_{1}\right)\right)}^{4}+{\left({\mathcal{J}}_{{\mathcal{G}}_{\widehat{{ \varsigma }_{1}}}}^{\upsilon}\left({\mathfrak{u}}_{1}\right)\right)}^{4}\right\}+\\ {\gamma }_{2}{\Omega }_{1}\left\{{\left({\mathcal{T}}_{{\mathcal{G}}_{\widehat{{ \varsigma }_{2}}}}^{\ell}\left({\mathfrak{u}}_{1}\right)\right)}^{4}+{\left({\mathcal{T}}_{{\mathcal{G}}_{\widehat{{ \varsigma }_{2}}}}^{\upsilon}\left({\mathfrak{u}}_{1}\right)\right)}^{4}+{\left({\mathcal{J}}_{{\mathcal{G}}_{\widehat{{ \varsigma }_{2}}}}^{\ell}\left({\mathfrak{u}}_{1}\right)\right)}^{4}+{\left({\mathcal{J}}_{{\mathcal{G}}_{\widehat{{ \varsigma }_{2}}}}^{\upsilon}\left({\mathfrak{u}}_{1}\right)\right)}^{4}\right\}+\\ \vdots \\ +\\ {\gamma }_{m}{\Omega }_{1}\left\{{\left({\mathcal{T}}_{{\mathcal{G}}_{\widehat{{ \varsigma }_{m}}}}^{\ell}\left({\mathfrak{u}}_{1}\right)\right)}^{4}+{\left({\mathcal{T}}_{{\mathcal{G}}_{\widehat{{ \varsigma }_{m}}}}^{\upsilon}\left({\mathfrak{u}}_{1}\right)\right)}^{4}+{\left({\mathcal{J}}_{{\mathcal{G}}_{\widehat{{ \varsigma }_{m}}}}^{\ell}\left({\mathfrak{u}}_{1}\right)\right)}^{4}+{\left({\mathcal{J}}_{{\mathcal{G}}_{\widehat{{ \varsigma }_{m}}}}^{\upsilon}\left({\mathfrak{u}}_{1}\right)\right)}^{4}\right\}\end{array}\right)+\\ \left(\begin{array}{c}{\gamma }_{1}{\Omega }_{2}\left\{{\left({\mathcal{T}}_{{\mathcal{G}}_{\widehat{{ \varsigma }_{1}}}}^{\ell}\left({\mathfrak{u}}_{2}\right)\right)}^{4}+{\left({\mathcal{T}}_{{\mathcal{G}}_{\widehat{{ \varsigma }_{1}}}}^{\upsilon}\left({\mathfrak{u}}_{2}\right)\right)}^{4}+{\left({\mathcal{J}}_{{\mathcal{G}}_{\widehat{{ \varsigma }_{1}}}}^{\ell}\left({\mathfrak{u}}_{2}\right)\right)}^{4}+{\left({\mathcal{J}}_{{\mathcal{G}}_{\widehat{{ \varsigma }_{1}}}}^{\upsilon}\left({\mathfrak{u}}_{2}\right)\right)}^{4}\right\}+\\ {\gamma }_{2}{\Omega }_{2}\left\{{\left({\mathcal{T}}_{{\mathcal{G}}_{\widehat{{ \varsigma }_{2}}}}^{\ell}\left({\mathfrak{u}}_{2}\right)\right)}^{4}+{\left({\mathcal{T}}_{{\mathcal{G}}_{\widehat{{ \varsigma }_{2}}}}^{\upsilon}\left({\mathfrak{u}}_{2}\right)\right)}^{4}+{\left({\mathcal{J}}_{{\mathcal{G}}_{\widehat{{ \varsigma }_{2}}}}^{\ell}\left({\mathfrak{u}}_{2}\right)\right)}^{4}+{\left({\mathcal{J}}_{{\mathcal{G}}_{\widehat{{ \varsigma }_{2}}}}^{\upsilon}\left({\mathfrak{u}}_{2}\right)\right)}^{4}\right\}+\\ \vdots \\ +\\ {\gamma }_{m}{\Omega }_{2}\left\{{\left({\mathcal{T}}_{{\mathcal{G}}_{\widehat{{ \varsigma }_{m}}}}^{\ell}\left({\mathfrak{u}}_{2}\right)\right)}^{4}+{\left({\mathcal{T}}_{{\mathcal{G}}_{\widehat{{ \varsigma }_{m}}}}^{\upsilon}\left({\mathfrak{u}}_{2}\right)\right)}^{4}+{\left({\mathcal{J}}_{{\mathcal{G}}_{\widehat{{ \varsigma }_{m}}}}^{\ell}\left({\mathfrak{u}}_{2}\right)\right)}^{4}+{\left({\mathcal{J}}_{{\mathcal{G}}_{\widehat{{ \varsigma }_{m}}}}^{\upsilon}\left({\mathfrak{u}}_{2}\right)\right)}^{4}\right\}\end{array}\right)+\\ \vdots \\ +\\ \begin{array}{c}\left(\begin{array}{c}{\gamma }_{1}{\Omega }_{n}\left\{{\left({\mathcal{T}}_{{\mathcal{G}}_{\widehat{{ \varsigma }_{1}}}}^{\ell}\left({\mathfrak{u}}_{n}\right)\right)}^{4}+{\left({\mathcal{T}}_{{\mathcal{G}}_{\widehat{{ \varsigma }_{1}}}}^{\upsilon}\left({\mathfrak{u}}_{n}\right)\right)}^{4}+{\left({\mathcal{J}}_{{\mathcal{G}}_{\widehat{{ \varsigma }_{1}}}}^{\ell}\left({\mathfrak{u}}_{n}\right)\right)}^{4}+{\left({\mathcal{J}}_{{\mathcal{G}}_{\widehat{{ \varsigma }_{1}}}}^{\upsilon}\left({\mathfrak{u}}_{n}\right)\right)}^{4}\right\}+\\ {\gamma }_{2}{\Omega }_{n}\left\{{\left({\mathcal{T}}_{{\mathcal{G}}_{\widehat{{ \varsigma }_{2}}}}^{\ell}\left({\mathfrak{u}}_{n}\right)\right)}^{4}+{\left({\mathcal{T}}_{{\mathcal{G}}_{\widehat{{ \varsigma }_{2}}}}^{\upsilon}\left({\mathfrak{u}}_{n}\right)\right)}^{4}+{\left({\mathcal{J}}_{{\mathcal{G}}_{\widehat{{ \varsigma }_{2}}}}^{\ell}\left({\mathfrak{u}}_{n}\right)\right)}^{4}+{\left({\mathcal{J}}_{{\mathcal{G}}_{\widehat{{ \varsigma }_{2}}}}^{\upsilon}\left({\mathfrak{u}}_{n}\right)\right)}^{4}\right\}+\\ \vdots \\ +\\ {\gamma }_{m}{\Omega }_{n}\left\{{\left({\mathcal{T}}_{{\mathcal{G}}_{\widehat{{ \varsigma }_{m}}}}^{\ell}\left({\mathfrak{u}}_{n}\right)\right)}^{4}+{\left({\mathcal{T}}_{{\mathcal{G}}_{\widehat{{ \varsigma }_{m}}}}^{\upsilon}\left({\mathfrak{u}}_{n}\right)\right)}^{4}+{\left({\mathcal{J}}_{{\mathcal{G}}_{\widehat{{ \varsigma }_{m}}}}^{\ell}\left({\mathfrak{u}}_{n}\right)\right)}^{4}+{\left({\mathcal{J}}_{{\mathcal{G}}_{\widehat{{ \varsigma}_{m}}}}^{\upsilon}\left({\mathfrak{u}}_{n}\right)\right)}^{4}\right\}\end{array}\right)\end{array}\end{array}\right\}\end{aligned}$$

$$\begin{aligned} {\mathcal{C}}_{{IVPFHSS}} & \left( {\left( {\Lambda ,{\mathcal{A}}} \right),\left( {{\mathcal{G}},{~\mathcal{B}}} \right)} \right)^{2} \\ & \le \mathop \sum \limits_{{k = 1}}^{m} \mathop \sum \limits_{{i = 1}}^{n} \left\{ {\left( {\left( {{\mathcal{T}}_{{\Lambda _{{\widehat{{\varsigma _{k} }}}} }}^{\ell } \left( {{\mathfrak{u}}_{i} } \right)} \right)^{4} + \left( {{\mathcal{T}}_{{\Lambda _{{\widehat{{\varsigma _{k} }}}} }}^{\upsilon } \left( {{\mathfrak{u}}_{i} } \right)} \right)^{4} } \right) + \left( {\left( {{\mathcal{J}}_{{\Lambda _{{\widehat{{\varsigma _{k} }}}} }}^{\ell } \left( {{\mathfrak{u}}_{i} } \right)} \right)^{4} + \left( {{\mathcal{J}}_{{\Lambda _{{\widehat{{\varsigma _{k} }}}} }}^{\upsilon } \left( {{\mathfrak{u}}_{i} } \right)} \right)^{4} } \right)} \right\} \\ & \;\;\; \times \mathop \sum \limits_{{k = 1}}^{m} \mathop \sum \limits_{{i = 1}}^{n} \left\{ {\left( {\left( {{\mathcal{T}}_{{{\mathcal{G}}_{{\widehat{{\varsigma _{k} }}}} }}^{\ell } \left( {{\mathfrak{u}}_{i} } \right)} \right)^{4} + \left( {{\mathcal{T}}_{{{\mathcal{G}}_{{\widehat{{\varsigma _{k} }}}} }}^{\upsilon } \left( {{\mathfrak{u}}_{i} } \right)} \right)^{4} } \right) + \left( {\left( {{\mathcal{J}}_{{{\mathcal{G}}_{{\widehat{{\varsigma _{k} }}}} }}^{\ell } \left( {{\mathfrak{u}}_{i} } \right)} \right)^{4} + \left( {{\mathcal{J}}_{{{\mathcal{G}}_{{\widehat{{\varsigma _{k} }}}} }}^{\upsilon } \left( {{\mathfrak{u}}_{i} } \right)} \right)^{4} } \right)} \right\} \\ \end{aligned}$$

$${\mathcal{C}}_{WIVPFHSS}{\left(\left(\Lambda ,\mathcal{A}\right), \left(\mathcal{G},\mathcal{B}\right)\right)}^{2}\le {\mathcal{E}}_{WIVPFHSS}\left(\Lambda ,\mathcal{A}\right)\times {\mathcal{E}}_{WIVPFHSS}\left(\mathcal{G},\mathcal{B}\right)$$

Using Definition 11, we get

$${\mathbb{C}}_{WIVPFHSS}\left(\left(\Lambda ,\mathcal{A}\right), \left(\mathcal{G},\mathcal{B}\right)\right)\le 1$$

So, it is verified that $0\le {\mathbb{C}}_{WIVPFHSS}\left(\left(\Lambda ,\mathcal{A}\right), \left(\mathcal{G},\mathcal{B}\right)\right)\le 1$.

### Proof 2.

As $\left(\Lambda ,\mathcal{A}\right)=\left\{\left({\mathfrak{u}}_{i}, \left(\left[{\mathcal{T}}_{{\Lambda }_{\widehat{{ \varsigma }_{k}}}}^{\ell}\left({\mathfrak{u}}_{i}\right), {\mathcal{T}}_{{\Lambda }_{\widehat{{ \varsigma }_{k}}}}^{\upsilon}\left({\mathfrak{u}}_{i}\right)\right], \left[{\mathcal{J}}_{{\Lambda }_{\widehat{{ \varsigma }_{k}}}}^{\ell}\left({\mathfrak{u}}_{i}\right), {\mathcal{J}}_{{\Lambda }_{\widehat{{ \varsigma }_{k}}}}^{\upsilon}\left({\mathfrak{u}}_{i}\right)\right]\right)\right) \left. {} \right| {\mathfrak{u}}_{i}\in \mathsf{U}\right\}$ and $\left(\mathcal{G},\mathcal{B}\right)=\left\{\left({\mathfrak{u}}_{i}, \left(\left[{\mathcal{T}}_{{\mathcal{G}}_{\widehat{{ \varsigma }_{k}}}}^{\ell}\left({\mathfrak{u}}_{i}\right), {\mathcal{T}}_{{\mathcal{G}}_{\widehat{{ \varsigma }_{k}}}}^{\upsilon}\left({\mathfrak{u}}_{i}\right)\right], \left[{\mathcal{J}}_{{\mathcal{G}}_{\widehat{{ \varsigma }_{k}}}}^{\ell}\left({\mathfrak{u}}_{i}\right), {\mathcal{J}}_{{\mathcal{G}}_{\widehat{{ \varsigma }_{k}}}}^{\upsilon}\left({\mathfrak{u}}_{i}\right)\right]\right)\right) \left. {} \right| {\mathfrak{u}}_{i}\in \mathsf{U}\right\}$ be two IVPFHSS. From Eq. (9)

$$\begin{aligned} & {\mathbb{C}}_{WIVPFHSS}\left(\left(\Lambda ,\mathcal{A}\right), \left(\mathcal{G},\mathcal{B}\right)\right)\\ & \quad =\frac{\sum_{k=1}^{m}{\gamma }_{k}\left(\sum_{i=1}^{n}{\Omega }_{i}\left(\begin{array}{l}{\left({\mathcal{T}}_{{\Lambda }_{\widehat{{ \varsigma }_{k}}}}^{\ell}\left({\mathfrak{u}}_{i}\right)\right)}^{2}*{\left({\mathcal{T}}_{{\mathcal{G}}_{\widehat{{ \varsigma }_{k}}}}^{\ell}\left({\mathfrak{u}}_{i}\right)\right)}^{2}+{\left({\mathcal{T}}_{{\Lambda }_{\widehat{{ \varsigma }_{k}}}}^{\upsilon}\left({\mathfrak{u}}_{i}\right)\right)}^{2}*{\left({\mathcal{T}}_{{\mathcal{G}}_{\widehat{{ \varsigma }_{k}}}}^{\upsilon}\left({\mathfrak{u}}_{i}\right)\right)}^{2}\\ \quad +{\left({\mathcal{J}}_{{\Lambda }_{\widehat{{ \varsigma }_{k}}}}^{\ell}\left({\mathfrak{u}}_{i}\right)\right)}^{2}*{\left({\mathcal{J}}_{{\mathcal{G}}_{\widehat{{ \varsigma }_{k}}}}^{\ell}\left({\mathfrak{u}}_{i}\right)\right)}^{2}+{\left({\mathcal{J}}_{{\Lambda }_{\widehat{{ \varsigma }_{k}}}}^{\upsilon}\left({\mathfrak{u}}_{i}\right)\right)}^{2}*{\left({\mathcal{J}}_{{\mathcal{G}}_{\widehat{{ \varsigma }_{k}}}}^{\upsilon}\left({\mathfrak{u}}_{i}\right)\right)}^{2}\end{array}\right)\right)}{\begin{array}{l}\sqrt{\sum_{k=1}^{m}{\gamma }_{k}\left(\sum_{i=1}^{n}{\Omega }_{i}\left({\left({\mathcal{T}}_{{\Lambda }_{\widehat{{ \varsigma }_{k}}}}^{\ell}\left({\mathfrak{u}}_{i}\right)\right)}^{4}+{\left({\mathcal{T}}_{{\Lambda }_{\widehat{{ \varsigma }_{k}}}}^{\upsilon}\left({\mathfrak{u}}_{i}\right)\right)}^{4}+{\left({\mathcal{J}}_{{\Lambda }_{\widehat{{ \varsigma }_{k}}}}^{\ell}\left({\mathfrak{u}}_{i}\right)\right)}^{4}+{\left({\mathcal{J}}_{{\Lambda }_{\widehat{{ \varsigma }_{k}}}}^{\upsilon}\left({\mathfrak{u}}_{i}\right)\right)}^{4}\right)\right)} \\ \quad \sqrt{\sum_{k=1}^{m}{\gamma }_{k}\left(\sum_{i=1}^{n}{\Omega }_{i}\left({\left({\mathcal{T}}_{{\mathcal{G}}_{\widehat{{ \varsigma }_{k}}}}^{\ell}\left({\mathfrak{u}}_{i}\right)\right)}^{4}+{\left({\mathcal{T}}_{{\mathcal{G}}_{\widehat{{ \varsigma }_{k}}}}^{\upsilon}\left({\mathfrak{u}}_{i}\right)\right)}^{4}+{\left({\mathcal{J}}_{{\mathcal{G}}_{\widehat{{ \varsigma }_{k}}}}^{\ell}\left({\mathfrak{u}}_{i}\right)\right)}^{4}+{\left({\mathcal{J}}_{{\mathcal{G}}_{\widehat{{ \varsigma }_{k}}}}^{\upsilon}\left({\mathfrak{u}}_{i}\right)\right)}^{4}\right)\right)}\end{array}} \end{aligned}$$

$$=\frac{\sum_{k=1}^{m}{\gamma }_{k}\left(\sum_{i=1}^{n}{\Omega }_{i}\left(\begin{array}{l}{\left({\mathcal{T}}_{{\mathcal{G}}_{\widehat{{ \varsigma }_{k}}}}^{\ell}\left({\mathfrak{u}}_{i}\right)\right)}^{2}*{\left({\mathcal{T}}_{{\Lambda }_{\widehat{{ \varsigma }_{k}}}}^{\ell}\left({\mathfrak{u}}_{i}\right)\right)}^{2}+{\left({\mathcal{T}}_{{\mathcal{G}}_{\widehat{{ \varsigma }_{k}}}}^{\upsilon}\left({\mathfrak{u}}_{i}\right)\right)}^{2}*{\left({\mathcal{T}}_{{\Lambda }_{\widehat{{ \varsigma }_{k}}}}^{\upsilon}\left({\mathfrak{u}}_{i}\right)\right)}^{2}\\ \quad +{\left({\mathcal{J}}_{{\mathcal{G}}_{\widehat{{ \varsigma }_{k}}}}^{\ell}\left({\mathfrak{u}}_{i}\right)\right)}^{2}*{\left({\mathcal{J}}_{{\Lambda }_{\widehat{{ \varsigma }_{k}}}}^{\ell}\left({\mathfrak{u}}_{i}\right)\right)}^{2}+{\left({\mathcal{J}}_{{\mathcal{G}}_{\widehat{{ \varsigma }_{k}}}}^{\upsilon}\left({\mathfrak{u}}_{i}\right)\right)}^{2}*{\left({\mathcal{J}}_{{\Lambda }_{\widehat{{ \varsigma }_{k}}}}^{\upsilon}\left({\mathfrak{u}}_{i}\right)\right)}^{2}\end{array}\right)\right)}{\begin{array}{l}\sqrt{\sum_{k=1}^{m}{\gamma }_{k}\left(\sum_{i=1}^{n}{\Omega }_{i}\left({\left({\mathcal{T}}_{{\mathcal{G}}_{\widehat{{ \varsigma }_{k}}}}^{\ell}\left({\mathfrak{u}}_{i}\right)\right)}^{4}+{\left({\mathcal{T}}_{{\mathcal{G}}_{\widehat{{ \varsigma }_{k}}}}^{\upsilon}\left({\mathfrak{u}}_{i}\right)\right)}^{4}+{\left({\mathcal{J}}_{{\mathcal{G}}_{\widehat{{ \varsigma }_{k}}}}^{\ell}\left({\mathfrak{u}}_{i}\right)\right)}^{4}+{\left({\mathcal{J}}_{{\mathcal{G}}_{\widehat{{ \varsigma }_{k}}}}^{\upsilon}\left({\mathfrak{u}}_{i}\right)\right)}^{4}\right)\right)}\\ \quad \sqrt{\sum_{k=1}^{m}{\gamma }_{k}\left(\sum_{i=1}^{n}{\Omega }_{i}\left({\left({\mathcal{T}}_{{\Lambda }_{\widehat{{ \varsigma }_{k}}}}^{\ell}\left({\mathfrak{u}}_{i}\right)\right)}^{4}+{\left({\mathcal{T}}_{{\Lambda }_{\widehat{{ \varsigma }_{k}}}}^{\upsilon}\left({\mathfrak{u}}_{i}\right)\right)}^{4}+{\left({\mathcal{J}}_{{\Lambda }_{\widehat{{ \varsigma }_{k}}}}^{\ell}\left({\mathfrak{u}}_{i}\right)\right)}^{4}+{\left({\mathcal{J}}_{{\Lambda }_{\widehat{{ \varsigma }_{k}}}}^{\upsilon}\left({\mathfrak{u}}_{i}\right)\right)}^{4}\right)\right)}\end{array}}$$

$$={\mathbb{C}}_{WIVPFHSS}\left( \left(\mathcal{G},\mathcal{B}\right), \left(\Lambda ,\mathcal{A}\right)\right)$$

### Proof 3.

As

$$\begin{aligned} & {\mathbb{C}}_{WIVPFHSS}\left(\left(\Lambda ,\mathcal{A}\right), \left(\mathcal{G},\mathcal{B}\right)\right)\\ &\quad=\frac{\sum_{k=1}^{m}{\gamma }_{k}\left(\sum_{i=1}^{n}{\Omega }_{i}\left(\begin{array}{c}{\left({\mathcal{T}}_{{\Lambda }_{\widehat{{ \varsigma }_{k}}}}^{\ell}\left({\mathfrak{u}}_{i}\right)\right)}^{2}*{\left({\mathcal{T}}_{{\mathcal{G}}_{\widehat{{ \varsigma }_{k}}}}^{\ell}\left({\mathfrak{u}}_{i}\right)\right)}^{2}+{\left({\mathcal{T}}_{{\Lambda }_{\widehat{{ \varsigma }_{k}}}}^{\upsilon}\left({\mathfrak{u}}_{i}\right)\right)}^{2}*{\left({\mathcal{T}}_{{\mathcal{G}}_{\widehat{{ \varsigma }_{k}}}}^{\upsilon}\left({\mathfrak{u}}_{i}\right)\right)}^{2}+\\ {\left({\mathcal{J}}_{{\Lambda }_{\widehat{{ \varsigma }_{k}}}}^{\ell}\left({\mathfrak{u}}_{i}\right)\right)}^{2}*{\left({\mathcal{J}}_{{\mathcal{G}}_{\widehat{{ \varsigma }_{k}}}}^{\ell}\left({\mathfrak{u}}_{i}\right)\right)}^{2}+{\left({\mathcal{J}}_{{\Lambda }_{\widehat{{ \varsigma }_{k}}}}^{\upsilon}\left({\mathfrak{u}}_{i}\right)\right)}^{2}*{\left({\mathcal{J}}_{{\mathcal{G}}_{\widehat{{ \varsigma }_{k}}}}^{\upsilon}\left({\mathfrak{u}}_{i}\right)\right)}^{2}\end{array}\right)\right) }{\left(\begin{array}{c}\sqrt{\sum_{k=1}^{m}{\gamma }_{k}\left(\sum_{i=1}^{n}{\Omega }_{i}\left({\left({\mathcal{T}}_{{\Lambda }_{\widehat{{ \varsigma }_{k}}}}^{\ell}\left({\mathfrak{u}}_{i}\right)\right)}^{4}+{\left({\mathcal{T}}_{{\Lambda }_{\widehat{{ \varsigma }_{k}}}}^{\upsilon}\left({\mathfrak{u}}_{i}\right)\right)}^{4}+{\left({\mathcal{J}}_{{\Lambda }_{\widehat{{ \varsigma }_{k}}}}^{\ell}\left({\mathfrak{u}}_{i}\right)\right)}^{4}+{\left({\mathcal{J}}_{{\Lambda }_{\widehat{{ \varsigma }_{k}}}}^{\upsilon}\left({\mathfrak{u}}_{i}\right)\right)}^{4}\right)\right)}\\ \sqrt{\sum_{k=1}^{m}{\gamma }_{k}\left(\sum_{i=1}^{n}{\Omega }_{i}\left({\left({\mathcal{T}}_{{\mathcal{G}}_{\widehat{{ \varsigma }_{k}}}}^{\ell}\left({\mathfrak{u}}_{i}\right)\right)}^{4}+{\left({\mathcal{T}}_{{\mathcal{G}}_{\widehat{{ \varsigma }_{k}}}}^{\upsilon}\left({\mathfrak{u}}_{i}\right)\right)}^{4}+{\left({\mathcal{J}}_{{\mathcal{G}}_{\widehat{{ \varsigma }_{k}}}}^{\ell}\left({\mathfrak{u}}_{i}\right)\right)}^{4}+{\left({\mathcal{J}}_{{\mathcal{G}}_{\widehat{{ \varsigma }_{k}}}}^{\upsilon}\left({\mathfrak{u}}_{i}\right)\right)}^{4}\right)\right)}\end{array}\right)}\end{aligned}$$

As

$${\mathcal{T}}_{{\Lambda _{{\widehat{{\varsigma _{k} }}}} }}^{\ell } \left( {{\mathfrak{u}}_{i} } \right) = {\mathcal{T}}_{{{\mathcal{G}}_{{\widehat{{\varsigma _{k} }}}} }}^{\ell } \left( {{\mathfrak{u}}_{i} } \right),\;\;{\mathcal{T}}_{{\Lambda _{{\widehat{{\varsigma _{k} }}}} }}^{\upsilon } \left( {{\mathfrak{u}}_{i} } \right) = {\mathcal{T}}_{{{\mathcal{G}}_{{\widehat{{\varsigma _{k} }}}} }}^{\upsilon } \left( {{\mathfrak{u}}_{i} } \right),\;\;{\mathcal{J}}_{{\Lambda _{{\widehat{{\varsigma _{k} }}}} }}^{\ell } \left( {{\mathfrak{u}}_{i} } \right) = {\mathcal{J}}_{{{\mathcal{G}}_{{\widehat{{\varsigma _{k} }}}} }}^{\ell } \left( {{\mathfrak{u}}_{i} } \right)\;\;{\text{and}}\;{\mathcal{J}}_{{\Lambda _{{\widehat{{\varsigma _{k} }}}} }}^{\upsilon } \left( {{\mathfrak{u}}_{i} } \right) = {\mathcal{J}}_{{{\mathcal{G}}_{{\widehat{{\varsigma _{k} }}}} }}^{\upsilon } \left( {{\mathfrak{u}}_{i} } \right).\;\;{\text{So,}}$$

$$\begin{aligned} & {\mathbb{C}}_{WIVPFHSS}\left(\left(\Lambda ,\mathcal{A}\right), \left(\mathcal{G},\mathcal{B}\right)\right)\\ &\quad=\frac{\sum_{k=1}^{m}{\gamma }_{k}\left(\sum_{i=1}^{n}{\Omega }_{i}\left\{\left({\left({\mathcal{T}}_{{\Lambda }_{\widehat{{ \varsigma }_{k}}}}^{\ell}\left({\mathfrak{u}}_{i}\right)\right)}^{4}+{\left({\mathcal{T}}_{{\Lambda }_{\widehat{{ \varsigma }_{k}}}}^{\upsilon}\left({\mathfrak{u}}_{i}\right)\right)}^{4}\right)+\left({\left({\mathcal{J}}_{{\Lambda }_{\widehat{{ \varsigma }_{k}}}}^{\ell}\left({\mathfrak{u}}_{i}\right)\right)}^{4}+{\left({\mathcal{J}}_{{\Lambda }_{\widehat{{ \varsigma }_{k}}}}^{\upsilon}\left({\mathfrak{u}}_{i}\right)\right)}^{4}\right)\right\}\right) }{\left(\begin{array}{c}\sqrt{\sum_{k=1}^{m}{\gamma }_{k}\left(\sum_{i=1}^{n}{\Omega }_{i}\left\{\left({\left({\mathcal{T}}_{{\Lambda }_{\widehat{{ \varsigma }_{k}}}}^{\ell}\left({\mathfrak{u}}_{i}\right)\right)}^{4}+{\left({\mathcal{T}}_{{\Lambda }_{\widehat{{ \varsigma }_{k}}}}^{\upsilon}\left({\mathfrak{u}}_{i}\right)\right)}^{4}\right)+\left({\left({\mathcal{J}}_{{\Lambda }_{\widehat{{ \varsigma }_{k}}}}^{\ell}\left({\mathfrak{u}}_{i}\right)\right)}^{4}+{\left({\mathcal{J}}_{{\Lambda }_{\widehat{{ \varsigma }_{k}}}}^{\upsilon}\left({\mathfrak{u}}_{i}\right)\right)}^{4}\right)\right\}\right)} \\ \sqrt{\sum_{k=1}^{m}{\gamma }_{k}\left(\sum_{i=1}^{n}{\Omega }_{i}\left\{\left({\left({\mathcal{T}}_{{\Lambda }_{\widehat{{ \varsigma }_{k}}}}^{\ell}\left({\mathfrak{u}}_{i}\right)\right)}^{4}+{\left({\mathcal{T}}_{{\Lambda }_{\widehat{{ \varsigma }_{k}}}}^{\upsilon}\left({\mathfrak{u}}_{i}\right)\right)}^{4}\right)+\left({\left({\mathcal{J}}_{{\Lambda }_{\widehat{{ \varsigma }_{k}}}}^{\ell}\left({\mathfrak{u}}_{i}\right)\right)}^{4}+{\left({\mathcal{J}}_{{\Lambda }_{\widehat{{ \varsigma }_{k}}}}^{\upsilon}\left({\mathfrak{u}}_{i}\right)\right)}^{4}\right)\right\}\right)}\end{array}\right)}\end{aligned}$$

$${\mathbb{C}}_{WIVPFHSS}\left(\left(\Lambda ,\mathcal{A}\right), \left(\mathcal{G},\mathcal{B}\right)\right)=1$$

### Definition 20

Let $\left(\Lambda ,\mathcal{A}\right)=\left\{\left({\mathfrak{u}}_{i}, \left(\left[{\mathcal{T}}_{{\Lambda }_{\widehat{{ \varsigma }_{k}}}}^{\ell}\left({\mathfrak{u}}_{i}\right), {\mathcal{T}}_{{\Lambda }_{\widehat{{ \varsigma }_{k}}}}^{\upsilon}\left({\mathfrak{u}}_{i}\right)\right], \left[{\mathcal{J}}_{{\Lambda }_{\widehat{{ \varsigma }_{k}}}}^{\ell}\left({\mathfrak{u}}_{i}\right), {\mathcal{J}}_{{\Lambda }_{\widehat{{ \varsigma }_{k}}}}^{\upsilon}\left({\mathfrak{u}}_{i}\right)\right]\right)\right) \left. {} \right| {\mathfrak{u}}_{i}\in \mathsf{U}\right\}$ and $\left(\mathcal{G},\mathcal{B}\right)=\left\{\left({\mathfrak{u}}_{i}, \left(\left[{\mathcal{T}}_{{\mathcal{G}}_{\widehat{{ \varsigma }_{k}}}}^{\ell}\left({\mathfrak{u}}_{i}\right), {\mathcal{T}}_{{\mathcal{G}}_{\widehat{{ \varsigma }_{k}}}}^{\upsilon}\left({\mathfrak{u}}_{i}\right)\right], \left[{\mathcal{J}}_{{\mathcal{G}}_{\widehat{{ \varsigma }_{k}}}}^{\ell}\left({\mathfrak{u}}_{i}\right), {\mathcal{J}}_{{\mathcal{G}}_{\widehat{{ \varsigma }_{k}}}}^{\upsilon}\left({\mathfrak{u}}_{i}\right)\right]\right)\right) \left. {} \right| {\mathfrak{u}}_{i}\in \mathsf{U}\right\}$ be two IVPFHSS. Then, the WCC between them is also defined as:

10 $$\begin{aligned} & {\mathbb{C}}_{WIVPFHSS}^{1}\left(\left(\Lambda ,\mathcal{A}\right), \left(\mathcal{G},\mathcal{B}\right)\right)\\ & \quad =\frac{{\mathcal{C}}_{WIVPFHSS}\left(\left(\Lambda ,\mathcal{A}\right), \left(\mathcal{G},\mathcal{B}\right)\right)}{max\left\{{\mathcal{E}}_{WIVPFHSS}\left(\Lambda ,\mathcal{A}\right), {\mathcal{E}}_{WIVPFHSS}\left(\mathcal{G},\mathcal{B}\right)\right\}}\\ & \quad =\frac{\sum_{k=1}^{m}{\gamma }_{k}\left(\sum_{i=1}^{n}{\Omega }_{i}\left(\begin{array}{c}{\left({\mathcal{T}}_{{\Lambda }_{\widehat{{ \varsigma }_{k}}}}^{\ell}\left({\mathfrak{u}}_{i}\right)\right)}^{2}*{\left({\mathcal{T}}_{{\mathcal{G}}_{\widehat{{ \varsigma }_{k}}}}^{\ell}\left({\mathfrak{u}}_{i}\right)\right)}^{2}+{\left({\mathcal{T}}_{{\Lambda }_{\widehat{{ \varsigma }_{k}}}}^{\upsilon}\left({\mathfrak{u}}_{i}\right)\right)}^{2}*{\left({\mathcal{T}}_{{\mathcal{G}}_{\widehat{{ \varsigma }_{k}}}}^{\upsilon}\left({\mathfrak{u}}_{i}\right)\right)}^{2}\\ \quad +{\left({\mathcal{J}}_{{\Lambda }_{\widehat{{ \varsigma }_{k}}}}^{\ell}\left({\mathfrak{u}}_{i}\right)\right)}^{2}*{\left({\mathcal{J}}_{{\mathcal{G}}_{\widehat{{ \varsigma }_{k}}}}^{\ell}\left({\mathfrak{u}}_{i}\right)\right)}^{2}+{\left({\mathcal{J}}_{{\Lambda }_{\widehat{{ \varsigma }_{k}}}}^{\upsilon}\left({\mathfrak{u}}_{i}\right)\right)}^{2}*{\left({\mathcal{J}}_{{\mathcal{G}}_{\widehat{{ \varsigma }_{k}}}}^{\upsilon}\left({\mathfrak{u}}_{i}\right)\right)}^{2}\end{array}\right)\right)}{max\left\{\begin{array}{c}\sum_{k=1}^{m}{\gamma }_{k}\left(\sum_{i=1}^{n}{\Omega }_{i}\left({\left({\mathcal{T}}_{{\Lambda }_{\widehat{{ \varsigma }_{k}}}}^{\ell}\left({\mathfrak{u}}_{i}\right)\right)}^{4}+{\left({\mathcal{T}}_{{\Lambda }_{\widehat{{ \varsigma }_{k}}}}^{\upsilon}\left({\mathfrak{u}}_{i}\right)\right)}^{4}+{\left({\mathcal{J}}_{{\Lambda }_{\widehat{{ \varsigma }_{k}}}}^{\ell}\left({\mathfrak{u}}_{i}\right)\right)}^{4}+{\left({\mathcal{J}}_{{\Lambda }_{\widehat{{ \varsigma }_{k}}}}^{\upsilon}\left({\mathfrak{u}}_{i}\right)\right)}^{4}\right)\right), \\ \sum_{k=1}^{m}{\gamma }_{k}\left(\sum_{i=1}^{n}{\Omega }_{i}\left({\left({\mathcal{T}}_{{\mathcal{G}}_{\widehat{{ \varsigma }_{k}}}}^{\ell}\left({\mathfrak{u}}_{i}\right)\right)}^{4}+{\left({\mathcal{T}}_{{\mathcal{G}}_{\widehat{{ \varsigma }_{k}}}}^{\upsilon}\left({\mathfrak{u}}_{i}\right)\right)}^{4}+{\left({\mathcal{J}}_{{\mathcal{G}}_{\widehat{{ \varsigma }_{k}}}}^{\ell}\left({\mathfrak{u}}_{i}\right)\right)}^{4}+{\left({\mathcal{J}}_{{\mathcal{G}}_{\widehat{{ \varsigma }_{k}}}}^{\upsilon}\left({\mathfrak{u}}_{i}\right)\right)}^{4}\right)\right)\end{array}\right\}}\end{aligned}$$

### Theorem 21

Let $\left( {\Lambda ,{\mathcal{A}}} \right) = \left\{ {\left. {\left( {{\mathfrak{u}}_{i},\left( {\left[ {{\mathcal{T}}_{{\Lambda_{{\widehat{{\varsigma_{k} }}}} }}^{\ell } \left( {{\mathfrak{u}}_{i} } \right),{ }{\mathcal{T}}_{{\Lambda_{{\widehat{{\varsigma_{k} }}}} }}^{\upsilon } \left( {{\mathfrak{u}}_{i} } \right)} \right], \left[ {{\mathcal{J}}_{{\Lambda_{{\widehat{{\varsigma_{k} }}}} }}^{\ell } \left( {{\mathfrak{u}}_{i} } \right),{ }{\mathcal{J}}_{{\Lambda_{{\widehat{{\varsigma_{k} }}}} }}^{\upsilon } \left( {{\mathfrak{u}}_{i} } \right)} \right]} \right)} \right) } \right|{\mathfrak{u}}_{i} \in {U}} \right\}$ and $\left( {{\mathcal{G}},{ \mathcal{B}}} \right) = \left\{ {\left. {\left( {{\mathfrak{u}}_{i},\left( {\left[ {{\mathcal{T}}_{{{\mathcal{G}}_{{\widehat{{\varsigma_{k} }}}} }}^{\ell } \left( {{\mathfrak{u}}_{i} } \right),{ }{\mathcal{T}}_{{{\mathcal{G}}_{{\widehat{{\varsigma_{k} }}}} }}^{\upsilon } \left( {{\mathfrak{u}}_{i} } \right)} \right], \left[ {{\mathcal{J}}_{{{\mathcal{G}}_{{\widehat{{\varsigma_{k} }}}} }}^{\ell } \left( {{\mathfrak{u}}_{i} } \right),{ }{\mathcal{J}}_{{{\mathcal{G}}_{{\widehat{{\varsigma_{k} }}}} }}^{\upsilon } \left( {{\mathfrak{u}}_{i} } \right)} \right]} \right)} \right)} \right| {\mathfrak{u}}_{i} \in {U}} \right\}$ beItwo IVIFHSS. Then,

1. $$0 \le {\mathbb{C}}_{WIVPFHSS}^{1} \left( {\left( {\Lambda ,{\mathcal{A}}} \right),\left( {{\mathcal{G}},{ \mathcal{B}}} \right)} \right) \le 1.$$
2. $${\mathbb{C}}_{WIVPFHSS}^{1} \left( {\left( {\Lambda ,{\mathcal{A}}} \right),\left( {{\mathcal{G}},{ \mathcal{B}}} \right)} \right) = {\mathbb{C}}_{WIVPFHSS}^{1} \left( {\left( {{\mathcal{G}},{ \mathcal{B}}} \right),\left( {\Lambda ,{\mathcal{A}}} \right)} \right)$$
3. If $\left( {\Lambda ,{\mathcal{A}}} \right) = \left( {{\mathcal{G}},{ \mathcal{B}}} \right)$, i.e., $\forall$ $i$, $j$, ${\mathcal{T}}_{{\Lambda_{{\widehat{{\varsigma_{k} }}}} }}^{\ell } \left( {{\mathfrak{u}}_{i} } \right) = {\mathcal{T}}_{{{\mathcal{G}}_{{\widehat{{\varsigma_{k} }}}} }}^{\ell } \left( {{\mathfrak{u}}_{i} } \right)$, ${\mathcal{T}}_{{\Lambda_{{\widehat{{\varsigma_{k} }}}} }}^{\upsilon } \left( {{\mathfrak{u}}_{i} } \right) = {\mathcal{T}}_{{{\mathcal{G}}_{{\widehat{{\varsigma_{k} }}}} }}^{\upsilon } \left( {{\mathfrak{u}}_{i} } \right)$, ${\mathcal{J}}_{{\Lambda_{{\widehat{{\varsigma_{k} }}}} }}^{\ell } \left( {{\mathfrak{u}}_{i} } \right) = {\mathcal{J}}_{{{\mathcal{G}}_{{\widehat{{\varsigma_{k} }}}} }}^{\ell } \left( {{\mathfrak{u}}_{i} } \right)$, and ${\mathcal{J}}_{{\Lambda_{{\widehat{{\varsigma_{k} }}}} }}^{\upsilon } \left( {{\mathfrak{u}}_{i} } \right) = {\mathcal{J}}_{{{\mathcal{G}}_{{\widehat{{\varsigma_{k} }}}} }}^{\upsilon } \left( {{\mathfrak{u}}_{i} } \right)$, then ${\mathbb{C}}_{WIVPFHSS}^{1} \left( {\left( {\Lambda ,{\mathcal{A}}} \right),{ }\left( {{\mathcal{G}},{ \mathcal{B}}} \right)} \right) = 1$.

### Proof.

The proof of case 2 is very easy and straightforward, and case 3 is like Theorem 12, which involves case 3 as well. Also, it’s apparent that ${\mathbb{C}}_{WIVPFHSS}^{1} \left( {\left( {\Lambda ,{\mathcal{A}}} \right),{ }\left( {{\mathcal{G}},{ \mathcal{B}}} \right)} \right) \ge 0$ in case 1. To complete the proof, we only need to show that ${\mathbb{C}}_{WIVPFHSS}^{1} \left( {\left( {\Lambda ,{\mathcal{A}}} \right),{ }\left( {{\mathcal{G}},{ \mathcal{B}}} \right)} \right) \le 1$. This can be shown by using the inequality ${\mathcal{C}}_{WIVPFHSS} \left( {\left( {\Lambda ,{\mathcal{A}}} \right),{ }\left( {{\mathcal{G}},{ \mathcal{B}}} \right)} \right)^{2} \le {\mathcal{E}}_{WIVPFHSS} \left( {\Lambda ,{\mathcal{A}}} \right) \times {\mathcal{E}}_{WIVPFHSS} \left( {{\mathcal{G}},{ \mathcal{B}}} \right)$. Therefore, ${\mathcal{C}}_{WIVPFHSS} \left( {\left( {\Lambda ,{\mathcal{A}}} \right),\left( {{\mathcal{G}},{ \mathcal{B}}} \right)} \right) \le max\left\{ {{\mathcal{E}}_{WIVPFHSS} \left( {\Lambda ,{\mathcal{A}}} \right), {\mathcal{E}}_{WIVPFHSS} \left( {{\mathcal{G}},{ \mathcal{B}}} \right)} \right\}$. Hence, ${\mathbb{C}}_{WIVPFHSS}^{1} \left( {\left( {\Lambda ,{\mathcal{A}}} \right),\left( {{\mathcal{G}},{ \mathcal{B}}} \right)} \right) \le 1$.

## Proposed TOPSIS approach based on correlation coefficient to resolve MADM problem under IVPFHSS

The TOPSIS methodology, frequently employed in MADM, presents several advantages for determining and tackling exacerbated decision concerns. TOPSIS methods offer several significant benefits, such as The TOPSIS approach is simple manipulation to pick up and execute. It provides decision-makers with a simple way to assess and evaluate alternatives according to multiple factors. This method enables the concurrent assessment of several criteria or features. It permits decision-makers to analyze alternatives using different parameters. TOPSIS conducts an extensive evaluation by considering both the positive ideal solution (PIS) and the negative ideal solution (NIS). This technique assists in identifying alternates that are almost identical to the positive ideal and least similar to the negative ideal, which leads to an improved equitable assessment. TOPSIS is a robust strategy for various data types involving numerical, grouping, and linguistic variables. This versatility helps decision-makers consider an extensive spectrum of information and opinions in their selections. The TOPSIS approach classifies or encourages the alternatives, providing the outcome simple to analyze and explore. Decision-makers are able to rapidly identify the most appropriate solutions and understand the conflicts among different criteria. TOPSIS is extensively studied and approved in a wide range of fields. Several uses in daily life scenarios for decision-making have shown their efficacy and applicability. This method can handle a number of scales for measuring, including interval-valued, fuzzy, and qualitative information. Furthermore, due to its versatility, it can be used for a broad spectrum of decision-making challenges, irrespective of the nature or type of details provided. In the context of MADM problems, the TOPSIS approach proposed by Hwang and Yoon^51^ is widely used to rank alternatives. In the subsequent segment, we encompass the TOPSIS methodology for IVPFHSS based on the CC.

### Proposed MADM approach

In this section, we consider a set of alternatives ${\mathfrak{H}} = \left\{ {{\mathfrak{H}}^{1} ,{ }{\mathfrak{H}}^{2} ,{ }{\mathfrak{H}}^{3},\ldots ,{ }{\mathfrak{H}}^{s} } \right\}$, a group of experts ${\mathcal{H}} = \left\{ {{\mathcal{H}}_{1} ,{ }{\mathcal{H}}_{2} ,{ }{\mathcal{H}}_{3},\ldots ,{ }{\mathcal{H}}_{n} } \right\}$, and a collection of considered attributes $\varsigma = \left\{ {\varsigma_{1} ,{ }\varsigma_{2} ,{ }\varsigma_{3} ,{ } \ldots ,{ }\varsigma_{m} } \right\}$ with multi-sub-attributes $\varsigma^{\prime} = \left\{ {\left( {\varsigma_{1\rho },\varsigma_{2\rho },\ldots,\varsigma_{m\rho } } \right) \forall \rho \in \left\{ {1, 2, 3, \ldots ,t} \right\}} \right\}$. The weights of professionals and the sub-attributes are given by $\Omega$ = $\left( {\Omega _{1} ,{ }\Omega _{1},\ldots ,{ }\Omega _{n} } \right)^{T}$ and $\gamma = \left( {\gamma_{1} ,{ }\gamma_{2} ,{ }\gamma_{3},\ldots ,{ }\gamma_{\rho } } \right)^{T}$, respectively, where $\Omega _{i} > 0$, $\mathop \sum \limits_{i = 1}^{n}\Omega _{i} = 1$, and $\gamma_{\rho } > 0$, $\mathop \sum \limits_{\rho = 1}^{t} \gamma_{\rho } = 1$. The assortment of multi-sub-attributes can be indicated as $\varsigma^{\prime} = { }\left\{ {\widehat{{\varsigma_{\rho } }}:\rho \in \left\{ {1, 2, \ldots ,t} \right\}} \right\}$. The group of professionals $\left\{ {{\mathcal{H}}_{i} :{ }i{ } = 1,{ }2, \ldots ,{ }n} \right\}$ assess the substitutes $\left\{ {{\mathfrak{H}}^{\left( z \right)} :{ }z{ } = { }1,{ }2,{ } \ldots ,{ }s} \right\}$ because of the particular sub-attributes $\varsigma^{\prime} = { }\left\{ {\widehat{{\varsigma_{\rho } }}:\rho \in \left\{ {1, 2, \ldots ,t} \right\}} \right\}$ in IVPFHSNs form, such as $\left( {{\mathfrak{H}}^{\left( z \right)} ,{ }\varsigma^{\prime}} \right)_{n \times \rho } = \left( {\left[ {{\mathcal{T}}_{{\widehat{{\varsigma_{ij} }}}}^{\ell } ,{ }{\mathcal{T}}_{{\widehat{{\varsigma_{ij} }}}}^{\upsilon } } \right], \left[ {{\mathcal{J}}_{{\widehat{{\varsigma_{ij} }}}}^{\ell } ,{ }{\mathcal{J}}_{{\widehat{{\varsigma_{ij} }}}}^{\upsilon } } \right]} \right)_{n \times \rho }$. The expert's judgment for each substitute in the form of IVPFHSNs can be epitomized as $\Delta_{{\widehat{{\varsigma_{ij} }}}}^{\left( z \right)} = \left( {\left[ {{\mathcal{T}}_{{\widehat{{\varsigma_{ij} }}}}^{\ell } ,{ }{\mathcal{T}}_{{\widehat{{\varsigma_{ij} }}}}^{\upsilon } } \right], \left[ {{\mathcal{J}}_{{\widehat{{\varsigma_{ij} }}}}^{\ell } ,{ }{\mathcal{J}}_{{\widehat{{\varsigma_{ij} }}}}^{\upsilon } } \right]} \right)_{n \times \rho }$, where $0 \le {\mathcal{T}}_{{\widehat{{\varsigma_{ij} }}}}^{\ell } ,{ }{\mathcal{T}}_{{\widehat{{\varsigma_{ij} }}}}^{\upsilon },{\mathcal{J}}_{{\widehat{{\varsigma_{ij} }}}}^{\ell } ,{ }{\mathcal{J}}_{{\widehat{{\varsigma_{ij} }}}}^{\upsilon } \le 1$ and $\left( {{\mathcal{T}}_{{\widehat{{\varsigma_{ij} }}}}^{\upsilon } } \right)^{2} + \left( {{\mathcal{J}}_{{\widehat{{\varsigma_{ij} }}}}^{\upsilon } } \right)^{2} \le 1$, $\forall i, j$.

The proposed TOPSIS technique algorithm is as follows:

**Step 1**. The experts evaluate the preferences of each alternative in the form of IVPFHSNs, such as:

11 $${\left({\mathfrak{H}}^{\left(z\right)}, {{{\varsigma}}}{\prime}\right)}_{n\times \rho }=\begin{array}{c}{\mathcal{H}}_{1}\\ {\mathcal{H}}_{2}\\ \vdots \\ {\mathcal{H}}_{n}\end{array}\left(\begin{array}{cccc}\left(\left[{\mathcal{T}}_{\widehat{{ \varsigma }_{11}}}^{\ell}, {\mathcal{T}}_{\widehat{{ \varsigma }_{11}}}^{\upsilon}\right], \left[{\mathcal{J}}_{\widehat{{ \varsigma }_{11}}}^{\ell}, {\mathcal{J}}_{\widehat{{ \varsigma }_{11}}}^{\upsilon}\right]\right)& \left(\left[{\mathcal{T}}_{\widehat{{ \varsigma }_{12}}}^{\ell}, {\mathcal{T}}_{\widehat{{ \varsigma }_{12}}}^{\upsilon}\right], \left[{\mathcal{J}}_{\widehat{{ \varsigma }_{12}}}^{\ell}, {\mathcal{J}}_{\widehat{{ \varsigma }_{12}}}^{\upsilon}\right]\right)& \cdots & \left(\left[{\mathcal{T}}_{\widehat{{ \varsigma }_{1\rho }}}^{\ell}, {\mathcal{T}}_{\widehat{{ \varsigma }_{1\rho }}}^{\upsilon}\right], \left[{\mathcal{J}}_{\widehat{{ \varsigma }_{1\rho }}}^{\ell}, {\mathcal{J}}_{\widehat{{ \varsigma }_{1\rho }}}^{\upsilon}\right]\right)\\ \left(\left[{\mathcal{T}}_{\widehat{{ \varsigma }_{21}}}^{\ell}, {\mathcal{T}}_{\widehat{{ \varsigma }_{21}}}^{\upsilon}\right], \left[{\mathcal{J}}_{\widehat{{ \varsigma }_{21}}}^{\ell}, {\mathcal{J}}_{\widehat{{ \varsigma }_{21}}}^{\upsilon}\right]\right)& \left(\left[{\mathcal{T}}_{\widehat{{ \varsigma }_{22}}}^{\ell}, {\mathcal{T}}_{\widehat{{ \varsigma }_{22}}}^{\upsilon}\right], \left[{\mathcal{J}}_{\widehat{{ \varsigma }_{22}}}^{\ell}, {\mathcal{J}}_{\widehat{{ \varsigma }_{22}}}^{\upsilon}\right]\right)& \cdots & \left(\left[{\mathcal{T}}_{\widehat{{ \varsigma }_{2\rho }}}^{\ell}, {\mathcal{T}}_{\widehat{{ \varsigma }_{2\rho }}}^{\upsilon}\right], \left[{\mathcal{J}}_{\widehat{{ \varsigma }_{2\rho }}}^{\ell}, {\mathcal{J}}_{\widehat{{ \varsigma }_{2\rho }}}^{\upsilon}\right]\right)\\ \vdots & \vdots & \vdots & \vdots \\ \left(\left[{\mathcal{T}}_{\widehat{{ \varsigma }_{n1}}}^{\ell}, {\mathcal{T}}_{\widehat{{ \varsigma }_{n1}}}^{\upsilon}\right], \left[{\mathcal{J}}_{\widehat{{ \varsigma }_{n1}}}^{\ell}, {\mathcal{J}}_{\widehat{{ \varsigma }_{n1}}}^{\upsilon}\right]\right)& \left(\left[{\mathcal{T}}_{\widehat{{ \varsigma }_{n2}}}^{\ell}, {\mathcal{T}}_{\widehat{{ \varsigma }_{n2}}}^{\upsilon}\right], \left[{\mathcal{J}}_{\widehat{{ \varsigma }_{n2}}}^{\ell}, {\mathcal{J}}_{\widehat{{ \varsigma }_{n2}}}^{\upsilon}\right]\right)& \cdots & \left(\left[{\mathcal{T}}_{\widehat{{ \varsigma }_{n\rho }}}^{\ell}, {\mathcal{T}}_{\widehat{{ \varsigma }_{n\rho }}}^{\upsilon}\right], \left[{\mathcal{J}}_{\widehat{{ \varsigma }_{n\rho }}}^{\ell}, {\mathcal{J}}_{\widehat{{ \varsigma }_{n\rho }}}^{\upsilon}\right]\right)\end{array}\right)$$

**Step 2.** Normalize the decision matrices using the normalization rule and reduce the sub-parameters in the same type.

12 $${{\mathfrak{H}}^{\left(z\right)}}_{\widehat{{ \varsigma }_{ij}}}= \left\{\begin{array}{c}{\left({{\mathfrak{H}}^{\left(z\right)}}_{\widehat{{ \varsigma }_{ij}}}\right)}^{c}={\left(\left[{\mathcal{J}}_{\widehat{{ \varsigma }_{ij}}}^{\ell}, {\mathcal{J}}_{\widehat{{ \varsigma }_{ij}}}^{\upsilon}\right], \left[{\mathcal{T}}_{\widehat{{ \varsigma }_{ij}}}^{\ell}, {\mathcal{T}}_{\widehat{{ \varsigma }_{ij}}}^{\upsilon}\right]\right)}_{n\times \rho } cost\,\, type \,\, sub-parameters\\ {{\mathfrak{H}}^{\left(z\right)}}_{\widehat{{ \varsigma }_{ij}}}= {\left(\left[{\mathcal{T}}_{\widehat{{ \varsigma }_{ij}}}^{\ell}, {\mathcal{T}}_{\widehat{{ \varsigma }_{ij}}}^{\upsilon}\right], \left[{\mathcal{J}}_{\widehat{{ \varsigma }_{ij}}}^{\ell}, {\mathcal{J}}_{\widehat{{ \varsigma }_{ij}}}^{\upsilon}\right]\right)}_{n\times \rho } benefit \,\,type \,\,sub-parameters\end{array}\right.$$

**Step 3.** Evaluate the weighted decision matrices for each alternative ${\overline{\mathfrak{H}}}^{\left( z \right)} = \left( {\Delta_{{\widehat{{\varsigma_{ij} }}}}^{\left( z \right)} } \right)_{n \times \rho }$, where

13 $${\overline{\mathfrak{H}}}^{\left( z \right)} = \gamma_{j}\Omega _{i} \Delta_{{\widehat{{\varsigma_{ij} }}}}^{\left( z \right)} = \left( {\sqrt {1 - \left( {\left( {1 - \left[ {{\mathcal{T}}_{{\Lambda_{{\widehat{{\varsigma_{ij} }}}} }}^{\ell } \left( {{\mathfrak{u}}_{i} } \right),{ }{\mathcal{T}}_{{\Lambda_{{\widehat{{\varsigma_{ij} }}}} }}^{\upsilon } \left( {{\mathfrak{u}}_{i} } \right)} \right]^{2} } \right)^{{\Omega _{i} }} } \right)^{{{\upgamma }_{j} }} } ,\left( {\left( {\left[ {{\mathcal{J}}_{{\Lambda_{{\widehat{{\varsigma_{ij} }}}} }}^{\ell } \left( {{\mathfrak{u}}_{i} } \right),{ }{\mathcal{J}}_{{\Lambda_{{\widehat{{\varsigma_{ij} }}}} }}^{\upsilon } \left( {{\mathfrak{u}}_{i} } \right)} \right]} \right)^{{\Omega _{i} }} } \right)^{{{\upgamma }_{j} }} } \right)$$

14 $${\overline{\mathfrak{H}}}^{\left( z \right)} = \gamma_{j}\Omega _{i} \Delta_{{\widehat{{\varsigma_{ij} }}}}^{\left( z \right)} = \left( {\left( {\left( {\left[ {{\mathcal{T}}_{{\Lambda_{{\widehat{{\varsigma_{ij} }}}} }}^{\ell } \left( {{\mathfrak{u}}_{i} } \right),{ }{\mathcal{T}}_{{\Lambda_{{\widehat{{\varsigma_{ij} }}}} }}^{\upsilon } \left( {{\mathfrak{u}}_{i} } \right)} \right]} \right)^{{\Omega _{i} }} } \right)^{{{\upgamma }_{j} }} ,\sqrt {1 - \left( {\left( {1 - \left[ {{\mathcal{J}}_{{\Lambda_{{\widehat{{\varsigma_{ij} }}}} }}^{\ell } \left( {{\mathfrak{u}}_{i} } \right),{ }{\mathcal{J}}_{{\Lambda_{{\widehat{{\varsigma_{ij} }}}} }}^{\upsilon } \left( {{\mathfrak{u}}_{i} } \right)} \right]^{2} } \right)^{{\Omega _{i} }} } \right)^{{{\upgamma }_{j} }} } } \right)$$

where $\Omega$ = $\left( {\Omega _{1} ,{ }\Omega _{1},\ldots ,{ }\Omega _{n} } \right)^{T}$ and $\gamma = \left( {\gamma_{1} ,{ }\gamma_{2} ,{ }\gamma_{3},\ldots ,{ }\gamma_{\rho } } \right)^{T}$, be the weights of experts and sub-parameters, respectively, such as: $\Omega _{i} > 0$, $\mathop \sum \limits_{i = 1}^{n}\Omega _{i} = 1$, and $\gamma_{\rho } > 0$, $\mathop \sum \limits_{\rho = 1}^{t} \gamma_{\rho } = 1$.

**Step 4.** Determine the PIA and NIA evaluate the indices $\hbar_{{\widehat{{\varsigma_{ij} }}}} = {\text{arg }}max_{z} \left\{ {\theta_{{\widehat{{\varsigma_{ij} }}}}^{\left( z \right)} } \right\}$ and ${\mathfrak{g}}_{{\widehat{{\varsigma_{ij} }}}} = {\text{arg }}min_{z} \left\{ {\theta_{{\widehat{{\varsigma_{ij} }}}}^{\left( z \right)} } \right\}$ such as:

15 $$\Delta _{{\widehat{{\varsigma _{{ij}} }}}}^{{\left( z \right) + }} = \left( {{\mathcal{T}}_{{\widehat{{\varsigma _{{ij}} }}}}^{ + } ,{\text{~}}{\mathcal{J}}_{{\widehat{{\varsigma _{{ij}} }}}}^{ + } } \right)_{{n \times m}} = \left( {{\bar{\mathcal{T}}}_{{\widehat{{\varsigma _{{ij}} }}}}^{{\left( {\hbar _{{\widehat{{\varsigma _{{ij}} }}}} } \right)}} ,{\text{~}}{\bar{\mathcal{J}}}_{{\widehat{{\varsigma _{{ij}} }}}}^{{\left( {\hbar _{{\widehat{{\varsigma _{{ij}} }}}} } \right)}} } \right)$$

and

16 $$\Delta _{{\widehat{{\varsigma _{{ij}} }}}}^{{\left( z \right) - }} = \left( {{\mathcal{T}}_{{\widehat{{\varsigma _{{ij}} }}}}^{ - } ,{\text{~}}{\mathcal{J}}_{{\widehat{{\varsigma _{{ij}} }}}}^{ - } } \right)_{{n \times m}} = \left( {{\bar{\mathcal{T}}}_{{\widehat{{\varsigma _{{ij}} }}}}^{{\left( {{\mathfrak{g}}_{{\widehat{{\varsigma _{{ij}} }}}} } \right)}} ,{\text{~}}{\bar{\mathcal{J}}}_{{\widehat{{\varsigma _{{ij}} }}}}^{{\left( {{\mathfrak{g}}_{{\widehat{{\varsigma _{{ij}} }}}} } \right)}} } \right)$$

**Step 5.** By using the weighted decision matrices ${\overline{\mathfrak{H}}}^{\left( z \right)}$ and PIA $\Delta_{{\widehat{{\varsigma_{ij} }}}}^{{\left( z \right)+ }}$, find the CC such as:

17 $$\begin{gathered} \kappa ^{{\left( z \right)}} = {\mathbb{C}}_{{IVPFHSS}} \left( {{\bar{\mathfrak{H}}}^{{\left( z \right)}} ,{\text{~}}\Delta _{{\widehat{{\varsigma _{{ij}} }}}}^{{\left( z \right) + }} } \right) = \frac{{{\mathcal{C}}_{{IVPFHSS}} \left( {{\bar{\mathfrak{H}}}^{{\left( z \right)}} ,\Delta _{{\widehat{{\varsigma _{{ij}} }}}}^{{\left( z \right) + }} } \right)}}{{\sqrt {{\mathcal{E}}_{{IVPFHSS}} {\bar{\mathfrak{H}}}^{{\left( z \right)}} } \sqrt {{\mathcal{E}}_{{IVPFHSS}} \Delta _{{\widehat{{\varsigma _{{ij}} }}}}^{{\left( z \right) + }} } }} = \hfill \\ \frac{{\mathop \sum \nolimits_{{j = 1}}^{m} \mathop \sum \nolimits_{{i = 1}}^{n} \left( {{\bar{\mathcal{T}}}_{{\widehat{{\varsigma _{{ij}} }}}}^{{\left( z \right)}} {\text{*}}{\mathcal{T}}_{{\widehat{{\varsigma _{{ij}} }}}}^{ + } + {\text{~}}{\bar{\mathcal{J}}}_{{\widehat{{\varsigma _{{ij}} }}}}^{{\left( z \right)}} {\text{*}}{\mathcal{J}}_{{\widehat{{\varsigma _{{ij}} }}}}^{ + } } \right)}}{{\sqrt {\mathop \sum \nolimits_{{j = 1}}^{m} \mathop \sum \nolimits_{{i = 1}}^{n} \left( {\left( {{\bar{\mathcal{T}}}_{{\widehat{{\varsigma _{{ij}} }}}}^{{\left( z \right)}} } \right)^{2} + {\text{~}}\left( {{\bar{\mathcal{J}}}_{{\widehat{{\varsigma _{{ij}} }}}}^{{\left( z \right)}} } \right)^{2} } \right)} \sqrt {\mathop \sum \nolimits_{{j = 1}}^{m} \mathop \sum \nolimits_{{i = 1}}^{n} \left( {\left( {{\mathcal{T}}_{{\widehat{{\varsigma _{{ij}} }}}}^{ + } } \right)^{2} + {\text{~}}\left( {{\mathcal{J}}_{{\widehat{{\varsigma _{{ij}} }}}}^{ + } } \right)^{2} } \right)} }} \hfill \\ \end{gathered}$$

**Step 6.** By using the weighted decision matrices ${\overline{\mathfrak{H}}}^{\left( z \right)}$ and NIA $\Delta_{{\widehat{{\varsigma_{ij} }}}}^{{\left( z \right) - }}$ find the CC:

18 $$\begin{gathered} \tau ^{{\left( z \right)}} = {\mathbb{C}}_{{IVPFHSS}} \left( {{\bar{\mathfrak{H}}}^{{\left( z \right)}} ,{\text{~}}\Delta _{{\widehat{{\varsigma _{{ij}} }}}}^{{\left( z \right) - }} } \right) = \frac{{{\mathcal{C}}_{{IVPFHSS}} \left( {{\bar{\mathfrak{H}}}^{{\left( z \right)}} ,{\text{~~}}\Delta _{{\widehat{{\varsigma _{{ij}} }}}}^{{\left( z \right) - }} } \right)}}{{\sqrt {{\mathcal{E}}_{{IVPFHSS}} {\bar{\mathfrak{H}}}^{{\left( z \right)}} } \sqrt {{\mathcal{E}}_{{IVPFHSS}} {\text{~~}}\Delta _{{\widehat{{\varsigma _{{ij}} }}}}^{{\left( z \right) - }} } }} = \hfill \\ \frac{{\mathop \sum \nolimits_{{j = 1}}^{m} \mathop \sum \nolimits_{{i = 1}}^{n} \left( {{\bar{\mathcal{T}}}_{{\widehat{{\varsigma _{{ij}} }}}}^{{\left( z \right)}} {\text{*}}{\mathcal{T}}_{{\widehat{{\varsigma _{{ij}} }}}}^{ - } + {\text{~}}{\bar{\mathcal{J}}}_{{\widehat{{\varsigma _{{ij}} }}}}^{{\left( z \right)}} {\text{*}}{\mathcal{J}}_{{\widehat{{\varsigma _{{ij}} }}}}^{ - } } \right)}}{{\sqrt {\mathop \sum \nolimits_{{j = 1}}^{m} \mathop \sum \nolimits_{{i = 1}}^{n} \left( {\left( {{\bar{\mathcal{T}}}_{{\widehat{{\varsigma _{{ij}} }}}}^{{\left( z \right)}} } \right)^{2} + {\text{~}}\left( {{\bar{\mathcal{J}}}_{{\widehat{{\varsigma _{{ij}} }}}}^{{\left( z \right)}} } \right)^{2} } \right)} \sqrt {\mathop \sum \nolimits_{{j = 1}}^{m} \mathop \sum \nolimits_{{i = 1}}^{n} \left( {\left( {{\mathcal{T}}_{{\widehat{{\varsigma _{{ij}} }}}}^{ - } } \right)^{2} + {\text{~}}\left( {{\mathcal{J}}_{{\widehat{{\varsigma _{{ij}} }}}}^{ - } } \right)^{2} } \right)} }} \hfill \\ \end{gathered}$$

**Step 7.** Compute the relative closeness to the ideal solution for each alternative.

19 $${\beth }^{{\left( z \right)}} = \frac{{{\daleth }\left( {{\bar{\mathfrak{H}}}^{{\left( z \right)}} ,{\text{~}}\Delta _{{\widehat{{\varsigma _{{ij}} }}}}^{{\left( z \right) - }} } \right)}}{{{\daleth }\left( {{\bar{\mathfrak{H}}}^{{\left( z \right)}} ,{\text{~}}\Delta _{{\widehat{{\varsigma _{{ij}} }}}}^{{\left( z \right) + }} } \right) + {\daleth }\left( {{\bar{\mathfrak{H}}}^{{\left( z \right)}} ,{\text{~}}\Delta _{{\widehat{{\varsigma _{{ij}} }}}}^{{\left( z \right) - }} } \right)}}$$

where $${\daleth }\left( {{\overline{\mathfrak{H}}}^{\left( z \right)} ,{ }\Delta_{{\widehat{{\varsigma_{ij} }}}}^{{\left( z \right)+ }} } \right) = 1 - \kappa^{\left( z \right)}$$ and $${\daleth }\left( {{\overline{\mathfrak{H}}}^{\left( z \right)} ,{ }\Delta_{{\widehat{{\varsigma_{ij} }}}}^{{\left( z \right) - }} } \right) = 1{ } - { }\tau^{\left( z \right)}$$.

**Step 8.** The suitability of a substitute is determined by the closeness coefficient, where a higher value of the closeness coefficient indicates that the alternative is more appropriate.

**Step 9.** Examine the classification.

**Figure 1:**
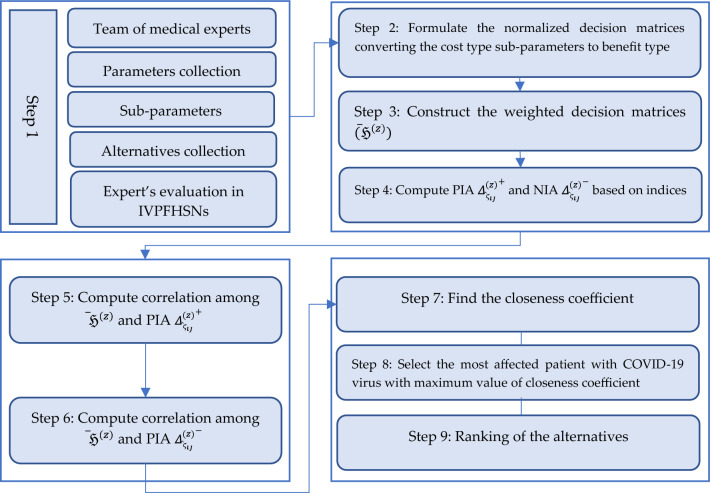
Flow chart of the proposed correlation-based TOPSIS method.

The flow chart of the introduced correlation-based TOPSIS model is presented in the following Fig. 1.

## Challenges in implementing fair bed allocation policies during the COVID-19 pandemic

The COVID-19 pandemic has caused a tremendous burden on healthcare systems worldwide, with an overwhelming number of patients requiring hospitalization and medical care. This has led to the implementation of fair bed allocation policies to ensure patients receive the care they need based on their medical conditions and severity. However, implementing appropriate bed allocation policies has not been without its challenges. One of the primary challenges is the shortage of hospital beds and medical resources. Hospitals have been struggling to meet the demands of COVID-19 patients, leading to the rationing of medical resources, such as ventilators and personal protective equipment. This shortage has made it difficult to allocate beds fairly, as insufficient resources exist. Another challenge is the lack of standardization in bed allocation policies across different healthcare systems. The criteria for bed allocation can vary between hospitals and even between countries. This lack of standardization can lead to disparities in patient care and outcomes, as some patients may have better access to resources than others. Additionally, there may be challenges in determining the severity of a patient’s condition and medical needs. This can be incredibly challenging for patients with underlying health conditions or atypical symptoms. In these cases, it may be challenging to determine which patients require hospitalization and which patients can be safely treated at home. Furthermore, there may be ethical dilemmas when implementing fair bed allocation policies. For example, deciding who should receive medical resources when there is a shortage can be challenging. Healthcare providers may face the difficult decision of choosing between patients, which can be emotionally taxing and have an enduring influence on the patient and their loved ones. Implementing fair bed allocation policies during the COVID-19 pandemic has been challenging due to the shortage of medical resources, lack of standardization in policies, difficulties in determining the severity of patients' conditions, and ethical dilemmas. Addressing these challenges will require collaboration between healthcare systems, policymakers, and healthcare providers to ensure that patients receive the care they need pretty and equitably.

Data-driven decision-making (D3U) has become essential in predicting the spread and stages of the pandemic and in predictive bed allocation. D3U has become integral to our daily lives because it analyzes large amounts of data and provides accurate predictions. It has helped healthcare providers to make informed decisions and allocate resources effectively. Predictive bed allocation has become a crucial aspect of healthcare management, especially during the pandemic, when hospitals have been overwhelmed by the number of patients seeking treatment. The ability to predict the spread and stages of the pandemic has helped policymakers make informed decisions regarding implementing public health measures, such as social distancing and lockdowns. In a D3U (Dedicated COVID-19 Unit), where patients seek treatment for COVID-19, there are several fundamental factors to consider for bed allocation. Firstly, the severity of the patient's symptoms and the level of medical intervention required is critical. Patients with severe symptoms such as respiratory distress, low oxygen saturation levels, or requiring ventilation support may need to be allocated to high-dependency units or ICU beds. Secondly, the patient's age and underlying medical conditions must be considered. Older patients or those with underlying health conditions, such as heart or lung disease, may require specialized care or monitoring. Patients undergoing surgical procedures or weakened immune systems may also require isolation to prevent infections. Thirdly, the availability of medical equipment and resources is essential in bed allocation. Hospitals must have the necessary equipment, such as ventilators, monitors, and oxygen supplies, to ensure proper care and treatment of patients. Lastly, the availability of medical staff and their expertise in treating COVID-19 patients is also a significant factor in bed allocation. Hospitals must have sufficient medical personnel trained in COVID-19 care and management to ensure patients receive the best treatment.

### The severity of the patient’s symptoms and the level of medical intervention $\left( {\varsigma_{1} } \right)$

The severity of a patient's symptoms and the level of medical intervention required are two fundamental factors that must be considered for bed allocation in D3U. The severity of a patient's symptoms is an essential indicator of their condition and the level of medical intervention needed to manage their illness. For instance, a patient with severe symptoms may require a higher medical intervention than a mild one. This could include interventions such as oxygen therapy, IV fluids, or mechanical ventilation. The level of medical intervention required also plays a critical role in determining the appropriate bed allocation for patients.

- Patients who require a high level of medical intervention, such as those needing critical care or intensive care, will require specialized beds equipped with the necessary medical equipment and facilities.
- Patients who require less medical intervention may be allocated to less specialized beds. The severity of a patient's symptoms and the level of medical intervention required are essential factors to consider in bed allocation in D3U. These factors can help to ensure that patients receive the appropriate level of care and are allocated to the most suitable beds based on their medical needs.

### Patient's age and underlying medical conditions $\left( {\varsigma_{2} } \right)$

When allocating beds in a D3U, it is important to consider the patient’s age and underlying medical conditions. Age and medical conditions are significant factors that can affect a patient’s recovery and the level of medical intervention they require.

- Older patients and those with underlying medical conditions such as diabetes, heart disease, or respiratory problems may require more medical intervention and monitoring. They may also take longer to recover, and their conditions deteriorate more rapidly. Therefore, it is essential to allocate beds to ensure patients receive the appropriate care and support.
- Younger patients or those with no underlying medical conditions may require less medical intervention and may recover more quickly. Allocating beds to these patients may help free up resources for those requiring more critical care.

It is also important to note that some medical conditions may increase the risk of complications, such as respiratory failure, which requires immediate and intensive care. In such cases, allocating beds to patients with these conditions must be prioritized to ensure timely and effective treatment. Patient age and underlying medical conditions are critical factors that must be considered when allocating beds in a D3U. Careful consideration of these factors can help to ensure that patients receive the appropriate level of care and that resources are allocated efficiently.

### Medical equipment and resources an essential factors in bed allocation $\left( {\varsigma_{3} } \right)$

When allocating beds in a D3U, it is essential to consider the availability of medical equipment and resources. The type of medical equipment and resources needed for each patient can vary significantly depending on their medical condition and the severity of their symptoms. For example, patients with severe respiratory distress may require access to ventilators, while patients with cardiac issues may need access to specialized monitoring equipment. Additionally, patients with infectious diseases may require isolation rooms and specialized personal protective equipment to prevent the spread of the disease. The availability of medical equipment and resources can vary depending on the location and size of the D3U. Sometimes, larger hospitals may have more extensive resources and equipment than smaller clinics or medical centers. Therefore, it is crucial to consider the medical facility’s capabilities and the resources available when allocating beds to patients. Moreover, it is also essential to ensure that the medical equipment and resources are adequately maintained and serviced to ensure proper functioning. Regular maintenance and service of medical equipment can help prevent equipment failure and ensure patients receive the necessary medical care promptly. Medical equipment and resources are critical factors that must be considered when allocating beds in a D3U. The availability and proper functioning of medical equipment can significantly impact patient outcomes and can be the difference between life and death in some cases. Therefore, it is crucial to ensure that medical facilities have access to the necessary equipment and resources and that they are adequately maintained to ensure optimal patient care.

### Availability of medical staff and their expertise in treating COVID-19 patients $\left( {\varsigma_{4} } \right)$

The availability of medical staff and their expertise in treating COVID-19 patients is crucial in bed allocation for D3U. The demand for healthcare workers has increased significantly due to the pandemic, and hospitals must ensure they have enough staff to handle the patient load. Not only is the number of available medical staff important, but also their level of expertise in treating COVID-19 patients. COVID-19 is a new and complex disease, and treating it requires specialized knowledge and skills. Hospitals must ensure that their staff is properly trained in the latest treatment protocols and have the necessary equipment to provide high-quality care to patients. The shortage of medical staff and the burden on those working in the healthcare industry have been major challenges during the COVID-19 pandemic. Staffing shortages can lead to increased workloads and burnout, compromising patient care quality. Hospitals must prioritize the safety and well-being of their staff and provide them with the necessary resources to do their job effectively. In addition to ensuring adequate staffing levels and expertise, hospitals must also consider the potential for staff members to contract the virus themselves. This risk can be mitigated by providing adequate personal protective equipment (PPE) and ensuring proper infection control protocols are in place. The availability of medical staff and their expertise in treating COVID-19 patients is critical in bed allocation for D3U. Hospitals must ensure that they have enough staff with the appropriate training and equipment to provide high-quality care to patients while also prioritizing the safety and well-being of their staff.

### Numerical example

Suppose ${\mathfrak{T}} = \left\{ {{\mathfrak{T}}^{1} ,{ }{\mathfrak{T}}^{2} ,{ }{\mathfrak{T}}^{3} ,{ }{\mathfrak{T}}^{4} } \right\}$ be a collection of different COVID-19 patients (alternatives) and ${\mathcal{H}} = \left\{ {{\mathcal{H}}_{1} ,{ }{\mathcal{H}}_{2} ,{ }{\mathcal{H}}_{3} ,{ }{\mathcal{H}}_{4} } \right\}$ be a team of medical experts with weights $\left( {0.2,{ }0.4,{ }0.3,{ }0.1} \right)^{T}$. The hospital has only one bed available, and the medical experts evaluate which patient is most affected by the COVID-19 virus. First of all, medical experts considered the criteria for bed allocation for COVID-19 patients, such as $\varsigma = \left\{ {\varsigma_{1} ,{ }\varsigma_{2} ,{ }\varsigma_{3} ,{ }\varsigma_{4} } \right\}$, where $\varsigma_{1} :$ the severity of the patient’s symptoms and the level of medical intervention, $\varsigma_{2} :$ patient’s age and underlying medical conditions, $\varsigma_{3} :$ medical equipment and resources is an essential factor in bed allocation, and $\varsigma_{4} :$ availability of medical staff and their expertise in treating COVID-19 patients. The severity of the patient’s symptoms and the level of medical intervention = $\varsigma_{1}$  = $\left\{ {\varsigma_{11} = {\text{Patients }}\;{\text{who}}\;{\text{ require}}\;{\text{ a }}\;{\text{high}}\;{\text{ level }}\;{\text{of }}\;{\text{medical}}\;{\text{ intervention}},{ }\varsigma_{12} = {\text{Patients }}\;{\text{who }}\;{\text{require}}\;{\text{ less}}\;{\text{ medical}}\;{\text{ intervention}}} \right\}$, Patient’s age and underlying medical conditions = $\varsigma_{2}$ = $\left\{ {\varsigma_{21} = {\text{Older patients}},{ }\varsigma_{22} = {\text{Younger patients}}} \right\}$ Medical equipment and resources an essential factors in bed allocation = $\varsigma_{3}$ = $\left\{ {\varsigma_{31} = Medical \;equipment\; and \;resources\; is \;an\; essential \;factor\; in\; bed \;allocation} \right\}$ and Availability of medical staff and their expertise in treating COVID-19 patients = $\varsigma_{4}$  = $\left\{ {\varsigma_{41} = availability \;of \;medical \;staff, \;\varsigma_{42} = availability \;of\; experienced\; medical\; experts} \right\}$. Let $\varsigma^{\prime} = \varsigma_{1} \times \varsigma_{2} \times \varsigma_{3} \times \varsigma_{4}$ represents the set of sub-parameters. 

$$\begin{gathered} \varsigma ^{\prime} = \varsigma _{1} \times \varsigma _{2} \times \varsigma _{3} \times \varsigma _{4} = \left\{ {\varsigma _{{11}} ,~\varsigma _{{12}} } \right\} \times \left\{ {\varsigma _{{21}} ,~\varsigma _{{22}} } \right\} \times \left\{ {\varsigma _{{31}} } \right\} \times \left\{ {\varsigma _{{41}} ,~\varsigma _{{42}} } \right\} = \hfill \\ \left\{ \begin{gathered} \left( {\varsigma _{{11}} ,~\varsigma _{{21}} ,~\varsigma _{{31}} ,~\varsigma _{{41}} } \right),~\left( {\varsigma _{{11}} ,~\varsigma _{{21}} ,~\varsigma _{{31}} ,~\varsigma _{{42}} } \right),~\left( {\varsigma _{{11}} ,~\varsigma _{{22}} ,~\varsigma _{{31}} ,~\varsigma _{{41}} } \right),~\left( {\varsigma _{{11}} ,~\varsigma _{{22}} ,~\varsigma _{{31}} ,~\varsigma _{{42}} } \right), \hfill \\ \left( {\varsigma _{{12}} ,~\varsigma _{{21}} ,~\varsigma _{{31}} ,~\varsigma _{{41}} } \right),~\left( {\varsigma _{{12}} ,~\varsigma _{{21}} ,~\varsigma _{{31}} ,~\varsigma _{{42}} } \right),~\left( {\varsigma _{{12}} ,~\varsigma _{{22}} ,~\varsigma _{{31}} ,~\varsigma _{{41}} } \right),~\left( {\varsigma _{{12}} ,~\varsigma _{{22}} ,~\varsigma _{{31}} ,~\varsigma _{{42}} } \right) \hfill \\ \end{gathered} \right\} \hfill \\ \end{gathered}$$, $\varsigma^{\prime} = \left\{ {\hat{\varsigma }_{1} ,\hat{\varsigma }_{2},\hat{\varsigma }_{3},\hat{\varsigma }_{4},\hat{\varsigma }_{5},\hat{\varsigma }_{6},\hat{\varsigma }_{7},\hat{\varsigma }_{8} } \right\}$ shows the collection of sub-attributes with weights $\gamma_{j} = \left( {.2, .05, .15, .1, .15, .18, .05, .12} \right)^{T}$. In the context of COVID-19 patients, various aspects are considered to evaluate the patient's health status, such as oxygen saturation, respiratory rate, blood pressure, etc. A group of professionals evaluates the patients based on these aspects and provides their preferences for each patient in the IVPFHSN form. The medical experts judge the most affected patient who needs bed on an emergency basis considering the above-stated symptoms under deliberated parameters. Tables 1, 2, 3 and 4 show the evaluation of COVID-19 patients based on various sub-attributes of these aspects. These evaluations are used to determine the most affected COVID-19 patients using the TOPSIS-based MADM problem. The TOPSIS approach evaluates the positive and negative ideal solutions for the DM process. It helps to accumulate the PIS and NIS and prolong the methodology for IVPFHSS built on the CC. The experts’ weights and attributes determine the most affected patient. The structure of the planned TOPSIS method is used to determine each patient's closeness coefficient, and the exaggerated value of the closeness coefficient spectacles the most affected patient and deserves bed on an urgent basis.

**Table 1:** Expert’s estation for ${\mathfrak{T}}^{1}$ in IVPFHSNs.

	$\hat{\varsigma }_{1}$	$\hat{\varsigma }_{2}$	$\hat{\varsigma }_{3}$	$\hat{\varsigma }_{4}$	$\hat{\varsigma }_{5}$	$\hat{\varsigma }_{6}$	$\hat{\varsigma }_{7}$	$\hat{\varsigma }_{8}$
${\mathcal{H}}_{1}$	$\left(\left[0.4, 0.5\right],\left[0.2, 0.6\right]\right)$	$\left(\left[0.3, 0.6\right],\left[0.1, 0.3\right]\right)$	$\left(\left[0.4, 0.6\right],\left[0.2, 0.4\right]\right)$	$\left(\left[0.2, 0.3\right],\left[0.2, 0.5\right]\right)$	$\left(\left[0.3, 0.6\right],\left[0.1, 0.3\right]\right)$	$\left(\left[0.3, 0.5\right],\left[0.2, 0.5\right]\right)$	$\left(\left[0.4, 0.5\right],\left[0.2, 0.5\right]\right)$	$\left(\left[0.2, 0.4\right],\left[0.2, 0.6\right]\right)$
${\mathcal{H}}_{2}$	$\left(\left[0.2, 0.5\right],\left[0.2, 0.4\right]\right)$	$\left(\left[0.2, 0.4\right],\left[0.4, 0.5\right]\right)$	$\left(\left[0.2, 0.3\right],\left[0.4, 0.6\right]\right)$	$\left(\left[0.2, 0.6\right],\left[0.5, 0.7\right]\right)$	$\left(\left[0.2, 0.5\right],\left[0.2, 0.4\right]\right)$	$\left(\left[0.2, 0.7\right],\left[0.1, 0.4\right]\right)$	$\left(\left[0.2, 0.7\right],\left[0.2, 0.6\right]\right)$	$\left(\left[0.2, 0.5\right],\left[0.4, 0.7\right]\right)$
${\mathcal{H}}_{3}$	$\left(\left[0.3, 0.5\right],\left[0.4, 0.7\right]\right)$	$\left(\left[0.4, 0.6\right],\left[0.2, 0.3\right]\right)$	$\left(\left[0.4, 0.7\right],\left[0.3, 0.3\right]\right)$	$\left(\left[0.3, 0.5\right],\left[0.1, 0.4\right]\right)$	$\left(\left[0.1, 0.5\right],\left[0.1, 0.8\right]\right)$	$\left(\left[0.3, 0.5\right],\left[0.1, 0.4\right]\right)$	$\left(\left[0.3, 0.5\right],\left[0.1, 0.4\right]\right)$	$\left(\left[0.3, 0.6\right],\left[0.2, 0.5\right]\right)$
${\mathcal{H}}_{4}$	$\left(\left[0.2, 0.6\right],\left[0.3, 0.7\right]\right)$	$\left(\left[0.1, 0.5\right],\left[0.3, 0.8\right]\right)$	$\left(\left[0.2, 0.4\right],\left[0.3, 0.5\right]\right)$	$\left(\left[0.1, 0.4\right],\left[0.5, 0.9\right]\right)$	$\left(\left[0.5, 0.6\right],\left[0.3, 0.5\right]\right)$	$\left(\left[0.2, 0.2\right],\left[0.4, 0.5\right]\right)$	$\left(\left[0.1, 0.3\right],\left[0.2, 0.7\right]\right)$	$\left(\left[0.2, 0.3\right],\left[0.4, 0.6\right]\right)$

**Table 2:** Expert’s estimation for ${\mathfrak{T}}^{2}$ in IVPFHSNs.

	$\hat{\varsigma }_{1}$	$\hat{\varsigma }_{2}$	$\hat{\varsigma }_{3}$	$\hat{\varsigma }_{4}$	$\hat{\varsigma }_{5}$	$\hat{\varsigma }_{6}$	$\hat{\varsigma }_{7}$	$\hat{\varsigma }_{8}$
${\mathcal{H}}_{1}$	$\left(\left[0.1, 0.3\right],\left[0.2, 0.5\right]\right)$	$\left(\left[0.2, 0.6\right],\left[0.1, 0.7\right]\right)$	$\left(\left[0.2, 0.4\right],\left[0.2, 0.5\right]\right)$	$\left(\left[0.1, 0.3\right],\left[0.3, 0.5\right]\right)$	$\left(\left[0.3, 0.5\right],\left[0.2, 0.3\right]\right)$	$\left(\left[0.1, 0.7\right],\left[0.2, 0.5\right]\right)$	$\left(\left[0.1, 0.5\right],\left[0.2, 0.4\right]\right)$	$\left(\left[0.1, 0.6\right],\left[0.2, 0.6\right]\right)$
${\mathcal{H}}_{2}$	$\left(\left[0.2, 0.7\right],\left[0.2, 0.4\right]\right)$	$\left(\left[0.2, 0.6\right],\left[0.1, 0.5\right]\right)$	$\left(\left[0.2, 0.6\right],\left[0.4, 0.5\right]\right)$	$\left(\left[0.2, 0.5\right],\left[0.2, 0.4\right]\right)$	$\left(\left[0.2, 0.4\right],\left[0.3, 0.5\right]\right)$	$\left(\left[0.2, 0.3\right],\left[0.1, 0.6\right]\right)$	$\left(\left[0.2, 0.5\right],\left[0.2, 0.6\right]\right)$	$\left(\left[0.2, 0.4\right],\left[0.4, 0.5\right]\right)$
${\mathcal{H}}_{3}$	$\left(\left[0.3, 0.5\right],\left[0.1, 0.6\right]\right)$	$\left(\left[0.4, 0.5\right],\left[0.2, 0.4\right]\right)$	$\left(\left[0.1, 0.5\right],\left[0.3, 0.6\right]\right)$	$\left(\left[0.3, 0.4\right],\left[0.1, 0.5\right]\right)$	$\left(\left[0.1, 0.7\right],\left[0.1, 0.4\right]\right)$	$\left(\left[0.3, 0.5\right],\left[0.1, 0.3\right]\right)$	$\left(\left[0.3, 0.5\right],\left[0.1, 0.4\right]\right)$	$\left(\left[0.3, 0.5\right],\left[0.2, 0.4\right]\right)$
${\mathcal{H}}_{4}$	$\left(\left[0.1, 0.4\right],\left[0.2, 0.7\right]\right)$	$\left(\left[0.3, 0.6\right],\left[0.4, 0.7\right]\right)$	$\left(\left[0.2, 0.4\right],\left[0.3, 0.8\right]\right)$	$\left(\left[0.1, 0.3\right],\left[0.2, 0.6\right]\right)$	$\left(\left[0.3, 0.5\right],\left[0.3, 0.6\right]\right)$	$\left(\left[0.2, 0.8\right],\left[0.1, 0.4\right]\right)$	$\left(\left[0.3, 0.4\right],\left[0.2, 0.7\right]\right)$	$\left(\left[0.3, 0.5\right],\left[0.1, 0.6\right]\right)$

**Table 3:** Expert’s estimation for ${\mathfrak{T}}^{3}$ in IVPFHSNs.

	$\hat{\varsigma }_{1}$	$\hat{\varsigma }_{2}$	$\hat{\varsigma }_{3}$	$\hat{\varsigma }_{4}$	$\hat{\varsigma }_{5}$	$\hat{\varsigma }_{6}$	$\hat{\varsigma }_{7}$	$\hat{\varsigma }_{8}$
${\mathcal{H}}_{1}$	$\left(\left[0.2, 0.7\right],\left[0.2, 0.4\right]\right)$	$\left(\left[0.2, 0.6\right],\left[0.1, 0.3\right]\right)$	$\left(\left[0.2, 0.5\right],\left[0.2, 0.8\right]\right)$	$\left(\left[0.2, 0.6\right],\left[0.2, 0.3\right]\right)$	$\left(\left[0.3, 0.5\right],\left[0.1, 0.3\right]\right)$	$\left(\left[0.1, 0.5\right],\left[0.2, 0.4\right]\right)$	$\left(\left[0.2, 0.4\right],\left[0.1, 0.5\right]\right)$	$\left(\left[0.2, 0.4\right],\left[0.2, 0.5\right]\right)$
${\mathcal{H}}_{2}$	$\left(\left[0.2, 0.5\right],\left[0.2, 0.3\right]\right)$	$\left(\left[0.2, 0.5\right],\left[0.2, 0.4\right]\right)$	$\left(\left[0.2, 0.4\right],\left[0.4, 0.5\right]\right)$	$\left(\left[0.2, 0.5\right],\left[0.2, 0.4\right]\right)$	$\left(\left[0.2, 0.4\right],\left[0.2, 0.5\right]\right)$	$\left(\left[0.2, 0.4\right],\left[0.1, 0.9\right]\right)$	$\left(\left[0.1, 0.3\right],\left[0.2, 0.4\right]\right)$	$\left(\left[0.2, 0.5\right],\left[0.4, 0.6\right]\right)$
${\mathcal{H}}_{3}$	$\left(\left[0.1, 0.4\right],\left[0.2, 0.4\right]\right)$	$\left(\left[0.3, 0.8\right],\left[0.2, 0.3\right]\right)$	$\left(\left[0.4, 0.6\right],\left[0.3, 0.4\right]\right)$	$\left(\left[0.3, 0.4\right],\left[0.1, 0.5\right]\right)$	$\left(\left[0.1, 0.3\right],\left[0.1, 0.6\right]\right)$	$\left(\left[0.3, 0.4\right],\left[0.1, 0.5\right]\right)$	$\left(\left[0.3, 0.5\right],\left[0.3, 0.4\right]\right)$	$\left(\left[0.3, 0.5\right],\left[0.2, 0.4\right]\right)$
${\mathcal{H}}_{4}$	$\left(\left[0.3, 0.7\right],\left[0.2, 0.5\right]\right)$	$\left(\left[0.1, 0.4\right],\left[0.3, 0.8\right]\right)$	$\left(\left[0.2, 0.5\right],\left[0.2, 0.4\right]\right)$	$\left(\left[0.4, 0.6\right],\left[0.3, 0.7\right]\right)$	$\left(\left[0.2, 0.4\right],\left[0.5, 0.7\right]\right)$	$\left(\left[0.4, 0.8\right],\left[0.1, 0.3\right]\right)$	$\left(\left[0.2, 0.5\right],\left[0.5, 0.8\right]\right)$	$\left(\left[0.3, 0.7\right],\left[0.2, 0.4\right]\right)$

**Table 4:** Expert's estimation for ${\mathfrak{T}}^{4}$ in IVPFHSNs.

	$\hat{\varsigma }_{1}$	$\hat{\varsigma }_{2}$	$\hat{\varsigma }_{3}$	$\hat{\varsigma }_{4}$	$\hat{\varsigma }_{5}$	$\hat{\varsigma }_{6}$	$\hat{\varsigma }_{7}$	$\hat{\varsigma }_{8}$
${\mathcal{H}}_{1}$	$\left(\left[0.3, 0.6\right],\left[0.1, 0.4\right]\right)$	$\left(\left[0.3, 0.5\right],\left[0.2, 0.7\right]\right)$	$\left(\left[0.2, 0.5\right],\left[0.2, 0.8\right]\right)$	$\left(\left[0.2, 0.9\right],\left[0.3, 0.4\right]\right)$	$\left(\left[0.1, 0.7\right],\left[0.1, 0.4\right]\right)$	$\left(\left[0.2, 0.6\right],\left[0.1, 0.5\right]\right)$	$\left(\left[0.2, 0.4\right],\left[0.3, 0.5\right]\right)$	$\left(\left[0.3, 0.4\right],\left[0.6, 0.7\right]\right)$
${\mathcal{H}}_{2}$	$\left(\left[0.4, 0.7\right],\left[0.3, 0.5\right]\right)$	$\left(\left[0.1, 0.4\right],\left[0.3, 0.6\right]\right)$	$\left(\left[0.3, 0.4\right],\left[0.6, 0.7\right]\right)$	$\left(\left[0.1, 0.5\right],\left[0.2, 0.7\right]\right)$	$\left(\left[0.3, 0.5\right],\left[0.1, 0.4\right]\right)$	$\left(\left[0.1, 0.6\right],\left[0.2, 0.6\right]\right)$	$\left(\left[0.1, 0.3\right],\left[0.2, 0.4\right]\right)$	$\left(\left[0.3, 0.4\right],\left[0.2, 0.7\right]\right)$
${\mathcal{H}}_{3}$	$\left(\left[0.3, 0.6\right],\left[0.4, 0.8\right]\right)$	$\left(\left[0.2, 0.8\right],\left[0.1, 0.3\right]\right)$	$\left(\left[0.1, 0.6\right],\left[0.2, 0.5\right]\right)$	$\left(\left[0.3, 0.4\right],\left[0.1, 0.5\right]\right)$	$\left(\left[0.4, 0.5\right],\left[0.2, 0.4\right]\right)$	$\left(\left[0.3, 0.5\right],\left[0.1, 0.6\right]\right)$	$\left(\left[0.2, 0.4\right],\left[0.1, 0.9\right]\right)$	$\left(\left[0.2, 0.5\right],\left[0.2, 0.4\right]\right)$
${\mathcal{H}}_{4}$	$\left(\left[0.2, 0.6\right],\left[0.3, 0.5\right]\right)$	$\left(\left[0.5, 0.7\right],\left[0.3, 0.5\right]\right)$	$\left(\left[0.4, 0.5\right],\left[0.2, 0.8\right]\right)$	$\left(\left[0.5, 0.6\right],\left[0.4, 0.7\right]\right)$	$\left(\left[0.2, 0.4\right],\left[0.2, 0.5\right]\right)$	$\left(\left[0.2, 0.6\right],\left[0.1, 0.5\right]\right)$	$\left(\left[0.2, 0.5\right],\left[0.2, 0.4\right]\right)$	$\left(\left[0.3, 0.6\right],\left[0.4, 0.7\right]\right)$

Step 1. The experts deliver their preferences for alternatives ${\mathfrak{T}} = \left\{ {{\mathfrak{T}}^{1} ,{ }{\mathfrak{T}}^{2} ,{ }{\mathfrak{T}}^{3} ,{ }{\mathfrak{T}}^{4} } \right\}$ in the form of IVPFHSNs, such as in the following Tables 1, 2, 3 and 4:

Step 2: Indicates that all considered parameters are the same type, so there is no need to normalize them. This means that the IVPFHSN values provided by the expert group for each patient in Tables 1, 2, 3 and 4 can be directly used as the decision matrix for each alternative.

Step 3: Tables 5, 6, 7 and 8 show the weighted decision matrix ${\overline{\mathfrak{T}}}^{\left( z \right)} = \left( {\Delta_{{\widehat{{\varsigma_{ij} }}}}^{\left( z \right)} } \right)_{n \times \rho }$ for each patient, the values in each cell represent the weighted IVPFHSN score for that particular sub-attribute.

**Table 5:** Weighted decision matrix for ${\overline{\mathfrak{T}}}^{\left( 1 \right)}$.

	$\hat{\varsigma }_{1}$	$\hat{\varsigma }_{2}$	$\hat{\varsigma }_{3}$	$\hat{\varsigma }_{4}$	$\hat{\varsigma }_{5}$	$\hat{\varsigma }_{6}$	$\hat{\varsigma }_{7}$	$\hat{\varsigma }_{8}$
${\mathcal{H}}_{1}$	$$\left(\begin{array}{c}\left[0.0456, 0.0697\right], \\ \left[0.4621, 0.5069\right]\end{array}\right)$$	$$\left(\begin{array}{c}\left[0.0271, 0.0353\right], \\ \left[0.5234, 0.5681\right]\end{array}\right)$$	$$\left(\begin{array}{c}\left[0.0361, 0.0452\right], \\ \left[0.4953, 0.5271\right]\end{array}\right)$$	$$\left(\begin{array}{c}\left[0.0241, 0.0371\right], \\ \left[0.5231, 0.5472\right]\end{array}\right)$$	$$\left(\begin{array}{c}\left[0.0271, 0.0343\right], \\ \left[0.5373, 0.5540\right]\end{array}\right)$$	$$\left(\begin{array}{c}\left[0.0246, 0.0414\right], \\ \left[0.4912, 0.5296\right]\end{array}\right)$$	$$\left(\begin{array}{c}\left[0.0224, 0.0376\right], \\ \left[0.5143, 0.5584\right]\end{array}\right)$$	$$\left(\begin{array}{c}\left[0.0265, 0.0354\right], \\ \left[0.4853, 0.5176\right]\end{array}\right)$$
${\mathcal{H}}_{2}$	$$\left(\begin{array}{c}\left[0.0147, 0.0293\right],\\ \left[0.6855, 0.7226\right]\end{array}\right)$$	$$\left(\begin{array}{c}\left[0.0235, 0.0379\right], \\ \left[0.5936, 0.6517\right]\end{array}\right)$$	$$\left(\begin{array}{c}\left[0.0279, 0.0364\right], \\ \left[0.6194, 0.6887\right]\end{array}\right)$$	$$\left(\begin{array}{c}\left[0.0427, 0.0567\right], \\ \left[0.5836, 0.5906\right]\end{array}\right)$$	$$\left(\begin{array}{c}\left[0.0357, 0.0397\right], \\ \left[0.5437, 0.6725\right]\end{array}\right)$$	$$\left(\begin{array}{c}\left[0.0314, 0.0491\right],\\ \left[0.6754, 0.7323\right]\end{array}\right)$$	$$\left(\begin{array}{c}\left[0.0327, 0.0467\right], \\ \left[0.5836, 0.6106\right]\end{array}\right)$$	$$\left(\begin{array}{c}\left[0.0349, 0.0473\right], \\ \left[0.5749, 0.6387\right]\end{array}\right)$$
${\mathcal{H}}_{3}$	$$\left(\begin{array}{c}\left[0.0363, 0.0439\right], \\ \left[0.5814, 0.6258\right]\end{array}\right)$$	$$\left(\begin{array}{c}\left[0.0363, 0.0438\right], \\ \left[0.6365, 0.7053\right]\end{array}\right)$$	$$\left(\begin{array}{c}\left[0.0214, 0.0394\right], \\ \left[0.5876, 0.6291\right]\end{array}\right)$$	$$\left(\begin{array}{c}\left[0.0236, 0.0316\right], \\ \left[0.5961, 0.6423\right]\end{array}\right)$$	$$\left(\begin{array}{c}\left[0.0363, 0.0416\right], \\ \left[0.6465, 0.7053\right]\end{array}\right)$$	$$\left(\begin{array}{c}\left[0.0253, 0.0375\right], \\ \left[0.5816, 0.6458\right]\end{array}\right)$$	$$\left(\begin{array}{c}\left[0.0263, 0.0416\right], \\ \left[0.5924, 0.6123\right]\end{array}\right)$$	$$\left(\begin{array}{c}\left[0.0242, 0.0451\right], \\ \left[0.5867, 0.6327\right]\end{array}\right)$$
${\mathcal{H}}_{4}$	$$\left(\begin{array}{c}\left[0.0427, 0.0567\right], \\ \left[0.5836, 0.5906\right]\end{array}\right)$$	$$\left(\begin{array}{c}\left[0.0253, 0.0375\right], \\ \left[0.5816, 0.6458\right]\end{array}\right)$$	$$\left(\begin{array}{c}\left[0.0242, 0.0451\right], \\ \left[0.5867, 0.6327\right]\end{array}\right)$$	$$\left(\begin{array}{c}\left[0.0246, 0.0414\right], \\ \left[0.4912, 0.5296\right]\end{array}\right)$$	$$\left(\begin{array}{c}\left[0.0271, 0.0353\right], \\ \left[0.5234, 0.5681\right]\end{array}\right)$$	$$\left(\begin{array}{c}\left[0.0432, 0.0477\right], \\ \left[0.5534, 0.5748\right]\end{array}\right)$$	$$\left(\begin{array}{c}\left[0.0174, 0.0539\right],\\ \left[0.6945, 0.7962\right]\end{array}\right)$$	$$\left(\begin{array}{c}\left[0.0327, 0.0443\right], \\ \left[0.5574, 0.5648\right]\end{array}\right)$$

**Table 6:** Weighted decision matrix for ${\overline{\mathfrak{T}}}^{\left( 2 \right)}$.

	$\hat{\varsigma }_{1}$	$\hat{\varsigma }_{2}$	$\hat{\varsigma }_{3}$	$\hat{\varsigma }_{4}$	$\hat{\varsigma }_{5}$	$\hat{\varsigma }_{6}$	$\hat{\varsigma }_{7}$	$\hat{\varsigma }_{8}$
${\mathcal{H}}_{1}$	$$\left(\begin{array}{c}\left[0.0236, 0.0316\right], \\ \left[0.5961, 0.6423\right]\end{array}\right)$$	$$\left(\begin{array}{c}\left[0.0235, 0.0397\right], \\ \left[0.5963, 0.6171\right]\end{array}\right)$$	$$\left(\begin{array}{c}\left[0.0174, 0.0539\right],\\ \left[0.6945, 0.7962\right]\end{array}\right)$$	$$\left(\begin{array}{c}\left[0.0375, 0.0379\right], \\ \left[0.6373, 0.6852\right]\end{array}\right)$$	$$\left(\begin{array}{c}\left[0.0512, 0.0576\right], \\ \left[0.6163, 0.6260\right]\end{array}\right)$$	$$\left(\begin{array}{c}\left[0.0344, 0.0419\right],\\ \left[0.7245, 0.7532\right]\end{array}\right)$$	$$\left(\begin{array}{c}\left[0.0472, 0.0476\right], \\ \left[0.5763, 0.5860\right]\end{array}\right)$$	$$\left(\begin{array}{c}\left[0.0234, 0.0357\right], \\ \left[0.6249, 0.6378\right]\end{array}\right)$$
${\mathcal{H}}_{2}$	$$\left(\begin{array}{c}\left[0.0432, 0.0477\right], \\ \left[0.5534, 0.5748\right]\end{array}\right)$$	$$\left(\begin{array}{c}\left[0.0224, 0.0376\right], \\ \left[0.5143, 0.5584\right]\end{array}\right)$$	$$\left(\begin{array}{c}\left[0.0314, 0.0491\right],\\ \left[0.6754, 0.7323\right]\end{array}\right)$$	$$\left(\begin{array}{c}\left[0.0253, 0.0375\right], \\ \left[0.5816, 0.6458\right]\end{array}\right)$$	$$\left(\begin{array}{c}\left[0.0214, 0.0394\right], \\ \left[0.5876, 0.6291\right]\end{array}\right)$$	$$\left(\begin{array}{c}\left[0.0147, 0.0293\right],\\ \left[0.6855, 0.7226\right]\end{array}\right)$$	$$\left(\begin{array}{c}\left[0.0327, 0.0443\right], \\ \left[0.5574, 0.5648\right]\end{array}\right)$$	$$\left(\begin{array}{c}\left[0.0253, 0.0357\right], \\ \left[0.5809, 0.6085\right]\end{array}\right)$$
${\mathcal{H}}_{3}$	$$\left(\begin{array}{c}\left[0.0253, 0.0375\right], \\ \left[0.5816, 0.6458\right]\end{array}\right)$$	$$\left(\begin{array}{c}\left[0.0464, 0.0541\right], \\ \left[0.4921, 0.5061\right]\end{array}\right)$$	$$\left(\begin{array}{c}\left[0.0327, 0.0443\right], \\ \left[0.5574, 0.5648\right]\end{array}\right)$$	$$\left(\begin{array}{c}\left[0.0265, 0.0345\right], \\ \left[0.4935, 0.5467\right]\end{array}\right)$$	$$\left(\begin{array}{c}\left[0.0564, 0.0741\right], \\ \left[0.9721, 0.9869\right]\end{array}\right)$$	$$\left(\begin{array}{c}\left[0.0242, 0.0377\right], \\ \left[0.5234, 0.5448\right]\end{array}\right)$$	$$\left(\begin{array}{c}\left[0.0127, 0.0253\right], \\ \left[0.5134, 0.5248\right]\end{array}\right)$$	$$\left(\begin{array}{c}\left[0.0432, 0.0477\right], \\ \left[0.5534, 0.5748\right]\end{array}\right)$$
${\mathcal{H}}_{4}$	$$\left(\begin{array}{c}\left[0.0253, 0.0357\right], \\ \left[0.5309, 0.5385\right]\end{array}\right)$$	$$\left(\begin{array}{c}\left[0.0136, 0.0361\right], \\ \left[0.6956, 0.7135\right]\end{array}\right)$$	$$\left(\begin{array}{c}\left[0.0436, 0.0583\right], \\ \left[0.6256, 0.6435\right]\end{array}\right)$$	$$\left(\begin{array}{c}\left[0.0236, 0.0361\right], \\ \left[0.6152, 0.6232\right]\end{array}\right)$$	$$\left(\begin{array}{c}\left[0.0253, 0.0357\right], \\ \left[0.5809, 0.6085\right]\end{array}\right)$$	$$\left(\begin{array}{c}\left[0.0463, 0.0693\right], \\ \left[0.9109, 0.9385\right]\end{array}\right)$$	$$\left(\begin{array}{c}\left[0.0336, 0.0561\right], \\ \left[0.6422, 0.6432\right]\end{array}\right)$$	$$\left(\begin{array}{c}\left[0.0142, 0.0249\right], \\ \left[0.5267, 0.6172\right]\end{array}\right)$$

**Table 7:** Weighted decision matrix for ${\overline{\mathfrak{T}}}^{\left( 3 \right)}$.

	$\hat{\varsigma }_{1}$	$\hat{\varsigma }_{2}$	$\hat{\varsigma }_{3}$	$\hat{\varsigma }_{4}$	$\hat{\varsigma }_{5}$	$\hat{\varsigma }_{6}$	$\hat{\varsigma }_{7}$	$\hat{\varsigma }_{8}$
${\mathcal{H}}_{1}$	$$\left(\begin{array}{c}\left[0.0159, 0.0227\right],\\ \left[0.9301, 0.9693\right]\end{array}\right)$$	$$\left(\begin{array}{c}\left[0.0469 0.0613\right], \\ \left[0.9189, 0.9387\right]\end{array}\right)$$	$$\left(\begin{array}{c}\left[0.0265 0.0469\right],\\ \left[0.9189, 0.9387\right]\end{array}\right)$$	$$\left(\begin{array}{c}\left[0.0133, 0.0189\right], \\ \left[0.9414, 0.9559\right]\end{array}\right)$$	$$\left(\begin{array}{c}\left[0.0047, 0.0099\right], \\ \left[0.9693, 0.9900\right]\end{array}\right)$$	$$\left(\begin{array}{c}\left[0.0103, 0.0239\right], \\ \left[0.9661, 0.9761\right]\end{array}\right)$$	$$\left(\begin{array}{c}\left[0.0033, 0.0053\right], \\ \left[0.9661, 0.9924\right]\end{array}\right)$$	$$\left(\begin{array}{c}\left[0.0039, 0.0189\right], \\ \left[0.9414, 0.9743\right]\end{array}\right)$$
${\mathcal{H}}_{2}$	$$\left(\begin{array}{c}\left[0.0242, 0.0451\right], \\ \left[0.5867, 0.6327\right]\end{array}\right)$$	$$\left(\begin{array}{c}\left[0.0214, 0.0394\right], \\ \left[0.5876, 0.6291\right]\end{array}\right)$$	$$\left(\begin{array}{c}\left[0.0363, 0.0439\right], \\ \left[0.5814, 0.6258\right]\end{array}\right)$$	$$\left(\begin{array}{c}\left[0.0236, 0.0316\right], \\ \left[0.5961, 0.6423\right]\end{array}\right)$$	$$\left(\begin{array}{c}\left[0.0263, 0.0416\right], \\ \left[0.5924, 0.6123\right]\end{array}\right)$$	$$\left(\begin{array}{c}\left[0.0363, 0.0416\right], \\ \left[0.6465, 0.7053\right]\end{array}\right)$$	$$\left(\begin{array}{c}\left[0.0253, 0.0375\right], \\ \left[0.5816, 0.6458\right]\end{array}\right)$$	$$\left(\begin{array}{c}\left[0.0363, 0.0438\right], \\ \left[0.6365, 0.7053\right]\end{array}\right)$$
${\mathcal{H}}_{3}$	$$\left(\begin{array}{c}\left[0.0071, 0.0102\right], \\ \left[0.9683, 0.9862\right]\end{array}\right)$$	$$\left(\begin{array}{c}\left[0.0247, 0.0474\right], \\ \left[0.9379, 0.9526\right]\end{array}\right)$$	$$\left(\begin{array}{c}\left[0.0053, 0.0177\right], \\ \left[0.9227, 0.9552\right]\end{array}\right)$$	$$\left(\begin{array}{c}\left[0.0238, 0.0317\right], \\ \left[0.9549, 0.9683\right]\end{array}\right)$$	$$\left(\begin{array}{c}\left[0.0474, 0.0808\right], \\ \left[0.8511, 0.8935\right]\end{array}\right)$$	$$\left(\begin{array}{c}\left[0.0063, 0.0133\right], \\ \left[0.9788, 0.9867\right]\end{array}\right)$$	$$\left(\begin{array}{c}\left[0.0252, 0.0448\right],\\ \left[0.9227, 0.9552\right]\end{array}\right)$$	$$\left(\begin{array}{c}\left[0.0535, 0.0921\right], \\ \left[0.8709, 0.9079\right]\end{array}\right)$$
${\mathcal{H}}_{4}$	$$\left(\begin{array}{c}\left[0.0172, 0.0297\right], \\ \left[0.9441, 0.9703\right]\end{array}\right)$$	$$\left(\begin{array}{c}\left[0.0507, 0.0664\right], \\ \left[0.8414, 0.9137\right]\end{array}\right)$$	$$\left(\begin{array}{c}\left[0.0220, 0.0314\right], \\ \left[0.9443, 0.9686\right]\end{array}\right)$$	$$\left(\begin{array}{c}\left[0.0127, 0.0172\right], \\ \left[0.9703, 0.9828\right]\end{array}\right)$$	$$\left(\begin{array}{c}\left[0.0264, 0.0507\right], \\ \left[0.9336, 0.9493\right]\end{array}\right)$$	$$\left(\begin{array}{c}\left[0.0138, 0.0557\right], \\ \left[0.9043, 0.9275\right]\end{array}\right)$$	$$\left(\begin{array}{c}\left[0.0588, 0.0770\right],\\ \left[0.8686, 0.9000\right]\end{array}\right)$$	$$\left(\begin{array}{c}\left[0.0307, 0.0588\right],\\ \left[0.6687, 0.9229\right]\end{array}\right)$$

**Table 8:** Weighted decision matrix for ${\overline{\mathfrak{T}}}^{\left( 4 \right)}$.

	$\hat{\varsigma }_{1}$	$\hat{\varsigma }_{2}$	$\hat{\varsigma }_{3}$	$\hat{\varsigma }_{4}$	$\hat{\varsigma }_{5}$	$\hat{\varsigma }_{6}$	$\hat{\varsigma }_{7}$	$\hat{\varsigma }_{8}$
${\mathcal{H}}_{1}$	$$\left(\begin{array}{c}\left[0.0218, 0.0327\right], \\ \left[0.9549, 0.9683\right]\end{array}\right)$$	$$\left(\begin{array}{c}\left[0.0227, 0.0272\right], \\ \left[0.9713, 0.9728\right]\end{array}\right)$$	$$\left(\begin{array}{c}\left[0.0238, 0.0547\right], \\ \left[0.9143, 0.9265\right]\end{array}\right)$$	$$\left(\begin{array}{c}\left[0.0272, 0.0291\right], \\ \left[0.9451, 0.9733\right]\end{array}\right)$$	$$\left(\begin{array}{c}\left[0.0159, 0.0227\right],\\ \left[0.9421, 0.9573\right]\end{array}\right)$$	$$\left(\begin{array}{c}\left[0.0237, 0.0478\right], \\ \left[0.9379, 0.9526\right]\end{array}\right)$$	$$\left(\begin{array}{c}\left[0.0454, 0.0818\right], \\ \left[0.8612, 0.8975\right]\end{array}\right)$$	$$\left(\begin{array}{c}\left[0.0057, 0.0097\right], \\ \left[0.9631, 0.9906\right]\end{array}\right)$$
${\mathcal{H}}_{2}$	$$\left(\begin{array}{c}\left[0.0254, 0.0451\right], \\ \left[0.4621, 0.5279\right]\end{array}\right)$$	$$\left(\begin{array}{c}\left[0.0175, 0.0534\right],\\ \left[0.6941, 0.7981\right]\end{array}\right)$$	$$\left(\begin{array}{c}\left[0.0448, 0.0587\right], \\ \left[0.6356, 0.6445\right]\end{array}\right)$$	$$\left(\begin{array}{c}\left[0.0472, 0.0476\right], \\ \left[0.5463, 0.5762\right]\end{array}\right)$$	$$\left(\begin{array}{c}\left[0.0175, 0.0534\right],\\ \left[0.6941, 0.7981\right]\end{array}\right)$$	$$\left(\begin{array}{c}\left[0.0172, 0.0239\right], \\ \left[0.5287, 0.6162\right]\end{array}\right)$$	$$\left(\begin{array}{c}\left[0.0134, 0.0219\right],\\ \left[0.7142, 0.7235\right]\end{array}\right)$$	$$\left(\begin{array}{c}\left[0.0334, 0.0367\right], \\ \left[0.6549, 0.6878\right]\end{array}\right)$$
${\mathcal{H}}_{3}$	$$\left(\begin{array}{c}\left[0.0142, 0.0549\right], \\ \left[0.5867, 0.6872\right]\end{array}\right)$$	$$\left(\begin{array}{c}\left[0.0336, 0.0561\right], \\ \left[0.6322, 0.6431\right]\end{array}\right)$$	$$\left(\begin{array}{c}\left[0.0153, 0.0351\right], \\ \left[0.5809, 0.6085\right]\end{array}\right)$$	$$\left(\begin{array}{c}\left[0.0136, 0.0561\right], \\ \left[0.6256, 0.7135\right]\end{array}\right)$$	$$\left(\begin{array}{c}\left[0.0231, 0.0578\right], \\ \left[0.6249, 0.7129\right]\end{array}\right)$$	$$\left(\begin{array}{c}\left[0.0191, 0.0540\right], \\ \left[0.6146, 0.6372\right]\end{array}\right)$$	$$\left(\begin{array}{c}\left[0.0375, 0.0379\right], \\ \left[0.6373, 0.6852\right]\end{array}\right)$$	$$\left(\begin{array}{c}\left[0.0136, 0.0561\right], \\ \left[0.6256, 0.7135\right]\end{array}\right)$$
${\mathcal{H}}_{4}$	$$\left(\begin{array}{c}\left[0.0189, 0.0257\right],\\ \left[0.9559, 0.9662\right]\end{array}\right)$$	$$\left(\begin{array}{c}\left[0.0572, 0.0676\right], \\ \left[0.5863, 0.5960\right]\end{array}\right)$$	$$\left(\begin{array}{c}\left[0.0053, 0.0177\right], \\ \left[0.9416, 0.9823\right]\end{array}\right)$$	$$\left(\begin{array}{c}\left[0.0235, 0.0497\right], \\ \left[0.5863, 0.6771\right]\end{array}\right)$$	$$\left(\begin{array}{c}\left[0.0063, 0.0133\right], \\ \left[0.9698, 0.9867\right]\end{array}\right)$$	$$\left(\begin{array}{c}\left[0.0294, 0.0537\right], \\ \left[0.6149, 0.6578\right]\end{array}\right)$$	$$\left(\begin{array}{c}\left[0.0572, 0.0676\right], \\ \left[0.5863, 0.5960\right]\end{array}\right)$$	$$\left(\begin{array}{c}\left[0.0294, 0.0537\right], \\ \left[0.6149, 0.6578\right]\end{array}\right)$$

Step 4. Employed Eqs.( 5.5 and 5.6) to compute the PIA and NIA, respectively.

$${{\Delta }_{\widehat{{ \varsigma }_{ij}}}^{(z)}}^{+}=\left[\begin{array}{cccccccc}\left(\begin{array}{c}\left[0.0159, 0.0227\right],\\ \left[0.9301, 0.9693\right]\end{array}\right)& \left(\begin{array}{c}\left[0.0227, 0.0272\right], \\ \left[0.9713, 0.9728\right]\end{array}\right)& \left(\begin{array}{c}\left[0.0361, 0.0452\right], \\ \left[0.4953, 0.5271\right]\end{array}\right)& \left(\begin{array}{c}\left[0.0375, 0.0379\right], \\ \left[0.6373, 0.6852\right]\end{array}\right)& \left(\begin{array}{c}\left[0.0159, 0.0227\right],\\ \left[0.9421, 0.9573\right]\end{array}\right)& \left(\begin{array}{c}\left[0.0344, 0.0419\right],\\ \left[0.7245, 0.7532\right]\end{array}\right)& \left(\begin{array}{c}\left[0.0472, 0.0476\right], \\ \left[0.5763, 0.5860\right]\end{array}\right)& \left(\begin{array}{c}\left[0.0057, 0.0097\right], \\ \left[0.9631, 0.9906\right]\end{array}\right)\\ \left(\begin{array}{c}\left[0.0432, 0.0477\right], \\ \left[0.5534, 0.5748\right]\end{array}\right)& \left(\begin{array}{c}\left[0.0235, 0.0379\right], \\ \left[0.5936, 0.6517\right]\end{array}\right)& \left(\begin{array}{c}\left[0.0363, 0.0439\right], \\ \left[0.5814, 0.6258\right]\end{array}\right)& \left(\begin{array}{c}\left[0.0472, 0.0476\right], \\ \left[0.5463, 0.5762\right]\end{array}\right)& \left(\begin{array}{c}\left[0.0357, 0.0397\right], \\ \left[0.5437, 0.6725\right]\end{array}\right)& \left(\begin{array}{c}\left[0.0363, 0.0416\right], \\ \left[0.6465, 0.7053\right]\end{array}\right)& \left(\begin{array}{c}\left[0.0134, 0.0219\right],\\ \left[0.7142, 0.7235\right]\end{array}\right)& \left(\begin{array}{c}\left[0.0334, 0.0367\right], \\ \left[0.6549, 0.6878\right]\end{array}\right)\\ \left(\begin{array}{c}\left[0.0071, 0.0102\right], \\ \left[0.9683, 0.9862\right]\end{array}\right)& \left(\begin{array}{c}\left[0.0363, 0.0438\right], \\ \left[0.6365, 0.7053\right]\end{array}\right)& \left(\begin{array}{c}\left[0.0053, 0.0177\right], \\ \left[0.9227, 0.9552\right]\end{array}\right)& \left(\begin{array}{c}\left[0.0238, 0.0317\right], \\ \left[0.9549, 0.9683\right]\end{array}\right)& \left(\begin{array}{c}\left[0.0363, 0.0416\right], \\ \left[0.6465, 0.7053\right]\end{array}\right)& \left(\begin{array}{c}\left[0.0063, 0.0133\right], \\ \left[0.9788, 0.9867\right]\end{array}\right)& \left(\begin{array}{c}\left[0.0375, 0.0379\right], \\ \left[0.6373, 0.6852\right]\end{array}\right)& \left(\begin{array}{c}\left[0.0432, 0.0477\right], \\ \left[0.5534, 0.5748\right]\end{array}\right)\\ \left(\begin{array}{c}\left[0.0427, 0.0567\right], \\ \left[0.5836, 0.5906\right]\end{array}\right)& \left(\begin{array}{c}\left[0.0572, 0.0676\right], \\ \left[0.5863, 0.5960\right]\end{array}\right)& \left(\begin{array}{c}\left[0.0053, 0.0177\right], \\ \left[0.9416, 0.9823\right]\end{array}\right)& \left(\begin{array}{c}\left[0.0236, 0.0361\right], \\ \left[0.6152, 0.6232\right]\end{array}\right)& \left(\begin{array}{c}\left[0.0063, 0.0133\right], \\ \left[0.9698, 0.9867\right]\end{array}\right)& \left(\begin{array}{c}\left[0.0432, 0.0477\right], \\ \left[0.5534, 0.5748\right]\end{array}\right)& \left(\begin{array}{c}\left[0.0572, 0.0676\right], \\ \left[0.5863, 0.5960\right]\end{array}\right)& \left(\begin{array}{c}\left[0.0142, 0.0249\right], \\ \left[0.5267, 0.6172\right]\end{array}\right)\end{array}\right]$$

$${{\Delta }_{\widehat{{ \varsigma }_{ij}}}^{\left(z\right)}}^{-}=\left[\begin{array}{cccccccc}\left(\begin{array}{c}\left[0.0456, 0.0697\right], \\ \left[0.4621, 0.5069\right]\end{array}\right)& \left(\begin{array}{c}\left[0.0235, 0.0397\right], \\ \left[0.5963, 0.6171\right]\end{array}\right)& \left(\begin{array}{c}\left[0.0174, 0.0539\right],\\ \left[0.6945, 0.7962\right]\end{array}\right)& \left(\begin{array}{c}\left[0.0241, 0.0371\right], \\ \left[0.5231, 0.5472\right]\end{array}\right)& \left(\begin{array}{c}\left[0.0512, 0.0576\right], \\ \left[0.6163, 0.6260\right]\end{array}\right)& \left(\begin{array}{c}\left[0.0237, 0.0478\right], \\ \left[0.9379, 0.9526\right]\end{array}\right)& \left(\begin{array}{c}\left[0.0454, 0.0818\right], \\ \left[0.8612, 0.8975\right]\end{array}\right)& \left(\begin{array}{c}\left[0.0039, 0.0189\right], \\ \left[0.9414, 0.9743\right]\end{array}\right)\\ \left(\begin{array}{c}\left[0.0432, 0.0477\right], \\ \left[0.5534, 0.5748\right]\end{array}\right)& \left(\begin{array}{c}\left[0.0175, 0.0534\right],\\ \left[0.6941, 0.7981\right]\end{array}\right)& \left(\begin{array}{c}\left[0.0314, 0.0491\right],\\ \left[0.6754, 0.7323\right]\end{array}\right)& \left(\begin{array}{c}\left[0.0427, 0.0567\right], \\ \left[0.5836, 0.5906\right]\end{array}\right)& \left(\begin{array}{c}\left[0.0175, 0.0534\right],\\ \left[0.6941, 0.7981\right]\end{array}\right)& \left(\begin{array}{c}\left[0.0314, 0.0491\right],\\ \left[0.6754, 0.7323\right]\end{array}\right)& \left(\begin{array}{c}\left[0.0327, 0.0467\right], \\ \left[0.5836, 0.6106\right]\end{array}\right)& \left(\begin{array}{c}\left[0.0349, 0.0473\right], \\ \left[0.5749, 0.6387\right]\end{array}\right)\\ \left(\begin{array}{c}\left[0.0142, 0.0549\right], \\ \left[0.5867, 0.6872\right]\end{array}\right)& \left(\begin{array}{c}\left[0.0247, 0.0474\right], \\ \left[0.9379, 0.9526\right]\end{array}\right)& \left(\begin{array}{c}\left[0.0214, 0.0394\right], \\ \left[0.5876, 0.6291\right]\end{array}\right)& \left(\begin{array}{c}\left[0.0136, 0.0561\right], \\ \left[0.6256, 0.7135\right]\end{array}\right)& \left(\begin{array}{c}\left[0.0474, 0.0808\right], \\ \left[0.8511, 0.8935\right]\end{array}\right)& \left(\begin{array}{c}\left[0.0191, 0.0540\right], \\ \left[0.6146, 0.6372\right]\end{array}\right)& \left(\begin{array}{c}\left[0.0252, 0.0448\right],\\ \left[0.9227, 0.9552\right]\end{array}\right)& \left(\begin{array}{c}\left[0.0535, 0.0921\right], \\ \left[0.8709, 0.9079\right]\end{array}\right)\\ \left(\begin{array}{c}\left[0.0427, 0.0567\right], \\ \left[0.5836, 0.5906\right]\end{array}\right)& \left(\begin{array}{c}\left[0.0136, 0.0361\right], \\ \left[0.6956, 0.7135\right]\end{array}\right)& \left(\begin{array}{c}\left[0.0242, 0.0451\right], \\ \left[0.5867, 0.6327\right]\end{array}\right)& \left(\begin{array}{c}\left[0.0235, 0.0497\right], \\ \left[0.5863, 0.6771\right]\end{array}\right)& \left(\begin{array}{c}\left[0.0264, 0.0507\right], \\ \left[0.9336, 0.9493\right]\end{array}\right)& \left(\begin{array}{c}\left[0.0138, 0.0557\right], \\ \left[0.9043, 0.9275\right]\end{array}\right)& \left(\begin{array}{c}\left[0.0174, 0.0539\right],\\ \left[0.6945, 0.7962\right]\end{array}\right)& \left(\begin{array}{c}\left[0.0307, 0.0588\right],\\ \left[0.6687, 0.9229\right]\end{array}\right)\end{array}\right]$$

Step 5. Determine the CC among ${\overline{\mathfrak{T}}}^{\left( z \right)}$ and PIA $\Delta_{{\widehat{{\varsigma_{ij} }}}}^{{\left( z \right)+ }}$ using Eq. (17).

$$\kappa^{\left( 1 \right)} = 0.99241$$, $$\kappa^{\left( 2 \right)} = 0.99537$$, $$\kappa^{\left( 3 \right)} = 0.99327$$, $$\kappa^{\left( 4 \right)} = 0.99245$$.

Step 6. Determine the CC among ${\overline{\mathfrak{T}}}^{\left( z \right)}$ and PIA $\Delta_{{\widehat{{\varsigma_{ij} }}}}^{{\left( z \right) - }}$ using Eq. (18).

$$\tau^{\left( 1 \right)} = 0.99362$$, $$\tau^{\left( 2 \right)} = 0.99402$$, $$\tau^{\left( 3 \right)} = 0.99319$$, $$\tau^{\left( 4 \right)} = 0.99724$$.

Step 7. Compute the closeness coefficient using Eq. (19).

$${\beth }^{\left( 1 \right)} = 0.45669$$, $${\beth }^{\left( 2 \right)} = 0.56362$$, $${\beth }^{\left( 3 \right)} = 0.50295$$, $${\beth }^{\left( 4 \right)} = 0.26770$$.

Step 8. The values of the closeness coefficient ${\mathfrak{T}}^{\left( 2 \right)}$ is maximum, which shows that ${\mathfrak{T}}^{\left( 2 \right)}$ the most critical patient. The above calculation represents the most affected patient by COVID-19, is ${\mathfrak{T}}^{\left( 2 \right)}$. So the patient ${\mathfrak{T}}^{\left( 2 \right)}$ is the most deserved patient for bed.

Step 9. Patients ranking ${\mathfrak{T}}^{\left( 2 \right)} > {\mathfrak{T}}^{\left( 3 \right)} > {\mathfrak{T}}^{\left( 1 \right)} > {\mathfrak{T}}^{\left( 4 \right)}$.

## Discussion and comparative analysis

In this section, we will compare the proposed method with other commonly used approaches and discuss their strengths and weaknesses to determine the effectiveness of the presented method.

### Supremacy of the planned approach

This study offers a methodology to tackle the challenges of implementing fair bed allocation policies during the COVID-19 pandemic. We suggest a correlations-based TOPSIS method to explain MADM obstacles under IVPFHSS. Compared to existing approaches, the proposed method is more accurate and compatible in dealing with MADM concerns. It is also versatile and easy to understand, allowing for unlike outcomes with inconsistency, liability, and amendment. The proposed method can be modified for different models with vibrant ordering features to contest their opinions. Structural studies and estimates were conducted to demonstrate that the proposed methodology's consequences are comparable to those of hybrids. The proposed method is also flexible, allowing frequent FS, IFS, and PFS structures to be converted into special environments of IVPFHSS by collecting certain settings. Object-related details can be explicitly selected and tentatively, making it suitable for merging imprecise and vague figures in DM plans. Through their research and valuation, the authors found that the planned scheme's outcomes are more communal than any offered organization. Still, they noted that the projected DM enlargement involves a massive breadth of realities to relieve distress in the statistics than presented DM systems. The proposed structure is effective, flexible, and convenient, superior to other FS, IFS, and PFS hybrid structures. Furthermore, adding suitable affinities can crack some consolidation developments of IVPFS into IVPFHSS. Adding infrequent and imprecise certainties to the present actual platform is unexpected. Now, details roughly well-being can be nominated methodically and accurately. Over the DM procedure, imaginary and alarming details are cooperative. So, the intended structure would be enhanced, significant, surprising, and compound mixture FS settings. Table 9 demonstrates the features of the established and predominant techniques, clearly comparing the proposed method with existing ones. The proposed methodology offers a promising solution to the challenges in implementing fair bed allocation policies during the COVID-19 pandemic, and its flexibility and adaptability make it a valuable tool for various DM agendas.

**Table 9:** Sensitivity analysis of the planned model with prevailing models.

	Set	Parameters	Sub-parameters	Aggregated information in interval form	Advantages	Limitations
Zadeh^1^	FS	×	×	×	Deals uncertainty using MD	Unable to handle NMD
Turksen^2^	IVFS	×	×	✓	Deals uncertainty using MD intervals	Unable to handle NMD interval
Atanassov^5^	IFS	×	×	×	Deals uncertainty using MD and NMD	Unable to handle $MD + NMD > 1$
Atanassov^7^	IVIFS	×	×	✓	Deals uncertainty using MD and NMD intervals	Unable to handle $MD + NMD > 1$
Maji et al.^20^	FSS	✓	×	×	Deals uncertainty using MD	Unable to handle NMD
Maji et al.^21^	IFSS	✓	×	×	Deals uncertainty using MD and NMD	Unable to handle $MD + NMD > 1$
Jiang et al.^23^	IVIFSS	✓	×	✓	Deals uncertainty using MD and NMD intervals	Unable to handle $MD + NMD > 1$
Smarandache^38^	IFHSS	✓	✓	×	Deals uncertainty using MD and NMD	Unable to handle $MD + NMD > 1$
Debnath^42^	IVIFHSS	✓	✓	✓	Deals uncertainty using MD and NMD intervals	Unable to handle $MD + NMD > 1$
Yager^12^	PFS	×	×	×	Deals uncertainty using MD and NMD	Unable to handle $MD^{2} + NMD^{2} > 1$
Peng & Yang^17^	IVPFS	×	×	✓	Deals uncertainty using MD and NMD intervals	Unable to handle $\left( {MD } \right)^{2} + \left( {NMD } \right)^{2} > 1$
Peng et al.^28^	PFSS	✓	×	×	Deals uncertainty using MD and NMD	Unable to handle $MD^{2} + NMD^{2} > 1$
Zulqarnain et al.^37^	IVPFSS	✓	×	✓	Deals uncertainty using MD and NMD intervals	Unable to handle $\left( {MD } \right)^{2} + \left( {NMD } \right)^{2} > 1$
Zulqarnainet al.^44^	PFHSS	✓	✓	×	Deals uncertainty using MD and NMD	Unable to handle $MD^{2} + NMD^{2} > 1$
Proposed approach	IVPFHSS	✓	✓	✓	Deals uncertainty using MD and NMD intervals	Unable to handle $\left( {MD } \right)^{2} + \left( {NMD } \right)^{2} > 1$

The main reason for developing an innovative TOPSIS method is to address the limitations of existing methods. The planned methodology is unique because it is based on the IVPFHSS, which allows for a more thorough evaluation of the sub-attributes since both the MD and NMD. Previous hybrid structures such as FS, IVFS, IFS, IVIFS, PFS, IVPFS, FSS, IVFSS, IFSS, IVIFSS, IFHSS, IVIFHSS, PFSS, IVPFSS, and PFHSS were found to be insufficient in delivering comprehensive facts about the state. The proposed correlation-based TOPSIS method for IVPFHSS offers a more accurate and precise approach to resolving MADM complications. Moreover, this method is more versatile and adaptable, enabling it to cater to different situations with varying levels of disparity, guilt, and modification. The research shows that the anticipated technique's outcomes are more comprehensive and efficient than the existing methods. Table 9 illustrates the superiority of the proposed hybrid structure of FS over other hybrid systems, emphasizing its significant potential in the field. The proposed methodology has enormous potential in various applications, including organizational studies, medical research, and decision-making processes that require integrating inaccurate and imprecise data. The planned methodology significantly advances decision-making under IVPFHSS, offering a more efficient and effective way to tackle MADM obstacles. The research demonstrates that the proposed method is superior to existing methods and has enormous potential for various applications. But, in some severe circumstances, the developed TOPSIS model is unable to handle the situation, such as $\left( {MD^{\upsilon } } \right)^{2} + \left( {NMD^{\upsilon } } \right)^{2} > 1$.

### Comparative studies

After reviewing various research studies, it has been found that the correlation-based TOPSIS technique established in this study is comparable to existing designs. However, the main advantage of this method is that it considers additional information related to the sub-parameters of the alternatives to deal with uncertainties in the statistics. This method can provide more precise and more empirical object-related facts, making it helpful in defining incorrect and enigmatic realities in the DM practice. Comparative analysis shows that previous methods did not have the same intention progression in scheduling techniques because of the PIA and NIA obligations. In the developed TOPSIS model, PIA and NIA are measured founded on formative CC incentive at an assumed auxiliary level, which reduces the loss of facts in the process. The benefit of this method is that it communicates not only the degree of sensitivity but also the degree of correspondence between interpretations, thereby avoiding conclusions built on destructive bases. The proposed TOPSIS model is more efficient than recent techniques and related measures because it considers the degree of perception and similarity among clarifications. This method also deals with concerns related to the parameterization of alternatives and the handling of multi-sub-attributes, which other TOPSIS methodologies cannot handle. The comparison consequences spectacle that the finest choice of the proposed method is consistent with the established scheme, demonstrating the proposed method's reliability and productivity. The proposed correlation-based TOPSIS method is a valuable addition to the DM toolbox, as it provides more accurate and reliable results than previous techniques when dealing with uncertainty and multi-sub-attributes. In the context of TOPSIS methods, it has been observed that existing methods, such as fuzzy TOPSIS^3^ and IVFS TOPSIS^4^, only consider the MD of alternatives in the form of fuzzy and interval-valued fuzzy numbers correspondingly. Other approaches, such as IFS TOPSIS^6^ and IVIFS TOPSIS^11^, consider both the MD and NMD in the ranking of the alternatives, but they cannot deal with the parameterization of alternatives. But, these TOPSIS methodologies cannot deal with when the $MD + NMD > 1$. Biswas & Sarkar^13^ and Garg^18^ developed the TOPSIS approach for PFS and IVPFS, respectively, which capably deals with the mentioned above shortcomings. Garg and Arora^22^ developed TOPSIS methods under IFSS, and Zulqarnain et al.^26^ developed TOPSIS methods under IVIFSS. However, these methods are not capable of handling sub-attributes of substitutes. Additionally, the correlation-based TOPSIS methods for PFSS^36^ also cannot take sub-attributes of reserves.

Although some TOPSIS methods have made an effort to address concerns related to handling sub-attributes of alternatives and the information in intervals forms, such as the TOPSIS technique for IVIFHSS established in^43^ and the AOs developed in^45^ under the IVPFHSS setting, these methods may not always be sufficient to address all specific circumstances. For example, the TOPSIS method in^52^ handles sub-attributes of alternatives but ignores the information in interval form, while the AOs in^45^ can take some of the drawbacks mentioned above but may not be effective in all cases. While existing AOs can capably address some of the concerns mentioned earlier, there are still particular circumstances where these operators fail to handle the problem adequately. This is where the proposed TOPSIS model comes in. The model is designed to deal with these concerns expertly, providing a more comprehensive and effective solution for ranking alternatives. To prove the efficiency of the proposed TOPSIS model, a comparison was conducted with other existing methods, and the outcome is presented in Table 10. The ranking of the top four patients (alternatives) indicates that the best option suggested by the new method aligns with the commonly used approach. This highlights the reliability and productivity of the projected technique, suggesting that it can provide accurate and reliable results in various scenarios.

**Table 10:** Comparative analysis with existing models.

Structure	Alternatives score values or closeness coefficient				Ranking
Fuzzy TOPSIS^3^	N/A				N/A
IVFS TOPSIS^4^	N/A				N/A
IFS TOPSIS^6^	N/A				N/A
IVIFS TOPSIS^11^	N/A				N/A
IFSS TOPSIS^41^	N/A				N/A
IVIFSS TOPSIS^26^	N/A				N/A
IFHSS TOPSIS^41^	N/A				N/A
IVIFHSS TOPSIS^43^	N/A				N/A
PFS TOPSIS^13^	N/A				N/A
IVPFS TOPSIS^18^	N/A				N/A
PFSS TOPSIS^36^	N/A				N/A
PFHSS TOPSIS^52^	N/A				N/A
IVPFHSWA^45^	$Sc\left( {0.05128} \right)$	$Sc\left( {0.05973} \right)$	$Sc\left( {0.05473} \right)$	$Sc\left( {0.05291} \right)$	${\mathfrak{T}}^{\left( 2 \right)} > {\mathfrak{T}}^{\left( 3 \right)} > {\mathfrak{T}}^{\left( 4 \right)} > {\mathfrak{T}}^{\left( 1 \right)}$
IVPFHSWG^45^	$Sc\left( {0.00198} \right)$	$Sc\left( {0.06137} \right)$	$Sc\left( {0.04925} \right)$	$Sc\left( {0.04362} \right)$	${\mathfrak{T}}^{\left( 2 \right)} > {\mathfrak{T}}^{\left( 3 \right)} > {\mathfrak{T}}^{\left( 4 \right)} > {\mathfrak{T}}^{\left( 1 \right)}$
Proposed TOPSIS	$0.45669$	$0.56362$	$0.50295$	$0.26770$	${\mathfrak{T}}^{\left( 2 \right)} > {\mathfrak{T}}^{\left( 3 \right)} > {\mathfrak{T}}^{\left( 1 \right)} > {\mathfrak{T}}^{\left( 4 \right)}$

**Figure 2:**
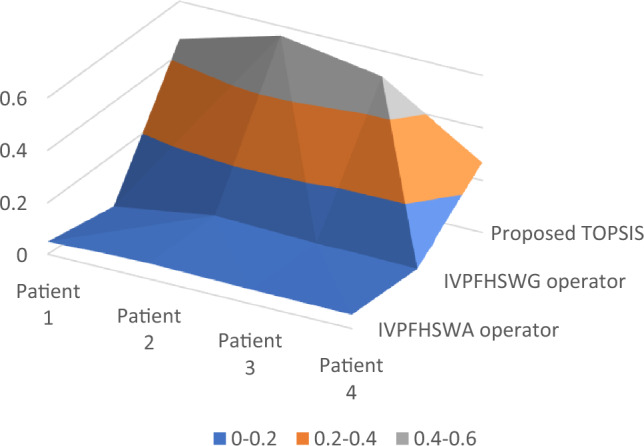
Comparative analysis with the existing models.

The planned TOPSIS model successfully addresses the limitations of other TOPSIS methods, including the inability to handle the parametrization of alternatives and the lack of consideration for sub-parameters. Additionally, it deals with information in interval form and multi-sub-attributes of alternatives, which other TOPSIS methods either ignore or cannot handle. These concerns are essential in decision-making processes, and the proposed TOPSIS model is designed to handle them expertly. The effectiveness of the proposed TOPSIS model is demonstrated by its ability to determine the most critical patient, ${\mathfrak{T}}^{\left( 2 \right)}$, who needs a bed on an emergency basis using all available alternatives (patients). The sufficiently of alternatives considered by the proposed TOPSIS method pays to a similar absolute decision as the other TOPSIS methods, indicating the consistency and efficiency of the proposed technique. The comparison results in Table 10 confirm the superiority of the proposed TOPSIS model in determining the optimal choice among the available alternatives. The proposed TOPSIS model represents a significant advancement in the field of decision-making and has the potential to make a significant impact in various industries and applications. Its ability to handle various concerns and effectively determine the best alternative among many options makes it a valuable tool for decision-makers. The graphical demonstration of comparative analysis is given in the following Fig. 2.

Several assessment standards will be applied to assess the excellence of the outcomes generated by the suggested methodology. These are a few popular ways to assess solutions’ reliability:

- Performing an analysis of sensitivity is helpful in evaluating the stability of the responses that have been collected. By altering or modifying the source information or variables, we can observe why the order of the alternatives fluctuates. A stable rating through numerous instances indicates a more satisfactory response.
- Assessing the advocated strategy’s consequences with existing standard outcomes or well-defined methods presents an overview of the efficacy of the outcomes. If the suggested technique frequently surpasses or exceeds the effectiveness of comparable methods, it signifies a high-quality strategy.
- Take confirmation and feedback from subject matter experts or decision-makers. Their expertise and experience may give significant perspectives on the overall quality of the solutions. The views of experts are able to determine whether or not the calculated ranks reflect their assumptions or desires.
- Evaluate decision-makers compliance with the responses delivered by the presented methodology. Decision-makers’ opinions and personal opinions may give a measure of the standard and applicability of alternatives in executing their specific requirements and needs.
- Case studies must be executed, or the suggested approach can be implemented in real-world situations that require decision-making. Analyze the findings and determine whether the approaches correlate with the actual efficiency or consequences in specific scenarios. Real-world executions deliver significant proof to demonstrate the technique's effectiveness and reliability.

### Advantages of the proposed model

The results obtained through the proposed approach, which integrates correlation measures with the TOPSIS technique, offer multiple benefits contributing to the technique’s effectiveness in MADM challenges. Let’s talk about these values as well as established approach performance:

- The suggested technique permits a more precise evaluation of the interactions among attributes or criteria within the IVPFHSS structure by including the correlation measures (CC and WCC). This promotes decision-making precision by identifying the fundamental associations and interactions between the different factors implicated. The improvement in sensitivity results in more reliable assessment and evaluation of decisions.
- In some cases, experts may face difficulty in making absolute choices about attributes and their associated sub-attributes. This model addresses this issue by operating the interval-valued Pythagorean fuzzy parameterization concept, which considers aspects' irregular performance and associated sub-attributes.
- The technique promotes transparency in decision-making by demonstrating a clear ranking or preference order of the alternatives. Since this approach presents a structured and monitored review system, decision-makers are able to examine and interpret the results. Integrity facilitates effective communication, collaboration, and decision making.
- The schematic TOPSIS scheme enables professionals to deliver evaluations founded on two-dimensional MD and NMD values, permitting them to approve or differ with assessments independently in the form of intervals. This approach provides greater flexibility for the experts, enabling them to express their opinions.
- This study demonstrates that the proposed approach effectively deals with intricate decision-making circumstances. This method may effectively handle unpredictability, inconsistencies, and varying information scales inside the IVPFHSS structure by using correlation measures and TOPSIS. The method's durability enables it to be applied in an extensive range of practical problems and decision scenarios, generating reliable and predictable consequences.
- The proposed TOPSIS model allows for estimates based on multiple sub-attributes rather than just a single parameter. This more efficient procedure enables experts to provide a more comprehensive and accurate assessment of the alternatives considered in the HSS environment. Overall, the proposed model is more reliable and effective than existing methods, offering greater flexibility and accuracy for experts.
- The proposed strategy is applicable to a number of sectors and industries where MADM obstacles be found, such as logistics, transportation, finance, and sustainability, among others. Its flexibility is derived from its ability to cope with various kinds of information and factors, which enables it to be effectively employed in a range of decision cases.

## Conclusion

This work presents an innovative TOPSIS approach that deals with the challenges of inadequate information, inadequacy, and inattention in the IVPFHSS setting by assessing the MD and NMD of n-tuple sub-attributes for the examined aspects. The recommended strategy has several advantages, including addressing the ambiguous nature of attributes and sub-attributes, assisting specialists in offering evaluations using 2D MD and NMD and presenting evaluations as HSS sub-attributes. The study aims to provide new correlation measures for IVPFHSS, such as CC and WCC. TOPSIS is a mechanism sub-attributes, and specialists provide to determine MADM challenges using the proposed CC and WCC. The extended correlation measures have been extensively researched and aid in determining correlation indices and closeness coefficients for evaluating the PIA (NIA) and rank of alternatives. A computational demonstration has demonstrated the suggested technique’s efficacy and uniqueness for bed allocation of the essential COVID-19 patient, and a comparative study was performed to validate its effectiveness. The intended TOPSIS model proved more credible and effective than conventional TOPSIS methodologies and AOs, illustrating its ability to assist decision-makers in the DM procedure. Future research can look into different MADM approaches, such as VIKOR and MABAC, to deal with DM challenges. Also, it may be extended to interval-valued q-rung orthopair fuzzy hypersoft sets along with its basic operations and several AOs with their distinct DM strategies. We can further integrate interval valued q-rung orthopair fuzzy hypersoft numbers with MADM, MCDM, MCGDM, and MAGDM strategies to carry out real-life applications such as material selection, diagnostics, information fusion, pattern identification, as well as supply chain management. The present study delivers a substantial improvement in the area of DM by providing an efficient TOPSIS approach that will handle various types of data unpredictability and contradiction issues.

## Data Availability

The datasets used during the current study available from the corresponding author on reasonable request.
